# GenAI-Net: A generative AI framework for automated biomolecular network design

**DOI:** 10.1126/sciadv.aeh8819

**Published:** 2026-09-30

**Authors:** Maurice Filo, Nicolò Rossi, Zhou Fang, Mustafa Khammash

**Affiliations:** ^1^ Department of Biosystems Science and Engineering, ETH Zürich, 4056 Basel, Switzerland.; ^2^ State Key Laboratory of Mathematical Sciences, Academy of Mathematics and Systems Science, Chinese Academy of Sciences, Beijing 100190, China.

## Abstract

Biomolecular networks underlie both natural biological processes and engineered cellular technologies, from intracellular regulation and ecological dynamics to biomanufacturing, smart therapeutics, and cell-based diagnostics. However, designing chemical reaction networks (CRNs) that implement a desired dynamical function remains a challenging task. Although candidate networks can be evaluated by simulation, the inverse problem of discovering networks from behavioral specifications remains difficult. It requires navigating vast spaces of topologies and kinetic parameters governed by nonlinear and potentially stochastic dynamics. Here, we introduce GenAI-Net, a generative artificial intelligence framework that automates CRN design by coupling reaction proposal to simulation-based evaluation defined by a user-specified objective. GenAI-Net efficiently produces topologically diverse solutions across design tasks, including dose-response shaping, complex logic gates, classifiers, oscillators, habituation, robust perfect adaptation, and noise reduction in stochastic settings. By turning specifications into families of circuit candidates, GenAI-Net provides a route to programmable biomolecular circuit design and accelerates translation from desired function to implementable mechanisms.

## INTRODUCTION

With modern advances in gene editing and DNA synthesis technologies (*1*–*5*), the rational engineering of living systems has become a practical reality (*6*–*11*). This capability is poised to transform diverse areas—from precision therapeutics and cell-based diagnostics to sustainable biomanufacturing and new biomaterials—with major implications for human health and the environment (*12*, *13*). A central prerequisite is the ability to design biomolecular circuits and reaction networks that implement desired functions while remaining sufficiently compact and robust to operate in complex cellular contexts. However, this design task remains difficult: Biomolecular circuits are inherently nonlinear and often nonmodular, and their function emerges from a complex interplay between reaction topology and kinetic parameters. As a result, achieving a desired behavior still typically demands substantial manual iteration and trial-and-error, reflecting the lack of a general, principled design approach.

To date, most biomolecular circuits have been designed by combining human intuition with principles from applied mathematics and classical systems and control theory. This approach enabled seminal synthetic circuits such as the repressilator (*6*), the genetic toggle switch (*7*), and synthetic autoregulation (*8*), which helped launch the field of synthetic biology. More recently, control-theoretic integral feedback has enabled the design of biomolecular circuits that achieve stringent forms of homeostasis (*9*, *14*–*22*). Proportional-integral-derivative (PID) control has also been translated into biomolecular circuit architectures to tune transient dynamics and improve performance beyond steady-state regulation (*23*–*29*). Furthermore, alternative actuation and sensing mechanisms have been explored to achieve similar improvements in dynamical response and noise suppression (*30*–*34*).

Beyond regulatory feedback motifs, biomolecular networks have been proposed for intricate dynamical responses and biochemical information processing, including minimum realizations (*35*), noise filters (*36*), linear classifiers (*37*), logic circuits (*38*–*40*), and even artificial neural networks (*41*–*49*). However, achieving these richer behaviors may require complex networks, and translating such designs to in vivo settings remains challenging—motivating automated approaches that can generate diverse candidate mechanisms and expose complexity-performance trade-offs.

Beyond human intuition, biomolecular circuits can also be designed using machine intelligence. Early work (*50*) used exhaustive enumeration to identify adaptive behaviors across all three-node enzyme network topologies. As the number of species grows and enumeration becomes infeasible, learning-based strategies become essential. Other pipelines have leveraged sparse regression (*51*), evolutionary algorithms (*52*–*55*), tree search (*56*), topological filtering (*57*), Thompson sampling (*58*), and Bayesian optimization (*59*) to discover biochemical networks with desired functions. More recently, a deep-learning approach based on conditional variational autoencoders was introduced (*60*), using simulations of three-node circuits to guide parameter search and retrieve circuits that exhibit adaptation (*60*). In a complementary direction, a supervised learning approach has been recently applied to large-scale experimental measurements to learn predictive composition-to-function models (*61*). Collectively, these methods demonstrate that machine intelligence can automate and accelerate biomolecular circuit design (*62*, *63*); however, existing approaches often remain constrained in either versatility (the range of tasks and network sizes they can handle) or efficiency (the ability to search large topology-parameter spaces without extensive task-specific engineering).

Often, the forward design direction is straightforward: Given a proposed circuit, we can usually test whether it performs a desired task (e.g., by simulating its dynamics). The reverse direction—starting from a behavioral specification and discovering a network that realizes it—is far more challenging, because the space of reaction topologies and parameterizations is vast with nonlinear and possibly stochastic dynamics. Here, we introduce GenAI-Net, a generative artificial intelligence (AI) framework that tackles this inverse problem by enabling flexible, automated design of chemical reaction networks (CRNs) for a broad range of user-defined tasks. GenAI-Net exploits the asymmetry in difficulty between the forward and reverse problems by placing an AI agent in the loop (Fig. 1). The agent acts as an oracle to automatically propose candidate designs (reverse problem) and pass them to a simulator for performance evaluation (forward problem). The resulting evaluation signals are then used to refine the oracle’s accuracy and strengthen the agent’s generative capability, which is fully encoded in its probabilistic neural network policy. In this work, therefore, generative AI refers to learning and sampling from a probability distribution over structured input-output chemical reaction network (I/O CRN) designs, rather than to the use of large language models, diffusion models, or foundation models.

**Fig. 1.**
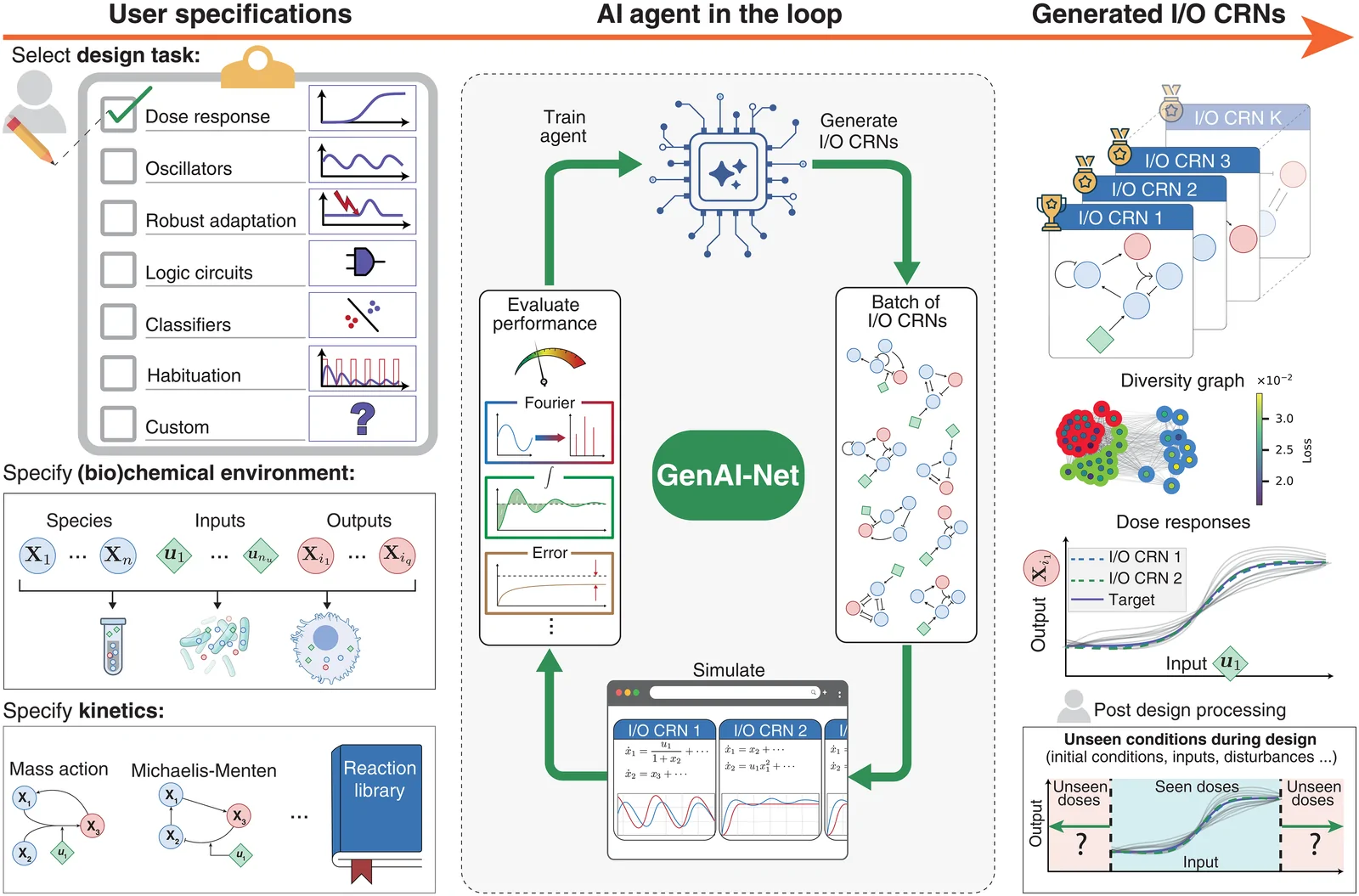
GenAI-Net overview: A generative AI agent for automatic design of I/O CRNs. User specifications: Users begin by selecting a desired design task. They then specify the intended (bio)chemical operating environment, including the set of chemical species, the designation of inputs (e.g., externally set signals $u_{1}$) and outputs (regulated/readout species), and any contextual constraints implied by the application setting. Last, users choose a kinetic model class (e.g., mass-action or Michaelis-Menten) and provide an available reaction library containing *M* permissible reactions from which candidate networks may be assembled. AI agent in the loop: Given these specifications, GenAI-Net iteratively generates candidate I/O CRNs, simulates their dynamics under the chosen kinetics, and evaluates performance against the task objective using quantitative metrics. These evaluations provide a learning signal used to train the agent, closing the design loop: The agent proposes networks conditioned on prior performance, progressively improving the generated candidates. The output of a design run is a batch of I/O CRNs sampled from the learned search policy, enabling downstream selection, analysis, and implementation. Generated I/O CRNs and behaviors: GenAI-Net returns multiple top-ranked candidate I/O CRNs, each represented by a reaction topology, parameters, and its corresponding predicted input-output behavior. Diversity graphs can be used to monitor the topological diversity of the generated I/O CRNs. The example to the right highlights how topologically distinct candidate networks can realize similar target specifications (e.g., matching a desired dose-response curve) while differing in internal reaction structure, enabling users to trade off performance, simplicity, and implementability when choosing a final design. The resulting designs can then be evaluated under conditions not used in the design loop. For example, the bottom-right panel illustrates a postdesign generalization test in which the generated I/O CRNs are simulated across dose levels that were not included during design.

A GenAI-Net user starts by specifying a task-level objective—what behavior the CRN should realize—together with the chemical context in which the design must live (Fig. 1, left). The user defines the molecular species and, when relevant, designated inputs and outputs, chooses a kinetic model (e.g., mass-action or Michaelis-Menten, deterministic or stochastic setting), and/or selects a reaction library that constrains which reaction types may be used. With these ingredients, GenAI-Net automatically generates and returns a set of diverse candidate CRNs (Fig. 1, right), enabling the user to compare alternative topologies and parameterizations and select designs that best match performance, simplicity, implementability, and even generalizability (Fig. 1, bottom right).

We apply GenAI-Net to a broad set of design specifications, including dose-response shaping (Hill-function matching, ultrasensitivity, and nonmonotonic responses), robust perfect adaptation (RPA) under setpoint changes and disturbances (both in deterministic models and in stochastic settings where noise reduction is additionally targeted), classifiers that map initial conditions to discrete fates, oscillators with fixed-mean dynamics or input-tunable frequency, logic circuits implementing complex Boolean gates, and habituation/sensitization. In each case, the specification is captured through a simulation-based performance evaluation (e.g., time-domain tracking error, classification loss over initial conditions, frequency-domain objectives, or truth-table accuracy), and GenAI-Net successfully generates diverse CRN designs that meet the desired behaviors. Together, these results position GenAI-Net as a general-purpose engine for programmable molecular circuit design, enabling rapid discovery of diverse reaction motifs directly from high-level specifications. All the code to reproduce the results of this work and to explore other applications is available in a Github repository (*64*) with an easy-to-use programmatic user interface and detailed documentation and list of generated CRNs (see Data, code, and materials availability).

## RESULTS

In what follows, we demonstrate how GenAI-Net enables automated, objective-driven discovery of CRNs across a wide range of dynamical tasks. We first present the core method—how candidate CRNs are represented, generated, and evaluated through simulation, and how this feedback is used to progressively improve design proposals. We then use this framework to produce diverse high-performing networks in multiple application settings, illustrating both the behaviors achieved and the reaction-level motifs that emerge from the search.

### Input-output chemical reaction networks

We represent each design as an I/O CRN: a collection of interacting species connected by reactions, where some quantities are treated as external inputs that modulate reaction rates and one or more species are designated as outputs of interest (to be measured or regulated). Each reaction is defined by its stoichiometry and kinetics. More precisely, a single reaction $R_{j}$ over species $\left(X_{1},\ldots ,X_{n}\right)$ is written as

$$R_{j}:\nu_{1j}X_{1}+\cdots +\nu_{\mathrm{nj}}X_{n}\overset{\lambda_{j}\left(x,u;\theta \right)}{\to }\nu_{\text{lj}}^{\prime }X_{1}+\cdots +\nu_{\text{nj}}^{\prime }X_{n}$$ (1)

where $\nu ,\nu^{\prime }$ ∈ ℕ_0_ are stoichiometric coefficients and $\lambda_{j}(x,u;\theta )$ is the reaction’s propensity (rate), which depends on the current state (species concentration or molecular counts), optional external inputs *u* that modulate reaction rates, and kinetic parameters **θ**. Each time this reaction fires, the system state changes by the net stoichiometric update $\Delta_{j}=\nu_{j}^{\prime }-\nu_{j}$. For a network with *m* reactions, stacking these **Δ***_j_* vectors as columns yields the stoichiometric matrix $S\in {\mathbb{Z}}^{n\times m}$, and stacking the propensities yields $\lambda \left(x,u;\theta \right)\in {\mathbb{R}}_{\geq 0}^{m}$. In the deterministic setting, the resulting dynamics are $\dot{x}=S\lambda (x,u;\theta )$; in the stochastic setting, the same *S* and λ define a continuous-time Markov jump process described by the chemical master equation (CME), typically simulated with the Gillespie algorithm (*65*). An I/O CRN is simply such a collection of reactions, together with a designation of which signals serve as inputs *u* and which species are treated as outputs.

### The reinforcement learning loop

The reinforcement learning (RL) loop (Fig. 2A) begins when the user specifies (i) a starter I/O CRN and (ii) the maximum number of reactions *m* that may be appended to complete the design. Together, these choices define the start of the construction process (the starter I/O CRN) and its endpoint (a completed I/O CRN obtained after *m* reaction additions).

**Fig. 2.**
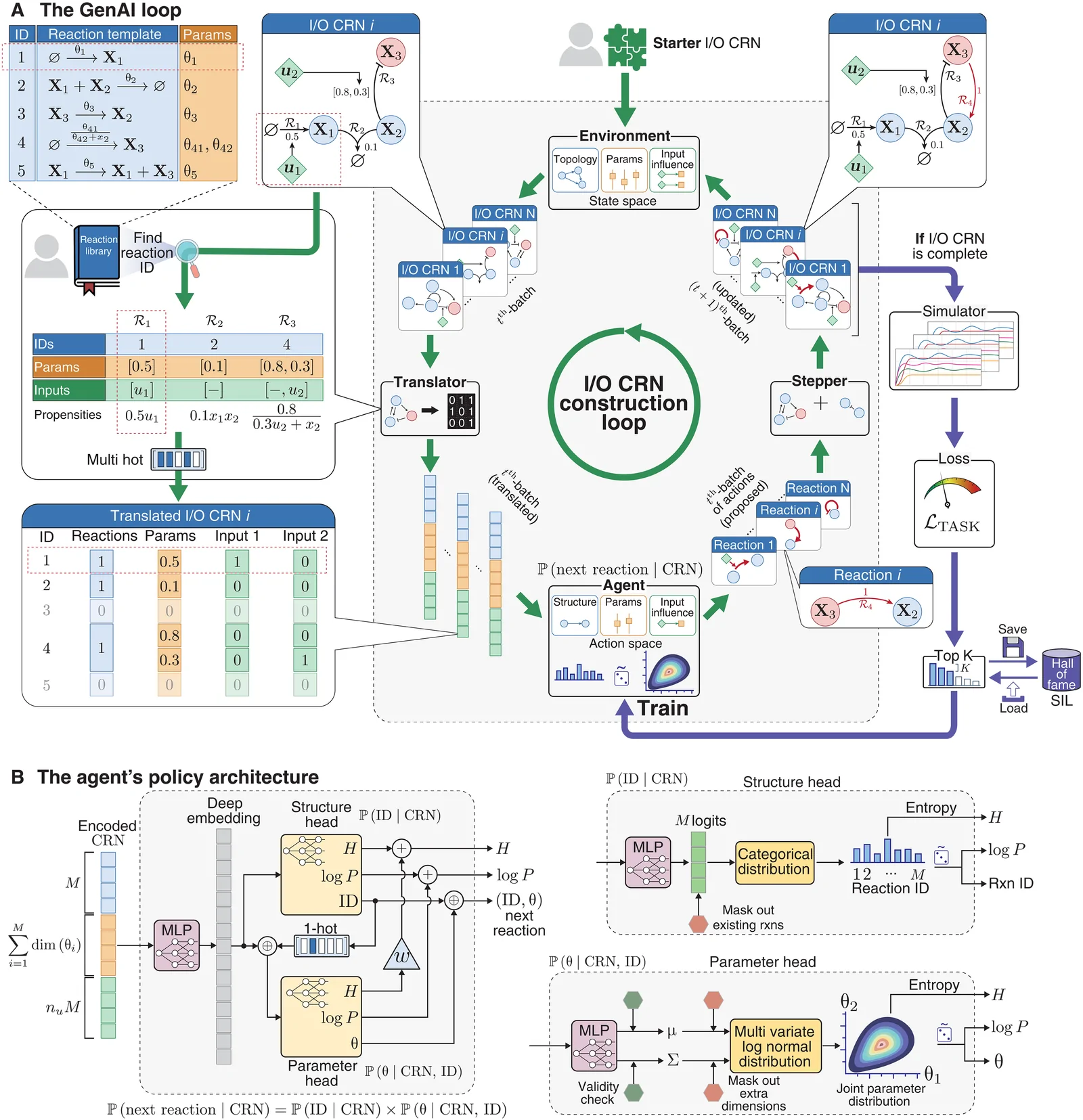
Overview of GenAI-Net. (**A**) The GenAI loop. Starting from a user-provided starter I/O CRN, GenAI-Net grows candidate networks by sequentially selecting reaction templates from a reaction library and assigning their kinetic parameters. A candidate I/O CRN (left; “I/O CRN *i*”) is represented as a set of reaction IDs and rate parameters (table), together with any inputs that modulate propensities. This representation is translated into an agent-interpretable form, where each reaction is encoded as a multihot entry indicating whether it is present, its parameter value(s), and which input influences its propensity. Conditioned on this representation, the agent proposes an action (e.g., adding a new reaction and its rate). A stepper applies the action to update the I/O CRN in the environment, and the translator is applied again. Once a terminal condition is met, the environment uses the updated I/O CRN to build its dynamical model under the chosen kinetics and passes it to the simulator and then to the loss module to evaluate the resulting trajectories and compute task-specific loss $L_{\text{TASK}}$. Across rollouts, GenAI-Net maintains a “hall of fame” of high-performing solutions and uses these elite trajectories to refine the training of the agent via SIL. (**B**) The agent’s policy architecture. The policy maps the current incomplete I/O CRN to a deep embedding, which feeds two coupled heads. The structure head produces logits over the discrete action space of reaction IDs, defining a categorical distribution ℙ(ID∣CRN); already-present reactions are masked to prevent redundant selection, and an entropy term encourages exploration during training. Given the selected reaction ID, the parameter head outputs a distribution over the continuous kinetic parameters ℙ(θ∣CRN,ID). Together, these heads define the policy ℙ(next_reaction∣CRN)=ℙ(ID∣CRN)ℙ(θ∣CRN,ID), enabling end-to-end learning of both network topology and kinetics.

At the core of GenAI-Net is an environment (top center of the construction loop in Fig. 2A) whose state is the current I/O CRN, which may be incomplete. Initially, the state is exactly the user-provided starter I/O CRN. As the loop unfolds, the state evolves through a discrete, combinatorial state space consisting of all CRNs obtainable by appending up to *m* reactions from the chosen reaction library, together with their associated kinetic parameterizations and any permitted input-modulation assignments. Thus, each state encodes the reactions selected so far (and how they are parameterized) while preserving the starter I/O CRN’s species and any designated input/output roles.

Because a CRN state is naturally expressed as a human-readable list of reactions, GenAI-Net includes a translator (center left of the construction loop in Fig. 2A) that converts the current I/O CRN into a fixed-format, agent-interpretable representation. Concretely, the translator maps the current set of selected reactions into a structured encoding that, for each library reaction, records whether it is present, its kinetic parameter value(s), and which input channels (if any) modulate its propensity. This translation standardizes variable-sized CRN descriptions into a consistent representation that the policy can condition on, while maintaining a direct correspondence back to reaction-level semantics.

Given the translated state, the agent (bottom center of the construction loop in Fig. 2A) selects the next reaction to add. The action space is defined by the reaction library: Each action corresponds to choosing a reaction identity (and, when relevant, its parameter values and input modulation choices), subject to constraints such as masking reactions already present. The agent’s decision is governed by a learned policy. Although the agent consumes the translated encoding, it produces its decision in a human-interpretable form—for example, in Fig. 2A (under proposed reaction), the agent selects the highlighted reaction (shown in red) to append next.

A lightweight stepper block (center right of the construction loop in Fig. 2A) then applies this action by appending the proposed reaction to the existing CRN, producing the next environment state; in the running example, the stepper augments the template by adding the red reaction to yield the updated I/O CRN shown in Fig. 2A (under updated I/O CRN). This construction loop repeats for *m* steps (or until a termination condition is met), yielding a complete I/O CRN. Once the I/O CRN is complete, GenAI-Net switches from the outer construction loop to an inner evaluation-and-training loop. The completed CRN is passed to a simulator, its trajectories are scored by the task objective to produce a scalar loss, and this loss provides the learning signal used to update the policy network of the agent (Fig. 2B). For a more detailed description of the various components in the RL loop, see Materials and Methods.

### GenAI-Net generates networks with target dose-responses

We first exemplify GenAI-Net for generating networks with target dose-responses. This task is prevalent in biological studies, aiming to identify the underlying biochemical mechanisms given experimentally measured dose-response curves or to generate networks exhibiting such responses for synthetic-biology applications. This problem is often hindered by the vast search space over both network structure and kinetic parameters, making exhaustive search impractical. For instance, we consider a system consisting of three chemical species and four starting reactions as shown in Fig. 3A, where the input signal regulates the production rate of one species, and the remaining degradation reactions (one for each reaction) represent the dilution process due to cell growth. Allowing up to five more doubly bimolecular mass-action reactions yields more than 3 × 10^7^ possible networks (i.e., the number of combinations of 87 candidate reactions taken five at a time), and this space expands further when accounting for choices of reaction rate constants.

**Fig. 3.**
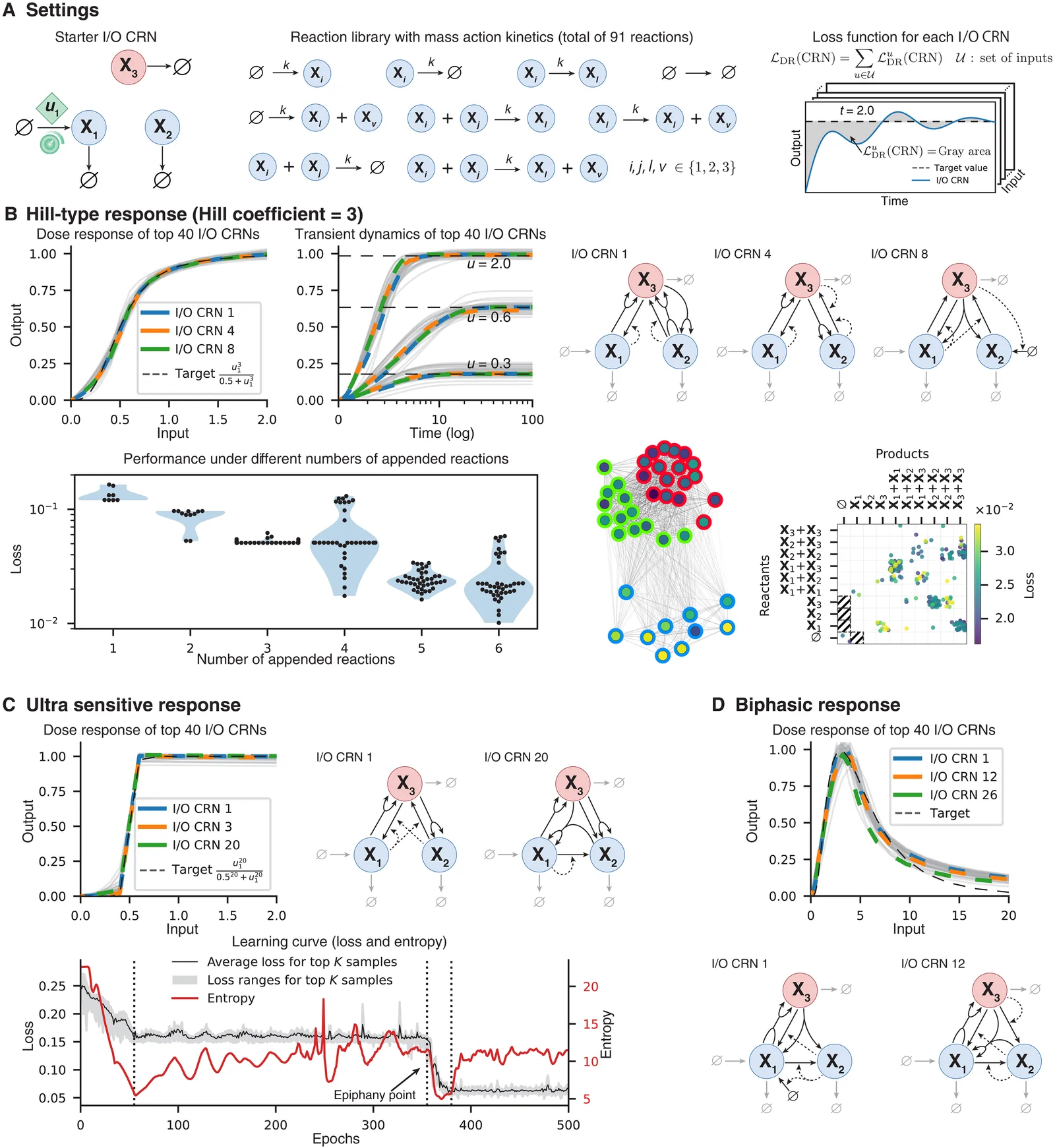
Dose response. (**A**) Setup for generating I/O CRNs exhibiting desired dose-responses. GenAI-Net starts from a starter I/O CRN of three species, including an output $X_{3}$, and four reactions. The rate of one of the reactions is modulated by an input $u_{1}$. GenAI-Net generates candidate I/O CRNs by appending up to five doubly bimolecular mass-action reactions from a library and optimizes the average loss of the top 5% generated networks together with an entropy term to boost diversity. The loss of each network is defined as a weighted $L_{1}$ norm of the difference between the output and the desired response over time, averaged across many different input conditions. (**B**) Generation of networks exhibiting Hill-type responses. GenAI-Net is capable of generating more than 40 topologically unique networks whose dose-response matches the target Hill function while also exhibiting favorable transient dynamics (first row). The colored lines represent the performance of three topologically distinct networks (top right), while the gray lines indicate the performance of the remaining networks among the top 40. The graph (middle, second row) summarizes topological diversity across the top 40 generated solutions: Nodes denote I/O CRN topologies, edge grayscale encodes pairwise Hamming distance (the number of differing reactions), node fill color indicates loss, and node outline color indicates clusters identified automatically via the Louvain method. The reactant-product incidence map (right bottom) summarizes reaction usage across the generated I/O CRNs: Each point corresponds to the reactants and their products, colored by the loss associated with I/O CRNs in which the reaction appears (color bar). The bottom-left panel illustrates how the performance of the top I/O CRNs changes with the number of appended reactions. (**C** and **D**) Generation of networks exhibiting ultrasensitive and biphasic responses. The bottom panel in (C) shows the evolution of the loss and entropy over epochs during learning.

Despite these challenges, GenAI-Net can generate a diverse set of networks that realize target dose-response curves, including Hill-type, ultrasensitive, and nonmonotonic behaviors (see Fig. 3, B to D). As shown, for each target curve, GenAI-Net identifies more than 40 topologically distinct networks whose responses closely match the desired profile. This diversity and precision arise from the balance of the loss and entropy terms of the network in the objective function. In one representative training procedure (bottom panel of Fig. 3C), the entropy term (red) drops quickly when the loss (black) decays rapidly—most notably during the first and third periods separated by dashed lines—indicating strong exploitation to select high-performing I/O CRNs from previously sampled networks. In other periods, when the loss plateaus, the entropy gradually increases, signaling exploration for alternative topologies with lower loss and thereby yielding diverse, high-quality networks. In addition to capturing the target dose-response, the generated networks exhibit smooth, fast-converging transient dynamics (second panel in the first row of Fig. 3B). This is encouraged by the loss-function design (see Materials and Methods), which evaluates trajectory-level mismatches between simulated dynamics and target values over time (right panel of Fig. 3A).

The generated networks also exhibit recurring topological patterns, highlighting specific design principles. In particular, several reactions are frequently selected by high-performing networks; whereas others are consistently avoided (see the bottom-right panel of Fig. 3B for the Hill-type response). By grouping networks according to topological similarity using the Louvain method (bottom-middle panel of Fig. 3B), we identify several representative topologies across the resulting clusters, as shown in the top-right panel of Fig. 3B. Similar patterns arise in the other dose-response examples, with representative networks shown in Fig. 3 (C and D).

The number of appended reactions is a key design knob for navigating the trade-off between topological simplicity and achievable performance. Allowing more appended reactions expands the space of candidate mechanisms and can improve the performance of the top I/O CRNs (bottom-left panel of Fig. 3B), with the best-performing network improving steadily as the reaction budget increases. This flexibility enables users to select a reaction budget that matches the desired balance between compactness and functional precision, depending on the requirements of the application.

### GenAI-Net discovers RPA circuits for setpoint tracking and disturbance rejection

One of the fundamental tasks of synthetic biomolecular feedback controllers (*66*) is to maintain homeostasis, a property with broad impact in bioproduction, metabolic engineering, and cell-based therapies, where many diseases arise from homeostatic failure (*67*, *68*). RPA (*69*–*71*) is a stringent form of homeostasis: It enforces exact steady-state regulation of a target variable to a prescribed setpoint despite persistent disturbances. A substantial body of work has developed control-theoretic CRN designs that achieve RPA or near-RPA (*15*–*17*, *30*, *72*–*77*), demonstrated experimental implementations (*9*, *14*, *19*, *20*, *22*, *78*–*80*), and provided increasingly general characterizations of RPA-capable network classes (*9*, *81*–*84*). However, even with these characterizations, the remaining design space can still be large, and satisfying structural conditions alone does not guarantee favorable transient performance.

In the following examples, we use GenAI-Net to design molecular controllers that achieve RPA with good dynamic performance (Fig. 4). We formulate RPA as an input-output control task in which an external input *u*_1_ specifies the desired setpoint and a second input *u*_2_ acts as a persistent disturbance by modulating the effective degradation of the regulated species (Fig. 4, A and B, left). With a minimal starter I/O CRN containing the regulated output species and controller species, GenAI-Net appends a user-defined number of additional reactions from the library and optimizes a trajectory-level loss (see Materials and Methods) that penalizes setpoint-tracking error over time, averaged across multiple setpoints and disturbance strengths. This objective explicitly rewards both fast and smooth dynamics and accurate steady-state regulation across conditions.

**Fig. 4.**
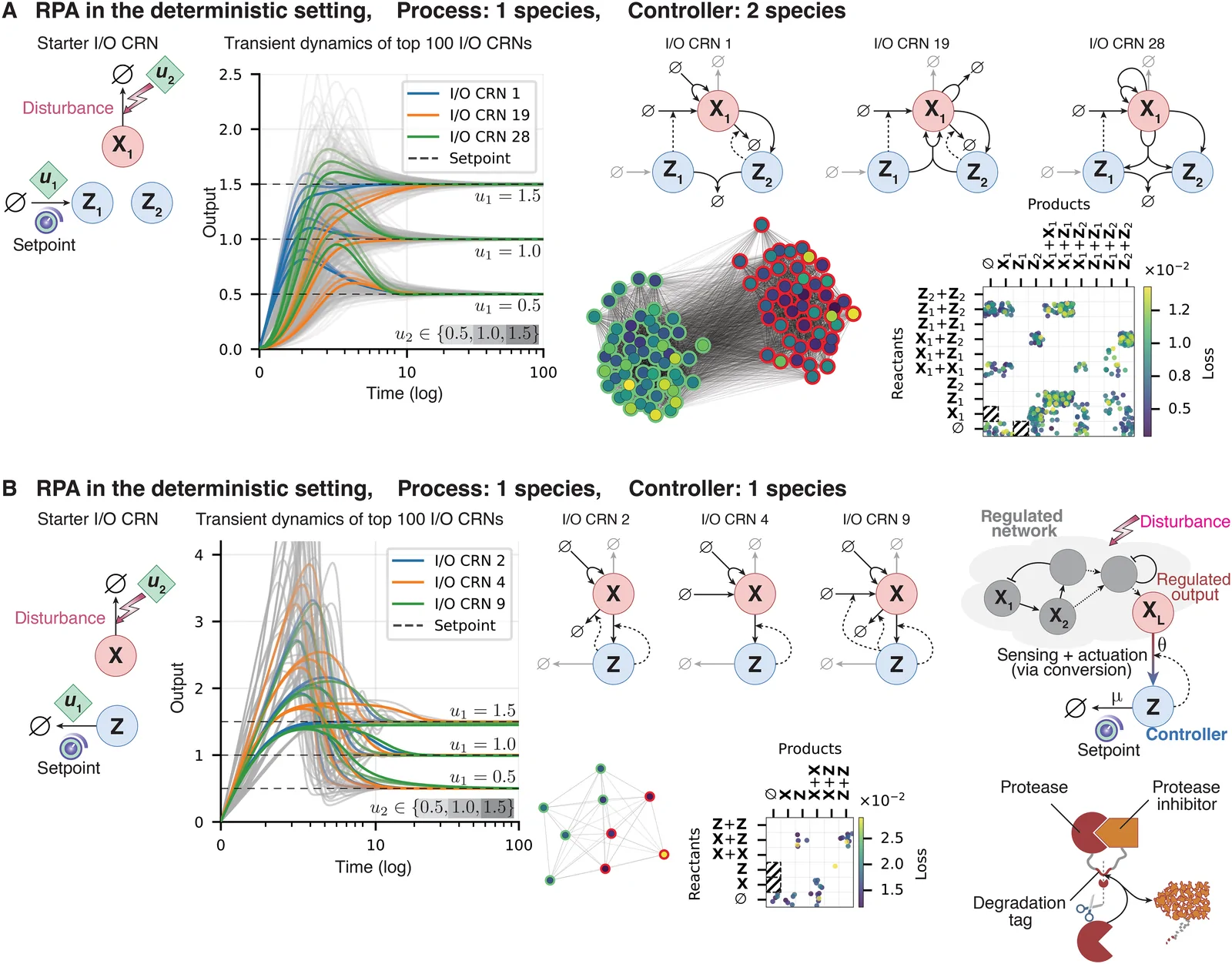
Robust perfect adaptation. (**A**) GenAI-Net begins with a starter I/O CRN consisting of three species—the regulated output $X_{1}$ and controller species $Z_{1},Z_{2}$—and two reactions whose rates are modulated by external inputs $u_{1}$ and $u_{2}$. The input $u_{1}$ tunes the desired setpoint, while $u_{2}$ acts as a disturbance. GenAI-Net generates candidate I/O CRNs by appending up to five doubly bimolecular mass-action reactions, optimizing a loss defined as a weighted $L_{1}$ norm of the setpoint-tracking error over time, averaged across three setpoints ($u_{1}\in \left\{0.5,1.0,1.5\right\}$) and three disturbance magnitudes ($u_{2}\in \left\{0.5,1.0,1.5\right\}$); see Materials and Methods. The time-course plot shows the regulated output versus log time for the top 100 topologically unique I/O CRNs (thin gray trajectories), with three representative solutions highlighted (colored curves; I/O CRNs 1, 19, and 28 shown to the right). For each highlighted I/O CRN, transparency encodes the disturbance strength set by $u_{2}$ (lighter to darker shades). All responses demonstrate robust convergence toward the desired setpoint levels (horizontal dashed lines) across all settings. The reactant-product incidence map and the topological diversity graph are shown for (A) and (B). (**B**) A minimal starter I/O CRN with a single controller species **Z** yields a smaller generated solution set (nine topologically unique I/O CRNs). Here, up to four reactions were allowed. As in (A), the center panel shows output trajectories for all generated solutions (gray), with three representative networks highlighted (I/O CRNs 2, 4, and 9) and their corresponding topologies shown to its right. The schematic to the right highlights a control motif discovered by GenAI-Net (I/O CRN 4) that closely resembles the autocatalytic integral controller reported in (*16*, *72*), yet departs from the standard control-theoretic separation between sensing and actuation. Here, a single molecular conversion reaction performs both operations simultaneously. The cartoon genetic implementation (bottom right) illustrates one possible realization via protease-mediated degradation.

#### Single-process-species yield diverse RPA solutions

In Fig. 4A, the template contains one process species **$X_{1}$** (regulated output) and two controller species **$Z_{1},Z_{2}$**. Allowing up to five appended reactions, GenAI-Net generates a large solution set: The top 100 topologically unique I/O CRNs (gray) robustly converge to the correct setpoints (dashed lines) across scanned (*u*_1,_ *u*_2_) conditions, with three representative networks (I/O CRNs 1, 19, and 28) highlighting distinct topologies that realize the same macroscopic behavior. The diversity graph and reactant-product incidence map summarize structural relationships among solutions and recurrent reaction usage in low-loss designs. Notably, the frequently selected sequestration reaction $Z_{1}+Z_{2}\to \emptyset$ effectively rediscovers the antithetic integral feedback (AIF) motif (*15*). At the same time, GenAI-Net repeatedly selects alternative sensing and actuation mechanisms that improve transients, including a conversion-based sensing reaction **$X_{1}\to Z_{2}$** (rather than the more common catalytic sensing of $X_{1}$) and modified “sequestration” variants in which $Z_{1}$ and $Z_{2}$ jointly contribute to actuation of **$X_{1}$** instead of purely annihilating. Together, these results illustrate how GenAI-Net can simultaneously satisfy stringent steady-state objectives (RPA) while discovering reaction-level modifications that yield favorable dynamics.

#### A minimal single-controller starter I/O CRN reveals compact motifs

Figure 4B reduces the starter I/O CRN to a single controller species Z, yielding a smaller but still nontrivial solution set (nine topologically unique I/O CRNs). Despite this minimal starting point, GenAI-Net still discovers networks that robustly adapt across setpoints and disturbances, with representative solutions shown (I/O CRNs 2, 4, and 9). Notably, one topology (I/O CRN 4) realizes a particularly compact feedback motif: a single molecular conversion reaction simultaneously (i) senses the regulated output and (ii) drives the compensatory action, collapsing sensing and actuation into one reaction channel. This motif is closely related in function to autocatalytic integral control (*16*, *72*), but differs in its nonmodular implementation: rather than a “sense-compute-actuate” separation, the feedback computation is inseparable from the actuation biochemistry itself. The schematic in Fig. 4B (right) illustrates this combined conversion-based mechanism, together with one possible protease-mediated genetic realization in which a protease and its inhibitor form a complex whose conversion back to active protease implements integral-like accumulation while maintaining robustness to persistent disturbances.

#### Scaling to higher-dimensional regulation

Last, fig. S1 demonstrates that GenAI-Net scales to a multispecies setting with two process species $X_{1},X_{2}$ (with **$X_{2}$** as the regulated output) and four controller species $\left(Z_{1},\ldots ,Z_{4}\right)$, where multiple disturbances modulate degradation of each process species. Starting from this higher-dimensional starter I/O CRN and allowing eight additional appended reactions, GenAI-Net again generates a large set of topologically distinct solutions (top 100 unique I/O CRNs) that achieve robust setpoint tracking for the regulated output across the scanned disturbance conditions, with representative topologies shown alongside the aggregate diversity summaries.

Together, these RPA examples show how GenAI-Net can (i) generate families of mechanistically distinct adaptive and high-performance controllers from a common starter I/O CRN, (ii) recover compact control motifs from minimal starting points, and (iii) scale the same design principles to higher-dimensional regulation problems. We also successfully tested other adaptation tasks, namely, habituation and sensitization (*85*, *86*), where inputs are allowed to be time varying. The results are reported in Materials and Methods and fig. S5.

### GenAI-Net generates RPA networks with reduced coefficient of variation in the stochastic reaction kinetics

Intracellular reaction systems can exhibit strongly stochastic dynamics when molecular copy numbers are low (*87*), in contrast to the deterministic regimes considered in our previous examples. Such intrinsic noise can be fundamentally difficult to suppress (*88*). To evaluate GenAI-Net under these conditions, we tasked the method with constructing RPA networks with tunable means and reduced coefficients of variation (CVs) over time, a property often desirable experimentally as it mitigates dynamical intrinsic noise that manifests as cell-to-cell variability. Similar to the RPA task in the deterministic setting, we selected a starter I/O CRN with one output species, three controller species, and two starting reactions regulated by two inputs representing the setpoint and disturbance, respectively (Fig. 5, top left). Allowing up to six appended reactions, GenAI-Net discovers more than 40 topologically distinct controllers that achieve stochastic RPA: The mean trajectory tracks the setpoint across disturbance levels *u*_2_ (Fig. 5, top middle). At the same time, the corresponding CVs remain consistently below the Poisson limit $1/\sqrt{\text{mean}}$, i.e., below the open-loop noise level for **$X_{1}$** in the absence of controller species. This demonstrates that GenAI-Net can jointly satisfy stringent steady-state regulation (RPA), maintain favorable transient performance, and attenuate intrinsic noise under disturbances.

**Fig. 5.**
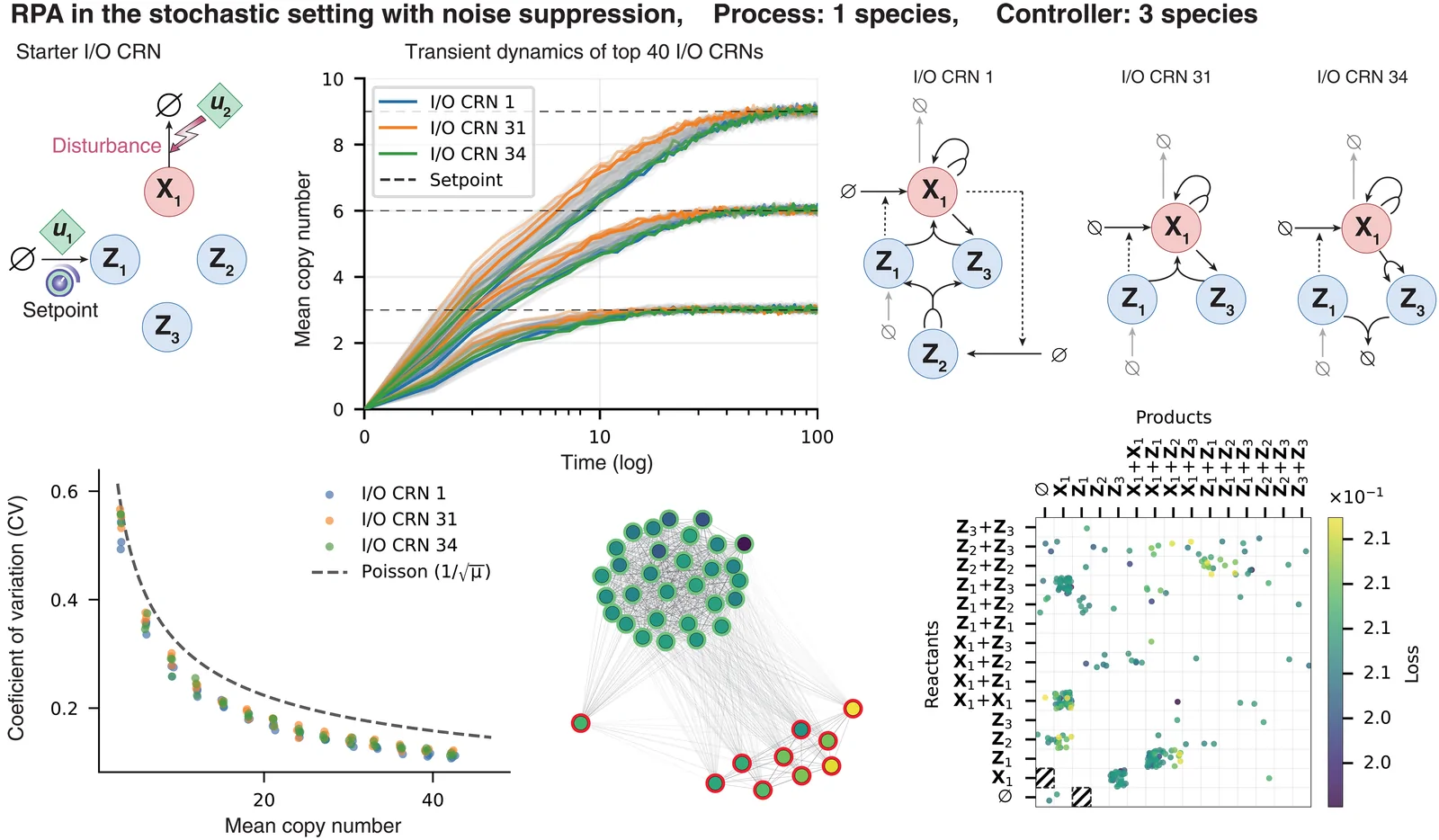
Stochastic RPA with noise suppression: Setpoint tracking with disturbance rejection while minimizing CV. GenAI-Net starts from a stochastic starter I/O CRN (top left) consisting of an output species **$X_{1}$** (red) and three controller species $Z_{1}\;\text{to}\;Z_{3}$ (blue). The input $u_{1}$ specifies the desired mean setpoint for **$X_{1}$**, while $u_{2}$ acts as a disturbance that must be rejected, i.e., changes in $u_{2}$ should not alter the steady-state mean of **$X_{1}$**. From this template, GenAI-Net generates candidate I/O CRNs by appending additional reactions and optimizing a stochastic objective that enforces RPA in the mean—setpoint tracking with rejection of $u_{2}$—while simultaneously minimizing output noise, quantified via the CV of **$X_{1}$** (see Materials and Methods). The time-course panel (top center) shows mean trajectories of **$X_{1}$** versus log time for the generated solution set (thin gray trajectories), with three representative solutions highlighted (colored curves; I/O CRNs 1, 31, and 34) and target setpoint levels indicated by horizontal dashed lines. Across scanned conditions, highlighted solutions robustly converge to the prescribed mean setpoints while maintaining adaptation despite the disturbance $u_{2}$. Noise performance is summarized in the CV panel (second row, left): For the three representative networks, the CV remains below the Poissonian reference level (black curve), indicating sub-Poisson fluctuations and improved noise suppression relative to the open-loop configuration where no controller species are present.

The resulting high-performing networks exhibit recurring motifs reminiscent of AIF (*15*), yet go beyond it in a direction that explicitly targets variance reduction. Whereas classical AIF is known to increase noise relative to open loop (*15*), prior work has sought to mitigate this through added circuitry (e.g., molecular PID) (*23*, *89*), modified actuation (*31*, *32*), or auxiliary antithetic controllers that regulate higher moments (*90*). In contrast, GenAI-Net automatically uncovers motifs that simultaneously address all three objectives—RPA, fast dynamics, and noise suppression. As in other tasks, we cluster the top-performing solutions by topological similarity (Fig. 5, bottom middle) and report representative circuits (Fig. 5, top right).

Mechanistically, these circuits repeatedly use conversion-based sensing **$X_{1}\to Z_{2}$** (rather than the more common catalytic sensing) and, in some families, replace pure annihilation $Z_{1}+Z_{2}\to \emptyset \emptyset$ with a coupled comparison-actuation step $Z_{1}+Z_{2}\to X_{1}$, which both removes controller species and directly actuates **$X_{1}$**. In addition, the networks consistently incorporate negative autoregulation on $X_{1}$ (Fig. 5, top right and bottom right), providing proportional negative feedback that suppresses stochastic fluctuations. Other solutions add parallel sensing routes—for example, an intermediate controller species that catalytically senses the output while producing both controller species—yielding alternative implementations that preserve adaptation while improving transient and noise properties.

### GenAI-Net generates circuits for fate decision

GenAI-Net can also generate biochemical circuits that implement initial condition–dependent fate decisions, a key mechanism in cell differentiation and tissue development (*91*). Substantial effort has been devoted to the theoretical design and experimental implementation of biomolecular circuits that perform decision making through a range of dynamical mechanisms, including multistable behavior (*92*–*97*). To illustrate this capability, we tasked GenAI-Net with designing a system of two species and a starter I/O CRN comprising two reactions (Fig. 6, top left). The resulting network is required to converge to one of two fixed points depending on the initial conditions: If the first species initially dominates, the system converges to (1,0); otherwise, it converges to (0,1). Using this setup and a loss function (see Materials and Methods) that evaluates performance from several selected initial conditions (Fig. 6, middle left), GenAI-Net can generate more than 40 topologically unique, high-performing networks whose dynamics satisfy the above requirement (Fig. 6). In addition, these networks respond rapidly: For most initial conditions, trajectories converge to the vicinity of the desired fixed points with less than 10 time units. The associated topology map and the reactant-product incidence map are given in fig. S2A).

**Fig. 6.**
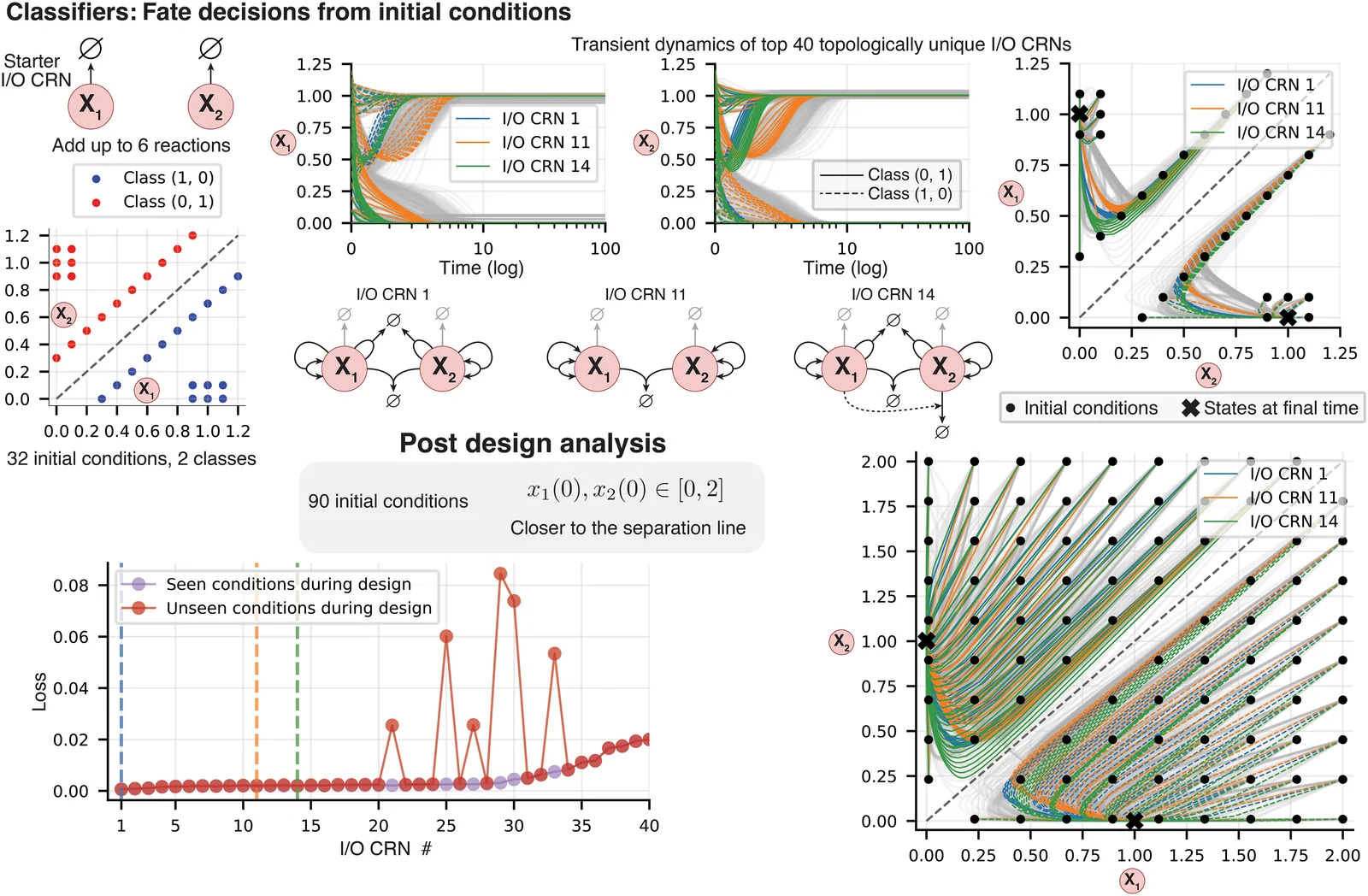
Molecular classifiers from initial condition–dependent fate decisions. GenAI-Net is tasked with generating I/O CRNs that classify the initial state of a two-species system into one of two discrete outcomes. The starter I/O CRN contains two output species, **$X_{1}$** and $X_{2}$, and no external inputs, so classification is driven solely by the network’s dynamics. A set of 32 initial conditions in the $X_{1}\;\text{to}\;X_{2}$ plane (left) is partitioned into two classes [blue: class (1,0); red: class (0,1)] corresponding to desired long-time fates in which one species is high while the other is low (diagonal decision boundary shown). GenAI-Net generates candidate I/O CRNs by appending up to six doubly bimolecular reactions with mass-action kinetics, and optimizing a task loss that penalizes incorrect assignment of initial conditions to the target fate basins (see Materials and Methods). The time-course plots show **$X_{1}$** and **$X_{2}$** trajectories versus log time for the top 40 topologically unique generated I/O CRNs (thin gray curves), with three representative solutions highlighted (colored; I/O CRNs 1, 11, and 14). For each highlighted I/O CRN, trajectories are shown across the full set of labeled initial conditions, demonstrating separation into two attractors consistent with the target classes (solid versus dashed curves). The phase-plane visualization (top right) summarizes the same simulations in the **$X_{1}\;\text{to}\;X_{2}$** plane, where points indicate trajectories from the labeled initial conditions and the two terminal regions correspond to the two classes (crosses). Postdesign analysis (bottom): To test generalization beyond the initial conditions seen during the design process, the generated I/O CRNs are evaluated on an expanded set of 90 initial conditions, some of which are near the decision boundary. The loss-versus-network index plot contrasts initial conditions seen during design (purple) with unseen initial conditions (red), and the phase-plane plot shows the resulting terminal states, highlighting how the learned basin structure extrapolates to unseen initial conditions.

The generated networks consistently favor certain reactions and exhibit specific patterns, as indicated by the reactant-product incidence map and representative circuits in Fig. 6, demonstrating underlying design principles for this task. As in the previous tasks, the representative networks are chosen from across the clusters of high-performing candidates, where clusters are defined by topological similarity (see Fig. 6, bottom left). These representative networks can be viewed as two independent branches—one for each species—combined via a sequestration reaction $X_{1}+X_{2}\to \emptyset \emptyset$. This reaction has a relatively high rate constant and acts as a competing module, driving the nondominant species to approximately zero at steady state and thereby enabling the classification functionality. Moreover, in each branch, an autoregulatory mechanism is provided to regulate the steady-state value of its associated species when it is dominant.

After generation, we perform a postdesign generalization analysis for the classifier circuits. Specifically, in addition to the 32 labeled initial conditions used in the design objective, we simulate each 40 top-ranked I/O CRN on an expanded set of 90 initial conditions, some of which are closer to the target decision boundary (Fig. 6, bottom). We then recompute the classification loss on this expanded set and compare it to the objective loss, providing a direct check that the learned basin geometry extends beyond the discrete initial conditions used during the design process. This postprocessing step does not affect the design loop; it is used only to assess how well the generated fate-decision mechanisms interpolate to nearby initial states.

### Logic circuits

Logic circuits provide a compact and compositional language for programming biochemical systems: By mapping molecular inputs to discrete decisions, they enable modular information processing and serve as reusable building blocks for constructing more complex computations (e.g., multilayer decision cascades, context-dependent responses, and higher-order CRN “programs” that orchestrate dynamics across many species) (*38*, *39*, *42*).

With a starter I/O CRN in which four external inputs $u_{1},\ldots ,u_{4}$ take Boolean values (low/high; 0/1) and modulate the corresponding input species $X_{1},\ldots ,X_{4}$ to regulate an output species **$X_{5}$**, GenAI-Net discovers molecular circuits that implement the irreducible four-input Boolean function $\left(u_{1}\wedge u_{2}\right)\vee \left(u_{2}\wedge u_{3}\right)\vee \left(u_{3}\wedge u_{4}\right)$ (Fig. 7). Allowing six appended doubly bimolecular mass-action reactions yields 29 top-performing I/O CRNs whose output trajectories cleanly separate into low and high digital levels. We highlight the transient dynamics of three representative solutions (I/O CRNs 1, 2, and 4; colored curves) and mark the low/high setpoints with horizontal dashed lines (Fig. 7). An ablation analysis on this task further demonstrated the importance of the various training components. The complete GenAI-Net ultimately achieved lower batch-best and top-*K* losses than variants with reduced or removed risk seeking, self-imitation learning (SIL), or entropy regularization, which frequently converged prematurely to higher-loss solutions (see fig. S8). Furthermore, we assessed the impact of the interplay between the entropy weights of the structure and parameter heads in the policy network. Figure S9 demonstrates that the ratio between these two components acts as a tuning knob that boosts the generative power in terms of topological diversity of the I/O CRNs.

**Fig. 7.**
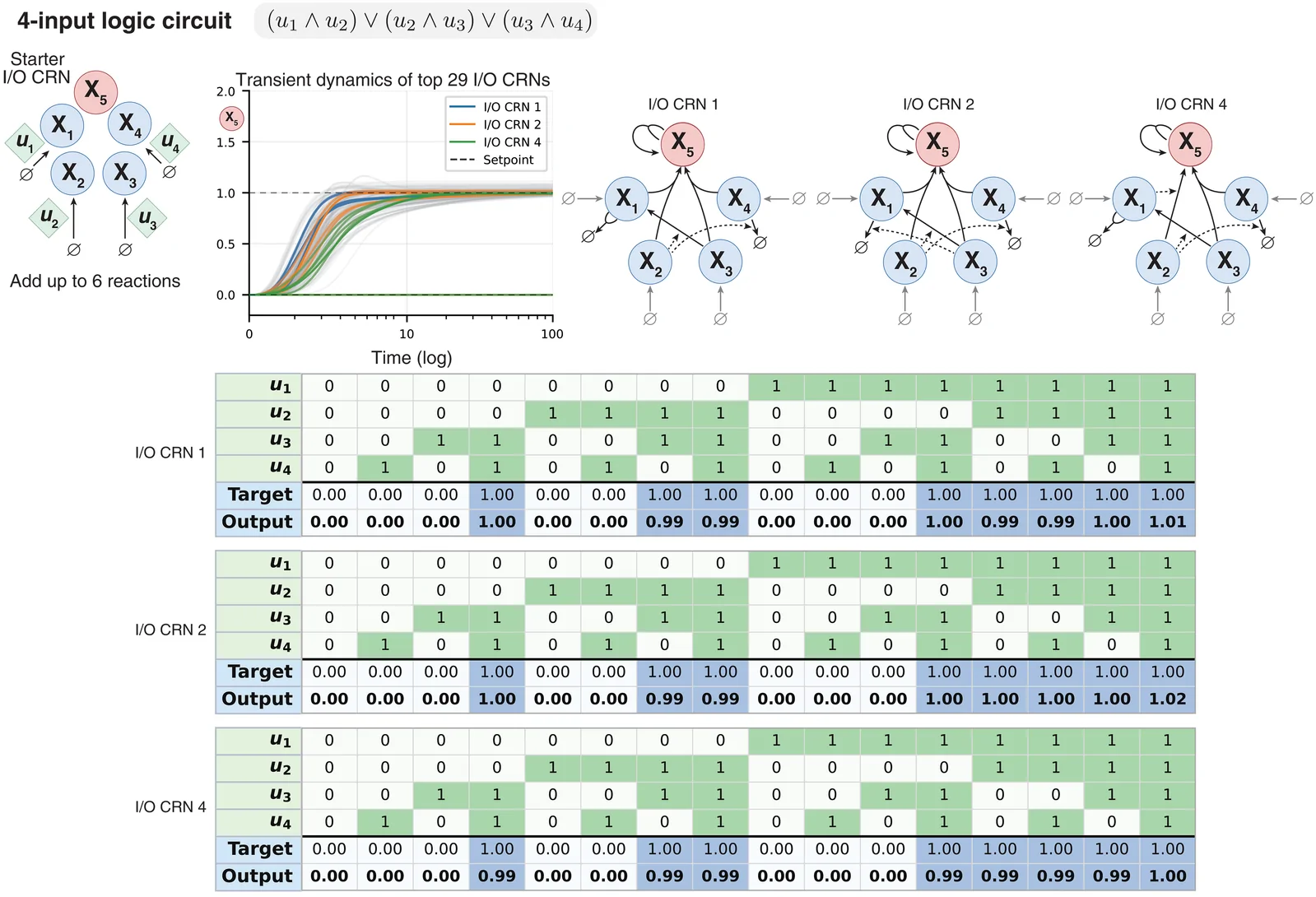
Four-input irreducible logic circuit discovery. GenAI-Net begins with a starter I/O CRN (top left) in which four external inputs $u_{1}-u_{4}$ act on corresponding input species **$X_{1}\;\text{to}\;X_{4}$** (blue) to regulate an output species (red). From this starter I/O CRN, GenAI-Net generates candidate I/O CRNs by appending additional reactions and optimizing a loss (see Materials and Methods) that rewards correct realization of the target four-input irreducible Boolean function $\left(u_{1}\wedge u_{2}\right)\vee \left(u_{2}\wedge u_{3}\right)\vee \left(u_{3}\wedge u_{4}\right)$. The time-course panel shows the output response versus time for the discovered solution set (thin gray trajectories), with three representative solutions highlighted (colored curves) and the low/high digital setpoints indicated by horizontal dashed lines. Example I/O CRN topologies for the highlighted networks are shown to the right, together with their truth tables (bottom), demonstrating high-precision binarization of the output across all 16 input combinations.

These solutions share two recurring design features. First, the output is stabilized by an autocatalytic degradation motif, which suppresses overshoot and accelerates convergence. Second, the logic is computed in an analog manner through direct or catalyzed conversion channels in which pairs of input species promote production of **$X_{5}$**, encoding the conjunctive clauses of the target function (Fig. 7, example topologies). Tight binarization across all 16 input combinations is achieved by adding catalytic degradation and interconversion reactions among $X_{1},\ldots ,X_{4}$ that tune the effective high-state setpoint, so true cases saturate near unity rather than increasing with input magnitude (Fig. 7), truth tables for I/O CRNs 1, 2, and 4). The associated topology map and the reactant-product incidence map are given in fig. S2B).

Restricting the search to at most five appended reactions yields 40 top-ranked candidates, and we show representative topologies (I/O CRNs 1, 6, and 26; fig. S2C). With this reduced reaction budget, the setpoint-conditioning mechanisms above are typically absent, leading to a systematic overshoot above the nominal high level in the all-inputs-high condition (fig. S2C, time courses). This overshoot does not affect threshold-based classification, but it reveals a trade-off between circuit compactness and tight digital saturation that is alleviated by the sixth reaction in Fig. 7. Last, to demonstrate GenAI-Net’s ability to operate on reaction libraries with multiparameter kinetics, we apply it to a Hill-type production library and successfully generate diverse I/O CRNs, as shown in fig. S3.

### GenAI-Net provides biochemical oscillators with specific centers and tunable frequencies

Biological rhythms are widespread in biology, precisely regulating life processes in response to oscillatory environmental cues. Despite the discovery of natural biochemical oscillators (*98*–*102*), and the development of synthetic ones (*6*, *103*–*105*), designing circuits with specified or tunable oscillatory parameters (frequency, center, amplitude, etc.) remains prohibitively challenging, with only a few rational designs available (*106*, *107*). We therefore tasked GenAI-Net with constructing such biochemical oscillators from a starter I/O CRN comprising three species and four reactions where all three species are diluted (top-left panels in Figs. 8 and 9).

**Fig. 8.**
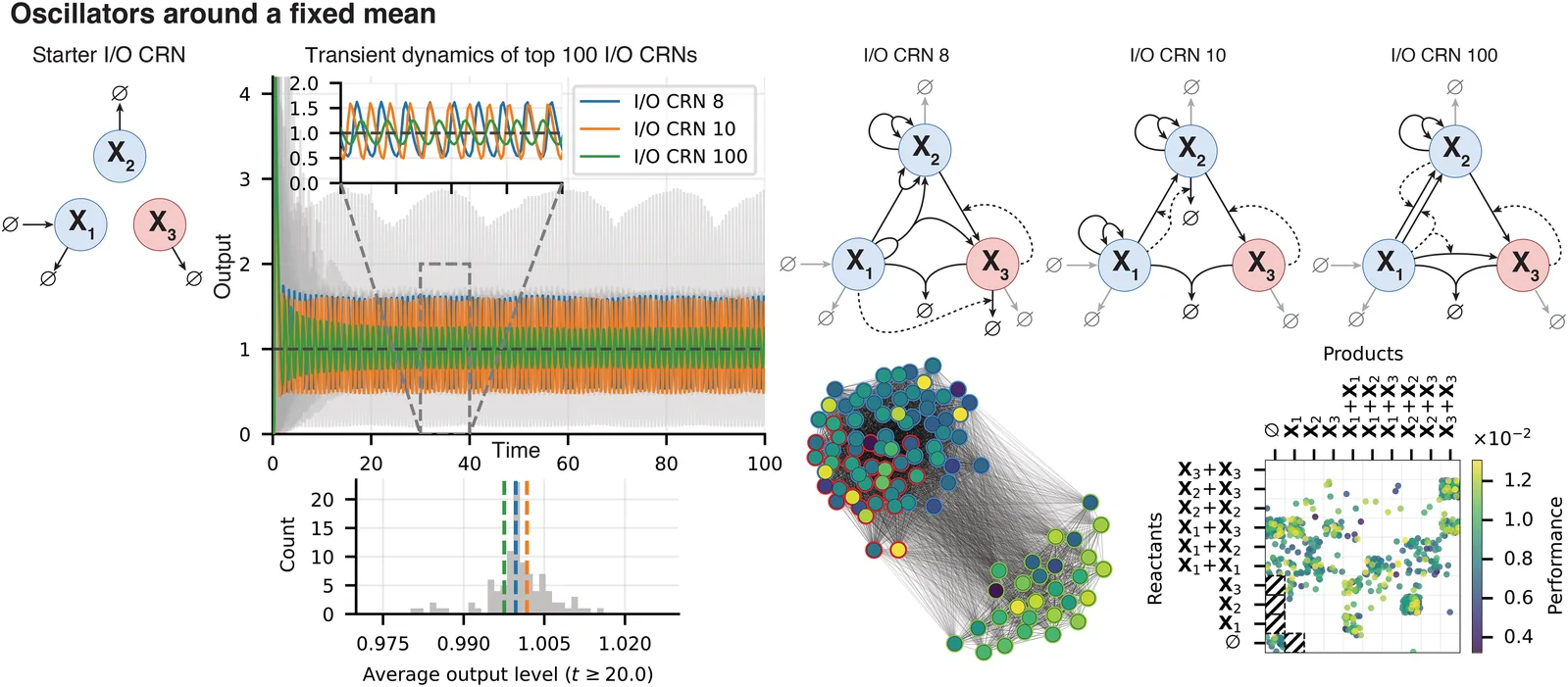
Molecular oscillators. Oscillators around a fixed mean. GenAI-Net begins with a starter I/O CRN (left) with three species, $X_{1},X_{2}$ (blue), and output $X_{3}$ (red), and no external input. From this template, GenAI-Net generates candidate I/O CRNs by appending up to six doubly bimolecular reactions, following mass-action kinetics, and optimizing a task objective that rewards sustained oscillations while constraining the temporal average output to a fixed mean equal to one (see loss function in Materials and Methods for more details). The center panel shows output trajectories for the top 100 topologically unique generated I/O CRNs (thin gray curves), with three representative solutions highlighted (colored; I/O CRNs 8, 10, and 100). The histogram (bottom left) summarizes the distribution of time-averaged output levels across the 100 solutions (computed over *t* ≥ 20), illustrating concentration near the target mean. Example I/O CRN topologies for the highlighted networks are shown to the right. The graph visualization (bottom center) summarizes topological diversity across the solution set, and the reactant-product incidence map (bottom right) summarizes reaction usage across generated solutions.

**Fig. 9.**
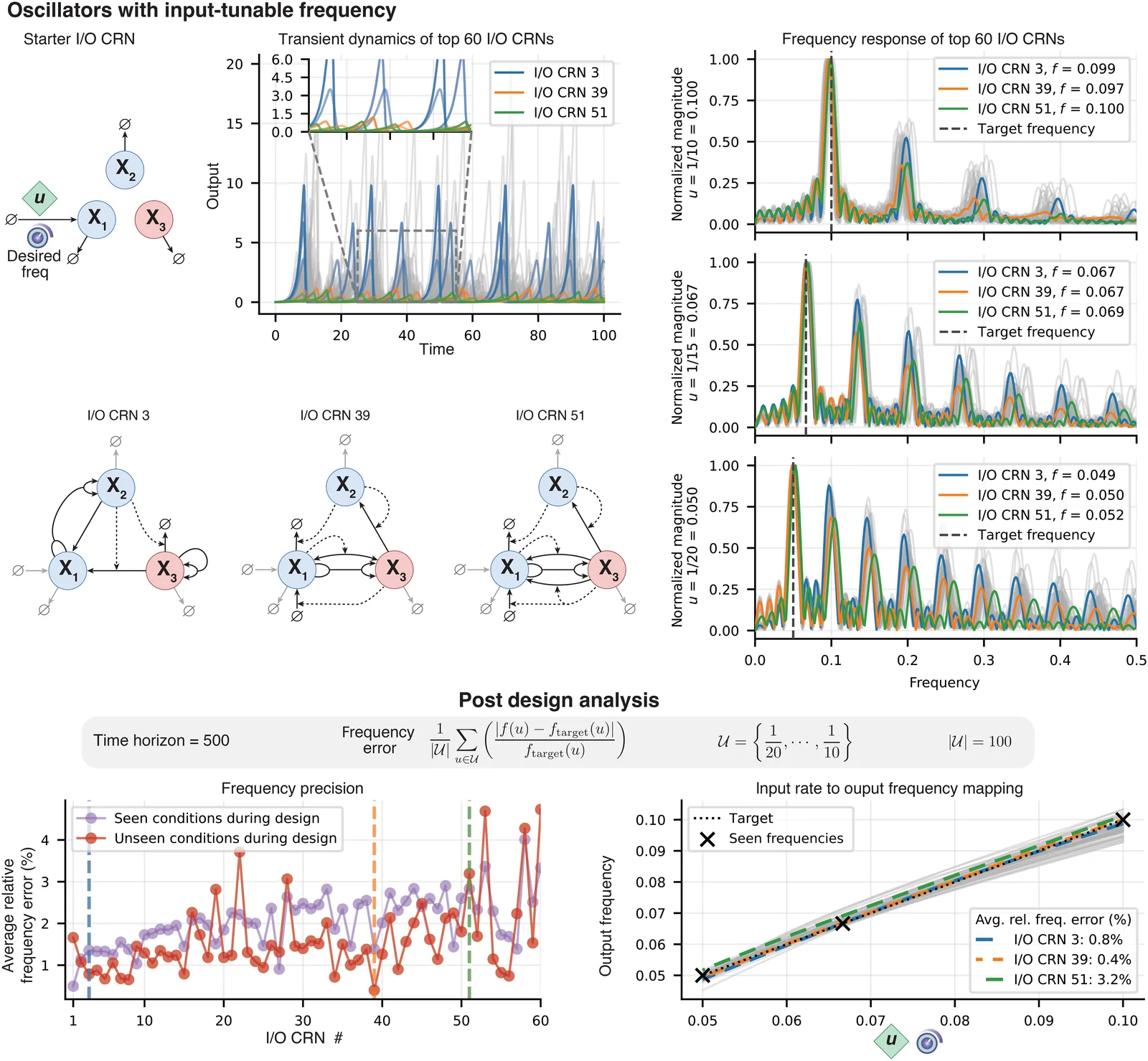
Oscillators with input-tunable frequency. A related starter I/O CRN introduces an external input *u*_1_ (green) that specifies a target oscillation frequency. GenAI-Net again appends up to six reactions and optimizes a frequency-tracking objective (see loss function in Materials and Methods for details), producing I/O CRNs whose oscillation frequency is tuned by *u*_1_ while maintaining oscillatory behavior. The left-center plot shows time-domain output trajectories for the top 60 topologically unique solutions (gray), with three representative networks highlighted (I/O CRNs 1, 2, and 51). Frequency-domain behavior is summarized on the right: For three example input settings, the normalized magnitude spectra of the highlighted networks are shown, with dashed lines indicating the target frequencies; legend annotations report the achieved dominant frequencies *f* for each I/O CRN. Example network topologies for the highlighted solutions are shown beneath the time-domain plot, and the topological diversity graph and reactant-product incidence map (bottom) summarize structural diversity and reaction usage across the generated oscillator solution set. Postdesign testing beyond seen conditions: To assess generalization, the top I/O CRNs are evaluated on a dense set of 100 input frequencies spanning the design range (schematic, top), using a longer simulation horizon (*t*_f_ = 500). The left panel compares the average relative frequency error on the input frequencies used during design (purple) versus additional frequencies not used during design (red) across the top 60 I/O CRNs. Using the longer horizon improves frequency estimation and yields higher precision, including on unseen input frequencies. The right panel plots output frequency versus input frequency for all top 60 I/O CRNs (gray) and the representative networks (colored), showing close agreement with the identity line and reporting the average relative error for each highlighted I/O CRN.

We begin with oscillators whose trajectories are required to oscillate around a prescribed center (Fig. 8). In this setting the starter I/O CRN has no inputs, and the objective targets the mean (center) of the output oscillation. GenAI-Net discovers more than 100 topologically distinct high-performing networks, with oscillator centers tightly clustered around the target value (Fig. 8, histogram). Three representative solutions are highlighted with colored trajectories alongside their corresponding topologies, while the topological diversity graph summarizes the breadth of distinct solutions found by GenAI-Net. The sequestration reaction **$X_{1}+X_{3}\to \emptyset \emptyset$** emerges repeatedly in the generated CRNs (see the table in Fig. 8), suggesting that sequestration constitutes a structurally favored mechanism for oscillatory regulation. Notably, related sequestration dynamics play a central role in the mammalian circadian clock (*108*, *109*). We are grateful to J. K. Kim for drawing our attention to this connection.

We next consider oscillators with input-tunable frequency (Fig. 9). Here, the starter I/O CRN includes an input *u*_1_ that modulates the production rate of **$X_{1}$**, providing a tuning knob for the oscillation frequency of the output species **$X_{3}$**. We evaluate three target periods (*T* = 10, 15, 20) and so the input *u*_1_ takes three values *u*_1_ ϵ {1/20,1/15,1/10} and find that GenAI-Net again produces more than 60 high-performance networks (Fig. 9). Fourier spectra (Fig. 9, right) confirm that, for each input level, the fundamental frequency aligns with the prescribed target period. As in the previous task, the diversity graph and reactant-product incidence map reveal substantial topological variation and recurring reaction usage among the generated solutions (see fig. S4), highlighting the framework’s ability to generate multiple distinct implementations of the same dynamical specification.

After generation, we perform a postdesign generalization test for the frequency-tunable oscillators. Specifically, although the design objective in Fig. 9 evaluates only a sparse set of target frequencies, we take the top I/O CRNs and simulate them on a dense sweep of 100 input frequencies spanning the same range. To improve frequency estimation, we use a longer simulation horizon (*t*_f_ = 500), which yields higher-precision frequency measurements and correspondingly lower relative error, including for input frequencies not encountered during design. The resulting input-output frequency curves closely track the identity relation, indicating that many generated oscillators interpolate smoothly across the design range rather than merely matching the discrete targets.

### Benchmarking against existing CRN design methods

To benchmark GenAI-Net against other search techniques, we adapted existing approaches to our tasks to handle CRN dynamics with inputs. In particular, we compared our RL approach with ReactionNetworkEvolution.jl (RNE) (*54*), CircuiTree (*56*), and random search on the RPA and logic-circuit tasks under matched budgets of full task simulations (fig. S11). GenAI-Net consistently reduced the best-so-far loss more rapidly and reached lower median losses than the existing methods. To improve statistical evidence, we further compared GenAI-Net with RNE. By controlling for network size, we have shown that standard RNE often uses substantially larger networks, whereas GenAI-Net attains strong performance with the prescribed reaction budget (fig. S12). Last, following the exact task reported in (*54*), where the RNE setting targets a fixed-frequency and fixed-amplitude oscillator (i.e CRNs with no inputs), GenAI-Net achieved higher success rates and recovered diverse valid oscillator topologies (see fig. S10).

### Modularity-induced increase of solution density

Searches over CRN topologies may be viewed as “needle-in-a-haystack” problems: As the allowed network size grows, the number of possible reaction sets grows rapidly, and valid designs may appear increasingly diluted. Modularity can break this intuition. When the desired behavior is dictated by a structural submodule, larger CRNs can contain the same functional core as a subnetwork in many distinct ways, provided the added reactions do not disrupt its function. In this section, we theoretically analyze the discrete search space in terms of reaction topologies while disregarding the continuous parameter space.

#### Density of valid CRN solutions

Let Ω be a reaction library that encodes for the topologies of all possible reactions with ∣Ω∣≜M. Suppose the target behavior is triggered by a fixed core submodule $M_{0}\subseteq \Omega$, with $|M_{0}|≜m_{0}$. We consider CRNs

$$M=M_{0}\cup \tilde{M},\;\;\;|\tilde{M}|≜j,\;\;\;|M|=m_{0}+j$$

where $\tilde{M}\subseteq \Omega \backslash M_{0}$ denotes the reactions that are added to the CRN on top of the core submodule $M_{0}$. Let $\Omega_{\text{safe}}\subseteq \Omega \backslash M_{0}$ denote the safe reactions that can be added in any combination to the CRN without disrupting the core function, with $K_{\text{safe}}≜|\Omega_{\text{safe}}|\;$. For fixed *j*, the total number of candidate CRNs is

$${\text{CRN}}_{\text{tot}}\left(j\right)=\left(\begin{array}{c} M\\ m_{0}+j \end{array}\right)$$ (2)

while the number of valid CRNs is

$${\text{CRN}}_{\text{sol}}\left(j\right)=\left(\begin{array}{c} K_{\text{safe}}\\ j \end{array}\right)$$ (3)

In general, it is possible that adding one unsafe reaction, which destroys the functionality, can be reversed by adding another reaction to recover the lost functionality. Such cases are not considered in this analysis to treat the worst-case scenario. Hence, the density of valid CRN solutions in the search space is given by

$$\rho \left(j\right)≜\frac{{\text{CRN}}_{\text{sol}}\left(j\right)}{{\text{CRN}}_{\text{tot}}\left(j\right)}=\frac{\left(\begin{array}{c} K_{\text{safe}}\\ j \end{array}\right)}{\left(\begin{array}{c} M\\ m_{0}+j \end{array}\right)}$$ (4)

Equivalently, ρ(j) can be decomposed as

$$\rho \left(j\right)=\frac{1}{{\text{CRN}}_{\text{tot}}\left(0\right)}\underset{p_{\text{safe}}^{\left(j\right)}}{\underbrace{\frac{\left(\begin{array}{c} K_{\text{safe}}\\ j \end{array}\right)}{\left(\begin{array}{c} M-m_{0}\\ j \end{array}\right)}}}\underset{\text{coverage}}{\underbrace{\left(\begin{array}{c} m_{0}+j\\ m_{0} \end{array}\right)}}$$ (5)

Here, $p_{\text{safe}}^{\left(j\right)}$ is the probability that *j* reactions drawn from the noncore library are all safe, while $\left(\begin{array}{c} m_{0}+j\\ m_{0} \end{array}\right)$ counts the possible *m*_0_-reaction subsets inside a CRN of size *m*_0_ + *j*. We consider two separate cases: increasing the number of added reactions *j* and the library size *M*.

#### Enlarging the CRN size

Fix *M* and $K_{\text{safe}}$, and compare the number of added reactions *j* and *j* + 1

$$\frac{\rho \left(j+1\right)}{\rho \left(j\right)}=\frac{\left(K_{\text{safe}}-j\right)\left(m_{0}+j+1\right)}{\left(j+1\right)\left(M-m_{0}-j\right)}$$ (6)

Thus ρ(j+1)>ρ(j) if and only if

$$\frac{K_{\text{safe}}-j}{M-m_{0}-j}>\frac{j+1}{m_{0}+j+1}$$ (7)

The condition states that increasing the CRN size by one raises the solution density when the fraction of remaining admissible reactions that are safe exceeds the relative burden of adding one more reaction to the enlarged network.

Next, we examine how enlarging the library changes the stringency of this condition. The right-hand side of the inequality is unchanged when the library is enlarged, so it suffices to track the left-hand side. If an unsafe reaction is added to the library, then M↦M+1 and $K_{\text{safe}}\mapsto K_{\text{safe}}$. Thus, an unsafe library addition makes the enlargement condition more stringent. However, if a safe reaction is added to the library, then M↦M+1 and $K_{\text{safe}}\mapsto K_{\text{safe}}+1$. Observe that

$$\frac{K_{\text{safe}}+1-j}{M+1-m_{0}-j}>\frac{K_{\text{safe}}-j}{M-m_{0}-j}$$ (8)

whenever $K_{\text{safe}}<M-m_{0}$. Thus, a safe library addition makes the enlargement condition less stringent, except in the degenerate case where all noncore reactions are already safe.

#### Enlarging the reaction library

Now, fix the candidate CRN size $m_{0}+j$ and increase the library size from *M* to *M* + 1. If the added reaction is unsafe, then M↦M+1 and $K_{\text{safe}}\mapsto K_{\text{safe}}$. We have

$$\frac{\rho \left(M+1,K_{\text{safe}}\right)}{\rho \left(M,K_{\text{safe}}\right)}=\frac{\left(\begin{array}{c} M\\ m_{0}+j \end{array}\right)}{\left(\begin{array}{c} M+1\\ m_{0}+j \end{array}\right)}=\frac{M+1-m_{0}-j}{M+1}<1$$ (9)

Thus, adding an unsafe reaction always decreases the density. If the added reaction is safe, then M↦M+1 and $K_{\text{safe}}\mapsto K_{\text{safe}}+1$. We have

$$\frac{\rho \left(M+1,K_{\text{safe}}+1\right)}{\rho \left(M,K_{\text{safe}}\right)}=\frac{K_{\text{safe}}+1}{K_{\text{safe}}+1-j}\frac{M+1-m_{0}-j}{M+1}$$ (10)

Hence, a safe addition increases density if and only if

$$\frac{K_{\text{safe}}+1}{M+1}<\frac{j}{m_{0}+j}$$ (11)

This condition states that the fraction of safe reactions in the augmented library must be lower than the fraction of added reactions in the CRN. Intuitively, when this condition is violated, the benefits of augmenting the library with a safe reaction is outweighed by the reduction of the probability of hitting the core submodule. Furthermore, observe that the right-hand side of the inequality is an increasing function of *j*. Thus, the condition becomes less stringent as *j* increases. Therefore, for larger CRNs, a newly added safe reaction to the library is more likely to increase density.

Together, these analyses show how modularity can induce regimes in which enlarging the search space increases, rather than decreases, the density of valid CRN solutions. As a demonstration, we have carried out a computational analysis for the solution density of the maxRPA task as defined in section 1.4 of (*81*). The results reported in fig. S13 align with our theoretical analysis: Increasing the search space by allowing more species and/or reactions can, in certain cases, increase the density of solutions and thus make the search problem easier to solve. We close this section by highlighting that the value of *K*_safe_ is application-dependent: It depends on the target behavior, reaction library, kinetics, and definition of disruption.

## DISCUSSION

We presented GenAI-Net, a generative AI framework for the automated design of CRNs from high-level dynamical specifications. GenAI-Net exploits the asymmetry between the ease of evaluating a proposed CRN by simulation and the difficulty of discovering one that satisfies a target behavior. By placing an AI agent in the loop, GenAI-Net iteratively proposes reactions (including kinetic parameters and, when relevant, input influences), evaluates completed networks with a task-defined loss, and refines its proposal distribution to generate diverse, high-performing solutions.

Across a broad set of benchmarks—including dose-response shaping (Hill-type, Michaelis-Menten, ultrasensitive, and nonmonotonic profiles), logic circuits implementing complex Boolean functions, classification by initial conditions, oscillators with constrained mean or input-tunable frequency, RPA under deterministic and stochastic dynamics (including simultaneous noise reduction), and habituation/sensitization—GenAI-Net consistently produced families of topologically distinct networks that match the desired behaviors. Beyond performance, the resulting solution sets enabled mechanistic insight: Recurring reaction patterns and cluster-level structure revealed alternative motifs for achieving the same specification.

Robustness to parameters can be incorporated directly into the GenAI-Net design objective by evaluating candidate networks under perturbed operating conditions and penalizing designs that fail to preserve the desired behavior. We used this strategy when designing robust-perfect-adaptation circuits, where disturbances were explicitly introduced during evaluation and the loss required the output to maintain its target setpoint despite these disturbances. This design of the loss function forced GenAI-Net to discover molecular integral controllers that deliver RPA despite these parametric disturbances. Robustness also emerged, in certain cases, even though it was not explicitly requested. Postdesign perturbation analyses showed that several initial-condition classifiers and oscillators with input-tunable frequency retained their functionality under simultaneous random perturbations of their kinetic parameters, despite parameter robustness not being included in their respective loss functions (figs. S6 and S7). One possible explanation is the entropy regularization used during training, which encourages exploration over both reaction structures and continuous kinetic parameters and may consequently favor solutions occupying broader regions of high performance in parameter space rather than narrowly tuned optima. Although establishing this mechanism will require further investigation, these results suggest that GenAI-Net can support both explicitly optimized robustness and robustness that emerges as a by-product of diversity-promoting generative search.

Despite all these successes, several directions remain to be explored in future work to further improve the method and extend its applicability to more advanced biological studies. From a methodological perspective, the computational efficiency of our generative approach could potentially be improved by adopting more advanced neural network architectures and generative paradigms, such as transformer-based models (*110*), GFlowNets (*111*), and diffusion models (*112*). In addition, incorporating more advanced training strategies, such as proximal policy optimization (PPO) (*113*), may further accelerate learning and yield more robust performance. Moreover, an adaptive hyperparameter-tuning scheme would help streamline practical use and reduce the burden of manual calibration for end users.

Our current implementation is designed to run on a single computer node (workstation or cluster job); however, because candidate CRNs and evaluation conditions are largely independent, the workflow is inherently parallel. Extending GenAI-Net to distributed, multinode execution is therefore a natural next step, but it would require careful systems engineering (e.g., asynchronous scheduling of simulations, efficient aggregation of rewards/gradients) to correctly handle the distributed training of our agent.

On the other hand, GenAI-Net naturally extends to richer design settings. One immediate direction is to treat input assignment itself as part of the search, allowing the agent to decide not only which reactions to add but also which external signals modulate each propensity. An important direction is to automate the incorporation of experimental implementability directly into the design loop. In the present work, kinetic parameters are treated largely as unconstrained optimization variables, and high-performing designs may therefore occupy parameter regimes that are difficult to realize experimentally. GenAI-Net can address this limitation by incorporating literature-informed parameter priors, experimentally measured bounds, and feasibility penalties into its parameter-sampling distributions and evaluation objectives. We are currently pursuing this direction by integrating experimental implementability criteria directly into the RL loop, allowing candidate designs to be evaluated jointly for dynamical performance and practical realizability. Although we focused here on reaction libraries with mass-action kinetics and Hill kinetics (see fig. S3), GenAI-Net is not limited to these settings. The same framework can operate with arbitrary, user-defined reaction libraries in which each reaction carries multiple kinetic parameters and follows more complex propensity forms. The framework can also leverage more sophisticated simulation environments that incorporate additional biological context—such as growth, dilution, resource limitations, burden, spatial effects, or host-circuit interactions—so that designs are optimized under more realistic operating conditions. Beyond the benchmarks explored here, the same loop could be applied to a wider range of objectives and larger networks, including multimodule behaviors and multiobjective specifications. Last, a complementary avenue is to decouple structure and parameters more explicitly: The agent could focus primarily on proposing reaction topologies, while an embedded optimizer (or inner-loop parameter-fitting routine) searches for the best kinetic parameters for each proposed topology, potentially improving sample efficiency and sharpening the topology-level search.

GenAI-Net is intended as a general design engine rather than a solver for a single task class. Users can specify a starter I/O CRN, a reaction library, kinetic semantics, and an evaluation objective, allowing the same framework to be applied across modeling assumptions and application domains. Looking forward, GenAI-Net opens a path toward more programmable molecular engineering, where circuit discovery is guided directly by behavioral specifications and where interpretable reaction-level motifs can be rapidly surfaced, compared, and translated into experimental implementations.

## MATERIALS AND METHODS

### Components of the RL Loop

We begin by defining the agent and its action space (reaction library) and then proceed to the environment and its state space (I/O CRNs). We then describe the translator that maps CRNs into an agent-interpretable representation, followed by the policy architecture used by the agent.

#### The agent and action space

The agent is responsible for proposing the next reaction to append to the current incomplete I/O CRN, iterating until the I/O CRN is complete. We formalize the agent’s decision as sampling an action from a reaction library, conditioned on the current translated CRN representation. Equivalently, the agent defines a conditional distribution over the next reaction given the current CRN state, i.e., ℙ(next reaction∣CRN), as schematized in Fig. 2A.

Informally, the action space A corresponds to the set of reactions that may appear in a valid CRN. Because different application settings permit different classes of reactions, we treat A as a user- or task-chosen reaction library, which is a core component of our formulation containing all permissible reaction templates. Let reaction templates in the library be indexed by ℓ∈{1,…,M}, where each template ℓ corresponds to a human-interpretable reaction template (stoichiometry together with associated kinetic parameter variables). When external inputs are present, each reaction may additionally be configured with an input-influence pattern indicating which input channels modulate its propensity. Concretely, let there be $n_{u}$ input channels and let G denote the (finite) set of admissible input-influence patterns (e.g., $G\subseteq {\left\{0,1\right\}}^{n_{u}}$, where a binary vector g∈G specifies which inputs act on the reaction). Reaction template ℓ has $p_{\ell }\in {\mathbb{N}}_{0}$ parameters with domains $S_{\ell ,\ell^{\prime }}$ for $\ell^{\prime }=1,\ldots ,p_{\ell }$. An action is then a tuple consisting of (i) a discrete reaction-type index, (ii) its continuous parameter vector, and (iii) an input-influence choice

$$A≜\left\{\left(\ell ,\theta ,g\right)|\ell \in \left\{1,\ldots ,M\right\},\;\theta =\left(\theta_{1},\ldots ,\theta_{p_{\ell }}\right),\theta_{\ell^{\prime }}\in S_{\ell ,\ell^{\prime }}\forall \ell^{\prime },g\in G\right\}$$ (12)

When a task requires no external input assignments, we take *n_u_* = 0 and G={∅}, reducing to the (ℓ,θ) action form. A is a hybrid space with both discrete (reaction template index) and continuous (kinetic parameters) components.

#### The environment and state space

Let $s_{t}$ denote the environment state at construction step *t*, defined as the current (possibly incomplete) I/O CRN obtained by starting from the user-provided starter I/O CRN and appending *t* reactions. Thus, is exactly the starter I/O CRN, and for t>0, the state $s_{t}$ encodes the set of selected reaction templates together with their chosen parameter values and input-influence assignments.

Formally, the state space is the combinatorial set of all such CRNs reachable by appending up to *m* reactions from the library

$$S≜\overset{m-1}{\underset{t=0}{\cup }}\;S_{t},\text{with}\;S_{t}≜\left(\begin{array}{c} A\\ t \end{array}\right)≜\left\{B\subseteq A|\;|B|=t\right\}$$ (13)

We detail the actual size of the search space under mass action kinetics in a subsequent section. In practice, we enforce validity constraints during construction (e.g., preventing duplicate reaction types and considering the starter I/O CRN). We next describe how a state $s_{t}$ is mapped into the agent-interpretable representation on which the policy conditions.

#### The translator

The translator maps a human-readable CRN state $s_{t}$ (a reaction list with parameters and input influences) into a fixed-format, agent-interpretable observation. Concretely, it encodes, for each library reaction template, whether it is present in the current I/O CRN and, if so, its current parameter value(s) and input-influence assignment. This yields a structured representation of constant dimension across all *t*, enabling the policy to operate on incomplete CRNs of varying sizes. A running example is shown in Fig. 2A (left), where a toy I/O CRN is translated into this fixed-format encoding using an example library; the reaction highlighted by the red box illustrates how a single library entry is represented.

#### Policy implementation

For compactness, we describe the policy architecture for the case where input assignment is not required (Fig. 2B). The agent is parameterized by neural-network weights **ϕ** and defines a conditional distribution over the next reaction type and its kinetic parameters given the current translated CRN state *s*, where we drop the subscript *t* for notational convenience. As illustrated in the left panel of Fig. 2B and zoomed-in on the right, the policy is implemented with a shared network trunk that produces an embedding of *s*, followed by two heads, and is written autoregressively as

$$\pi_{\phi }\left(\ell ,\theta |s\right)=\pi_{\phi }\left(\ell |s\right)\pi_{\phi }(\theta |s,\ell )$$ (14)

where we recall that ℓ denotes the reaction template index (or ID) in the library, and θ denotes its parameter values. The structure head outputs logits over reaction templates ℓ∈{1,…,M}; logits for reaction templates already present in the current partial CRN are masked to enforce uniqueness, yielding the categorical distribution $\pi_{\phi }(\ell |s_{t})$. Conditioned on the sampled template ℓ, the parameter head outputs a continuous distribution over **θ** (we use a log normal family), yielding $\pi_{\phi }(\theta |s,\ell )$. During sampling, the agent draws $\ell ∼\pi_{\phi }(\ell |s)$ and then $\theta ∼\pi_{\phi }(\theta |s,\ell )$. The corresponding log-probabilities are summed up in log-space. Furthermore, we compute a weighted entropy regularizer by combining the Shannon entropy of the discrete component and the differential entropy of the continuous component

$$H_{\pi_{\phi }}^{w}\left(s\right)=H\left[\pi_{\phi }\left(\cdot |s\right)\right]+wE_{\ell ∼\pi_{\phi }\left(\cdot |s\right)}\left[H\left[\pi_{\phi }\left(\cdot |s,\ell \right)\right]\right]$$ (15)

where the expectation appears because the parameter distribution $\pi_{\phi }(\theta |s,\ell )$ (and thus its differential entropy) depends on the reaction template ℓ, so we average over ℓ under $\pi_{\phi }(\ell |s)$ to obtain a single entropy value for the joint policy at state **s**. Last, *w* is a training hyperparameter controlling the relative strength of the continuous-entropy term, which we discuss in the subsequent training section.

### The training

Training proceeds via episodic construction rollouts of fixed length *m*. Starting from the initial state $s_{0}$ (the starter I/O CRN), the agent repeatedly samples an action and the environment deterministically applies it through the state transition operator T (by appending the proposed reaction)

$$a_{t}∼\pi_{\phi }(\cdot |s_{t}),\;\;\;s_{t+1}=T\left(s_{t},a_{t}\right),\;\;\;\;t=0,1,\ldots ,m-1$$ (16)

yielding a trajectory $\tau =(s_{0},a_{0},\ldots ,s_{m-1},a_{m-1},s_{m})$, where $s_{m}$ is a complete I/O CRN. The completed CRN $s_{m}$ is then simulated and evaluated using a task-specific objective to yield a scalar loss $L_{\text{task}}(s_{m})$ (defined by the user for each application, as illustrated in our examples). Because partial CRNs can exhibit behavior that is not predictive of the final circuit, we assign reward only at termination and optimize terminal performance.

#### Policy optimization

We define the terminal reward as the negative task loss

$$R\left(\tau \right)≜R\left(s_{m}\right)≜-L_{\text{task}}(s_{m})$$ (17)

Under the standard REINFORCE formulation (*114*) with terminal rewards, the policy is trained by solving the expected-return maximization problem

$$\underset{\phi }{\text{max}}E_{\tau ∼p_{\phi }}\left[R\left(\tau \right)\right]=E_{\tau ∼p_{\phi }}\left[-L_{\text{task}}\left(s_{m}\right)\right]$$ (18)

where $p_{\phi }(\tau )$ denotes the rollout distribution induced by the policy $\pi_{\phi }$ and the deterministic transition T. In GenAI-Net, we build on this objective and introduce four modifications—(i) a risky (top-*K*) objective that emphasizes high-performing rollouts (*115*), (ii) baseline subtraction using the worst reward among the top-*K* samples, (iii) hybrid entropy regularization combining discrete (Shannon) and continuous (differential) entropy terms (*116*, *117*), and (iv) an SIL term based on a hall-of-fame replay buffer (*118*)—each described in detail in what follows.

#### *Risk-sensitive (top-*K*) objective with baseline subtraction*

Standard REINFORCE maximizes the expected return, which in batch form averages rewards across sampled rollouts. To reduce this averaging effect and emphasize rare high-performing solutions, we skew the objective toward the upper tail of the reward distribution. Let $q_{\alpha }$ denote the α-quantile of the terminal reward R(τ) under $\tau ∼p_{\phi }$ {i.e., $\mathbb{P}\left[R\left(\tau \right)\leq q_{\alpha }\right]=\alpha$}. The corresponding upper-tail (top-quantile) objective is

$$J_{\alpha }(\phi )≜E\left[R\left(\tau \right)|R\left(\tau \right)\geq q_{\alpha }\right]$$ (19)

which is closely related to CVaR-style risk-sensitive optimization and the risky policy gradients viewpoint (*115*). In practice, we estimate this objective from a batch of *N* rollouts ${\left\{\tau^{\left(i\right)}\right\}}_{i=1}^{N}$ with terminal rewards $r_{i}≜R\left(\tau^{\left(i\right)}\right)$. Let $r_{\left(1\right)}\geq \cdots \geq r_{\left(N\right)}$ be the sorted rewards and choose K=⌈N(1−p)⌉, so that the empirical threshold $r_{\left(K\right)}$ estimates $q_{\alpha }$. We further apply baseline subtraction with the batch baseline defined as the worst reward among the top-*K* samples

$$B_{\text{top}K}≜r_{\left(K\right)}$$

The resulting baseline-shifted top-*K* objective is

$$J_{\text{RL}}≜\frac{1}{K}\sum_{i=1}^{N}\left(r_{i}-B_{\text{top}K}\right)1\left\{r_{i}\geq B_{\text{top}K}\right\}=\frac{1}{K}\sum_{i=1}^{K}\left(r_{\left(i\right)}-B_{\text{top}K}\right)$$ (20)

so that only rollouts in the empirical top quantile contribute, with non-negative shifted returns, while all remaining rollouts are excluded from this objective.

#### Entropy bonus

To encourage exploration in the hybrid action space, we add an entropy bonus computed along construction steps. Using the per-state hybrid policy entropy $H_{\pi_{\phi }}^{w}\left(s\right)$ defined in (*15*), we form

$$J_{H}^{w}≜E_{\tau }[\sum_{t=0}^{m}H_{\pi_{\phi }}^{w}\left(s_{t}\right)]$$ (21)

and weight it with a training coefficient $\lambda_{H}\geq 0$. The gradient of this entropy regularization term is implemented using a pathwise stop-gradient approximation. This omits the score-function cross term yielding a lower-variance (but biased) entropy-gradient estimator.

#### Self-imitation learning

To prevent “forgetting” rare but valuable solutions, we maintain a hall-of-fame replay buffer containing the best complete CRNs observed so far. Let $S_{k}$ denote the set of complete CRNs sampled in episode *k*, and define the cumulative sample set up to episode *k* as $S_{\leq k}=\cup_{k=1}^{k}S_{\bar{k}}$. For a hall-of-fame capacity $k_{\text{HOF}}$, we define

$${\text{HOF}}_{k}≜\text{Top}K_{k_{\text{HOF}}}(S_{\leq k};R)$$ (22)

i.e., the set of the $k_{\text{HOF}}$ highest-reward complete CRNs observed so far. Within the current batch, let

$$R_{m}^{*}≜\underset{s\in S_{k}}{\text{max}}\;R(s)$$ (23)

be the best reward achieved in the current iteration. We then add a SIL objective that increases the likelihood of sampling hall-of-fame solutions only when they outperform the current batch best

$$\begin{array}{c} J_{\text{SIL}}(\phi )≜-E_{s∼{\text{HOF}}_{k}}[{\left(R(s)-R_{t}^{*}\right)}_{+}\;\text{log}{\bar{p}}_{\phi }(s)]\\ \text{with}\;{\left(x\right)}_{+}≜\text{max}(0,x) \end{array}$$ (24)

where ${\bar{p}}_{\phi }(s)$ denotes the probability (under the current policy) of generating the complete CRN *s*, equivalently the likelihood of the action sequence that constructs *s*, with per-step log-probabilities summed in log-space as described above.

#### Final objective

We combine the risk-sensitive RL term, the entropy bonus, and SIL

$$J\left(\phi \right)≜J_{\text{RL}}(\phi )+\lambda_{H}J_{H}^{w}\left(\phi \right)+\lambda_{\text{SIL}}J_{\text{SIL}}(\phi )$$ (25)

and optimize the agent by minimizing the corresponding loss L(ϕ)≜−J(ϕ). The hyperparameters are $\left(p,k,w,\lambda_{H},\lambda_{\text{SIL}},k_{\text{HOF}}\right)$.

### Task-dependent loss functions

Let N denote an input-output CRN. Given an input signal *u* and initial condition $x_{0}$, we denote the (vector-valued) network output trajectory by $y(t;x_{0},u)$; we omit $x_{0}$ and/or u from the notation when they are fixed by the task. Each task defines a loss functional $L_{\text{task}}(N)$ that quantifies how well N matches a desired behavior. Unless stated otherwise, we measure output mismatch with an $\ell_{1}$ error

$$e(t)≜∥y(t;x_{0},u)-r{∥}_{1}$$

where *r* denotes a desired reference (e.g., a setpoint or target trajectory). When performance must hold across multiple tested conditions (inputs, disturbances, and/or initial conditions), we aggregate by averaging over the corresponding evaluation set.

For time-integrated objectives, we use a weighted integral operator

$$I[e]≜\int_{0}^{t_{f}}w_{d}(t)e(t)\mathrm{dt}$$ (26)

$$w_{d}(t)=\left\{\begin{array}{ll} 0.25, & 0\leq t<t_{f}/5,\\ 1, & t_{f}/5\leq t<4t_{f}/5,\\ 2, & 4t_{f}/5\leq t\leq t_{f} \end{array}\right.$$ (27)

where $t_{f}$ is the final simulation time and $w_{d}(t)$ emphasizes late-time accuracy (steady-state performance) while still penalizing transient error. Next, we specify the task-specific losses used in this work.

#### Dose-response shaping

For this task, we fix the initial condition to $x_{0}=0$. For each tested input level u∈U, we define the input-conditional loss

$$L_{\text{DR}}^{u}(N)≜I[∥y(t;u)-r(u){∥}_{1}]$$ (28)

where *r*(*u*) is the target dose-response function. The overall dose-response loss averages across the evaluated input set

$$L_{\text{DR}}(N)≜\frac{1}{|U|}\sum_{u\in U}L_{\text{DR}}^{u}(N)$$ (29)

#### Robust perfect adaptation

For this task, we evaluate performance across setpoints $u_{1}\in U_{1}$ and disturbances $u_{2}\in U_{2}$, with reference $r\left(u_{1}\right)$ independent of $u_{2}$. We define the overall RPA loss by averaging the time-integrated tracking error over all tested $\left(u_{1},u_{2}\right)$ conditions

$$L_{\text{RPA}}\left(N\right)≜\frac{1}{|U_{1}|\;|U_{2}|}\sum_{u_{1}\in U_{1}}\;\sum_{u_{2}\in U_{2}}I\left[∥y\left(t;u_{1},u_{2}\right)-r\left(u_{1}\right){∥}_{1}\right]$$ (30)

#### Fate decision circuits

For this task, we evaluate fate decisions across a set of initial conditions $x_{0}\in X_{0}$, where each $x_{0}$ is assigned a basin-specific reference trajectory $r(x_{0})$. We define the overall loss by averaging the time-integrated tracking error over all tested initial conditions

$$L_{\text{Fate}}(N)≜\frac{1}{|X_{0}|}\sum_{x_{0}\in X_{0}}I\left[∥y\left(t;x_{0}\right)-r\left(x_{0}\right){∥}_{1}\right]$$ (31)

#### Logic circuits

For each truth-table condition $c\in C_{\text{LOGIC}}$, we measure terminal-time disagreement at $t_{f}$ by

$$L_{\text{LOGIC}}^{c}\left(N\right)≜{\left\|y\left(t_{f};c\right)-r\left(c\right)\right\|}_{1}$$ (32)

We then average across all truth-table conditions

$$L_{\text{LOGIC}}(N)≜\frac{1}{|C_{\text{LOGIC}}|}\sum_{c\in C_{\text{LOGIC}}}L_{\text{LOGIC}}^{c}\left(N\right)$$ (33)

#### Oscillators

To promote sustained periodic behavior, we maximize a periodicity index computed from the normalized autocorrelation of the centered output. For a scalar output *y*(*t*), define the time-averaged mean

$$\bar{y}≜\frac{1}{t_{f}}\int_{0}^{t_{f}}y(t)\mathrm{dt}$$ (34)

the centered signal

$$y_{c}(t)≜y\left(t\right)-\bar{y}$$ (35)

and the normalized autocorrelation (for lags τ≥0)

$$R_{y}(\tau )≜\frac{\int_{0}^{t_{f}-\tau }\bar{y}\left(t\right)\bar{y}\left(t+\tau \right)\mathrm{dt}}{\int_{0}^{t_{f}}{\bar{y}}^{2}\left(t\right)\mathrm{dt}}$$ (36)

We then define the periodicity index as the height of the first positive-lag local maximum of $R_{y}$

$$\rho_{1}[y]≜\underset{\tau >0}{\text{max}}\left\{R_{y}(\tau \right)|R_{y}^{\prime }\left(\tau \right)=0,R_{y}^{\prime \prime }\left(\tau \right)<0^{\prime }\}$$ (37)

In addition to periodicity, we constrain the oscillation center (time average) to match a prescribed target $\mu^{⋆}\in \mathbb{R}$. We thus define the oscillator loss

$$L_{\text{Osc}}\left(N\right)≜\left(1-\rho_{1}\left[y\right]\right)+\lambda_{\mu }|\bar{y}-\mu^{⋆}|$$ (38)

where $\lambda_{\mu }\geq 0$ weights the center-matching term.

#### Oscillators with frequency tuning

For frequency-tunable oscillators, we include terms that (i) match a target frequency and (ii) discourage amplitude damping.

We estimate the oscillation period by averaging successive interpeak intervals. Let $t_{i}$ denote successive peak times of $|y_{c}(t)|$, let $n_{p}$ be the number of detected peaks in $\left[0,t_{f}\right]$, and define

$$T\left[y\right]≜\frac{1}{n_{p}-1}\sum_{i=1}^{n_{p}-1}\left(t_{i+1}-t_{i}\right)$$ (39)

To quantify damping, define peak amplitudes $A_{i}≜|y_{c}(t_{i})|$ and the damping index

$$\zeta [y]≜\frac{1}{n_{p}-1}\sum_{i=1}^{n_{p}-1}\frac{A_{i}}{A_{i+1}}$$ (40)

For a single evaluated condition u∈U (where *u* specifies the target frequency), we define the per-condition loss

$$L_{\text{OscF}}^{u}(N)≜\left(1-\rho_{1}[y\left(\cdot ;u\right)]\right)+\lambda_{f}|\frac{1}{T[y(\cdot ;u)]}-u|+\lambda_{d}\left(\zeta \left[y\left(\cdot ;u\right)\right]-1\right)$$ (41)

where $\rho_{1}[\cdot ]$ is the periodicity index defined above, and $\lambda_{f},\lambda_{d}\geq 0$ are weights. The overall loss averages across the evaluated conditions

$$L_{\text{OscF}}(N)≜\frac{1}{|U|}\sum_{u\in U}L_{\text{OscF}}^{u}\left(N\right)$$ (42)

#### Habituation and sensitization losses

In this application, we target two adaptive response classes—habituation and sensitization (*85*, *86*)—using a common loss construction based on three qualitative hallmarks: (i) a systematic change in response amplitude across repeated pulses within a pulse train (decreasing for habituation, increasing for sensitization), (ii) recovery of the output toward its prestimulus fixed point after a sufficiently long OFF gap (spontaneous recovery), and (iii) a stronger cumulative effect under higher-frequency stimulation (frequency sensitivity). The two tasks differ only in the direction of the within-train peak progression; all other terms are shared.

We evaluate these hallmarks over input levels u∈U and pulse shapes p∈P, where each pulse shape $p=\left(t_{\text{on}},t_{\text{off}}\right)$ defines both a stimulation frequency and a duty cycle. Each condition (u,p) consists of three phases: a pre-gap pulse train, an OFF gap of duration $t_{\text{gap}}$, and a post-gap pulse train (see fig. S5, B and C, red pulses). Let y(t;u,p) denote the output trajectory, and let $a_{k}^{\text{pre}}\left(u,p\right)$ and $a_{k}^{\text{post}}\left(u,p\right)$ denote the detected peak amplitudes (measured relative to the prestimulus fixed point) during the pre-gap and post-gap pulse trains, respectively.

To treat habituation and sensitization in a unified way, we define the sign

$$s≜\{\begin{array}{cc} +1, & \text{habituation},\\ -1, & \text{sensitization} \end{array}$$ (43)

so that the desired trend is always encoded by

$$s\left(a_{k+1}-a_{k}\right)\leq 0$$

that is, decreasing peaks for habituation and increasing peaks for sensitization.

For a fixed condition (u,p), let $n_{\text{pre}}$ be the number of detected peaks in the pre-gap pulse train, and define the signed consecutive-peak ratios

$$r_{k}(u,p)≜{\left(\frac{a_{k+1}^{\text{pre}}(u,p)}{a_{k}^{\text{pre}}(u,p)+\varepsilon }\right)}^{s},\;\;k=1,\ldots ,n_{\text{pre}}-1$$ (44)

where ε>0 is a small constant for numerical stability. We quantify the within-train trend by the log-median of these ratios

$$L_{\text{trend}}^{u,p}(N)\;≜\;\text{log}\;(\text{median}{\left\{r_{k}(u,p)\right\}}_{k=1}^{n_{\text{pre}}-1}+\varepsilon )$$ (45)

This term is small when the pre-gap pulse amplitudes evolve in the desired direction.

To enforce spontaneous recovery, let $t_{\text{gap}}^{-}$ denote the end of the OFF gap and let $y_{\text{fp}}(u)$ be the prestimulus fixed-point output under zero input. We define

$$L_{\text{gap}}^{u,p}\left(N\right)\;≜\;|y(t_{\text{gap}}^{-};u,p)-y_{\text{fp}}(u)|$$ (46)

$$+{\left[a_{1}^{\text{pre}}\left(u,p\right)-a_{1}^{\text{post}}(u,p)\right]}_{+}$$ (47)

which jointly encourages the output to relax back toward its original fixed point during the gap and to recover a comparable first-pulse response after the gap.

To reward stronger adaptation under higher-frequency stimulation, we define the signed adaptation score

$$\Delta \left(u,p\right)≜-s\frac{a_{n_{\text{pre}}}^{\text{pre}}\left(u,p\right)-a_{1}^{\text{pre}}\left(u,p\right)}{a_{1}^{\text{pre}}\left(u,p\right)+\varepsilon }$$ (48)

so that larger Δ(u,p) always corresponds to a stronger effect, whether habituation (larger decrease) or sensitization (larger increase). Ordering pulse shapes by increasing frequency as $p_{1},\ldots ,p_{|P|}$, we penalize violations of monotonic strengthening

$$L_{\text{freq}}^{u}\left(N\right)≜\frac{1}{|P|-1}\sum_{j=1}^{|P|-1}{\left[\Delta \left(u,p_{j}\right)-\Delta (u,p_{j+1})\right]}_{+}$$ (49)

with $L_{\text{freq}}^{u}\left(N\right)\equiv 0$ when ∣P∣=1.

The per-condition loss combines the within-train trend and spontaneous-recovery terms

$$L_{\text{Hab}/\text{Sens}}^{u,p}\left(N\right)≜L_{\text{trend}}^{u,p}\left(N\right)+\lambda_{\text{gap}}L_{\text{gap}}^{u,p}(N)$$ (50)

and the overall loss averages across inputs and pulse shapes, with an additional frequency-sensitivity penalty

$$L_{\text{Hab}/\text{Sens}}(N)≜\frac{1}{|U|\;|P|}\sum_{u\in U}\sum_{p\in P}L_{\text{Hab}/\text{Sens}}^{u,p}\left(N\right)$$ (51)

$$+\lambda_{\text{freq}}\frac{1}{|U|}\sum_{u\in U}L_{\text{freq}}^{u}(N)$$ (52)

where $\lambda_{\text{gap}},\lambda_{\text{freq}}\geq 0$ are weighting coefficients.

#### Stochastic objectives (mean and noise)

For stochastic CRNs, we optimize moments of the output. Under an evaluation condition c∈C, let

μ(t;c)≜E[y(t;c)] (53)

denote the output mean. We define the mean-tracking loss

$$L_{\text{MEAN}}^{c}\left(N\right)≜I[∥\mu \left(t;c\right)-r\left(c\right){∥}_{1}]$$ (54)

To penalize variability, we use the (component-wise) CV

$$\text{CV}\left(t;c\right)≜\frac{\sigma \left(t;c\right)}{\mu \left(t;c\right)+\varepsilon }$$ (55)

where σ(t;c) is the output standard deviation and ε>0 avoids division by zero. The variability loss is

$$L_{\text{CV}}^{c}\left(N\right)≜I[∥\text{CV}\left(t;c\right){∥}_{1}]$$ (56)

We combine the two objectives as

$$L_{\text{MEAN}+\text{CV}}^{c}\left(N\right)≜L_{\text{MEAN}}^{c}\left(N\right)+\lambda_{\text{CV}}L_{\text{CV}}^{c}(N)$$ (57)

with $\lambda_{\text{CV}}\geq 0$. The overall stochastic loss averages across evaluated conditions

$$L_{\text{MEAN}+\text{CV}}(N)≜\frac{1}{|C|}\sum_{c\in C}L_{\text{MEAN}+\text{CV}}^{c}\left(N\right)$$ (58)

(or, when conditions are grouped, by averaging within groups and then across groups).

### Size of the search space

Candidate topologies scale combinatorially with the reaction-template library. Let L be the library of admissible reaction templates and let $L_{0}\subseteq L$ denote templates fixed a priori (e.g., those in the starter I/O CRN) or excluded from sampling. The effective library is $L^{-}=L\backslash L_{0}$. Let $M≜|L^{-}|$ be the size of the effective library. Appending *m* reactions corresponds to selecting a size-*m* subset of $L^{-}$, so the number of topologically distinct candidates is

$$n_{\text{CRN}}(m,M)=\left(\begin{array}{c} M\\ m \end{array}\right)$$ (59)

The library size *M* depends on the reaction family and the number of species.

#### Mass-action kinetics

Under mass-action kinetics, a template reaction is specified by a reactant and a product complex (including the null complex). We say that the reaction is of doubly order *o* if the reactant and product complexes contain at most *o* molecules drawn from a species set of size *n*. Therefore, given *n* and *o*, the number of all possible complexes is

$$n_{c}(n,o)=\sum_{i=0}^{o}\text{MultiSet}\left(n,i\right)$$ (60)

where

$$\text{MultiSet}\left(a,b\right)≜\left(\begin{array}{c} a+b-1\\ b \end{array}\right)$$ (61)

denote the number of multisets of cardinality *b* drawn from a set of size *a*. It can be shown that the sum can be simplified to yield

$$n_{c}(n,o)=\left(\begin{array}{c} n+o\\ n \end{array}\right)$$ (62)

Last, pairing distinct reactant/product complexes yields

$$M=n_{c}(n,o)\left[n_{c}(n,o\right)-1]+1$$ (63)

where the final term accounts for the null reaction (empty-to-empty).

For instance, for *n* = 3 species, order *o* = 2, and *m* = 5 reactions, the library size is *M* = 91, and the number of possible CRN topologies is *n*_CRN_ = 46,504,458. This excludes the rate parameters that further expand the space. Adding one more reaction, that is, *m* = 6, without changing the number of species and reaction order increases the possibilities by over an order of magnitude to *n*_CRN_ = 666,563,898. Allowing *n* = 4 species increases the library size to *M* = 211 and the number of CRN topologies to *n*_CRN_ = 114,081,819,852.

#### Hill-type kinetics

For Hill-type (production-only) templates, the number of distinct product complexes of size up to $n_{\text{prod}}$ is

$$\sum_{n_{i}=1}^{n_{\text{prod}}}\text{MultiSet}\left(n,n_{i}\right)$$ (64)

Signed regulator (activator or repressor enzyme) sets of size up to $n_{\text{REG}}$ contribute a factor

$$\sum_{n_{i}=1}^{n_{\text{REG}}}\left(\begin{array}{c} n\\ n_{i} \end{array}\right)2^{n_{i}}$$ (65)

so the Hill library size is

$$M=\sum_{n_{i}=1}^{n_{\text{prod}}}\text{MultiSet}\left(n,n_{i}\right)\sum_{n_{i}=1}^{n_{\text{REG}}}\left(\begin{array}{c} n\\ n_{i} \end{array}\right)\;2^{n_{i}}$$ (66)

For example, allowing only on product per reaction ($n_{\text{prod}}=1$) and up to two regulators ($n_{\text{REG}}=2$), we have *M* = 432 reactions.

### Implementation and computational resources

The GenAI-Net implementation will be made fully available on GitHub with the final version of this preprint. The software is written in Python, using PyTorch for the reinforcement-learning loop and PyCUDA for GPU-accelerated stochastic simulation.

Deterministic analyses other than the dose-response experiments were run on a workstation (WS1) equipped with an AMD Ryzen Threadripper PRO 7985WX CPU and an NVIDIA RTX 4500 Ada GPU, with computations distributed across 64 CPU cores. Stochastic simulations, which are predominantly GPU-bound, were performed on a machine (WS2) equipped with an Intel Xeon W-2123 CPU and NVIDIA Titan RTX GPUs; these resources were provided by the D-BSSE ETH Zurich. Dose-response experiments were conducted on a computing cluster (CLS) with Intel Xeon Gold 6330 CPUs using 40 CPU cores and with Nvidia A800 GPUs, provided by the Academy of Mathematics and Systems Science, Chinese Academy of Sciences.

Deterministic simulations for logic-circuit training runs were executed on WS1. For the logic-circuit experiments (five species and six reactions), training with batch size 1024 for 300 epochs required ∼5 hours. Stochastic simulations were executed on WS2: For all SSA-based evaluations, we used 1000 SSA simulations per batch element. The stochastic RPA experiment with CV reduction (batch size 160, 600 epochs) required ∼20 hours.

### User workflow

This section provides a high-level guide for applying our method. We outline how to define a problem, which hyperparameters matter in practice, how to configure logging, how to store trained models, and how to visualize results.

### Conceptualization of the problem

This is the first—and one of the most critical—steps for a successful application of our method. In our setting, it starts with choosing the application domain: whether to use a deterministic or stochastic CRN interpretation, and selecting a reaction library. These choices determine which backend is used to evaluate candidate CRNs inside the training loop.

To define the task, the user must specify (i) a loss function, (ii) a set of inputs, and (iii) initial conditions. Since many applications can be formulated as transient tracking problems, we provide utilities to construct distance-from-reference loss functions among others.

As an example, consider a task that searches for circuits exhibiting RPA. We used a single initial condition (the zero state), allowed deterministic CRNs with mass-action kinetics, selected a small set of inputs and disturbances ($u_{\text{in}}\in \left\{1,2,3\right\}$ and $u_{\text{disturbance}}\in \left\{0.5,1,1.5\right\}$), and defined the loss as the time integral of the tracking error between a designated output species and a reference proportional to $u_{\text{in}}$ as detailed in a previous section. This encourages both accurate setpoint tracking and fast responses.

### Hyperparameters

After defining the problem, the method hyperparameters must be specified; in practice, only a small subset has a noticeable effect on performance. A compact reference is provided in Table 1, which reports the values used for this task (and can serve as a reasonable default configuration).

**Table 1.** Hyperparameters used for the GenAI-Net.

Hyperparameter	Description	Default value	Impact
Entropy			
$\lambda_{H}$	Weight of the entropy bonus to promote exploration.	5 × 10^−3^	High
*w*	Entropy weight encouraging diversity in proposed CRN parameters.	1/5	High
Risk			
*p*	Risk level: Use only the top *p*-percentile topologies to compute updates.	0.90	High
Self-imitation learning			
$\lambda_{\text{SIL}}$	Weight of the SIL loss to reduce forgetting by reinforcing past good solutions.	1.0	Low
$n_{\text{SIL-HOF}}$	Replay buffer size (hall-of-fame): Number of CRNs stored for SIL.	50	Low
Agent			
$n_{\text{epochs}}$	Number of training epochs.	300	Moderate
net_width	Hidden-layer width of the policy feed-forward network.	10,240	Low
net_depth	Number of hidden layers in the policy feed-forward network.	*5*	Low
batch_size	Mini-batch size.	1024	Moderate

### Training and logging

We provide an interface for experiment tracking via Comet.ml, enabling users to monitor training progress and to log each component of the objective (e.g., reward, entropy bonuses, and auxiliary terms) separately. In addition, we support task-dependent visualizations of candidate CRN behaviors. For example, in the setting considered above, we render the transient trajectories of the designated output species under multiple input levels and disturbance instances to directly assess tracking and robustness.

At the end of training, users can export the trained RL agent together with a snapshot of the hall of fame. The hall of fame stores the highest-performing CRNs encountered during optimization and should be interpreted as the primary output of the search procedure (i.e., a curated set of promising designs).

### Parameters of discovered I/O CRNs

All parameters for the discovered I/O CRNs presented in this manuscript are provided in the Supplementary Materials. We also provide an explicit rewriting of each CRN in terms of reaction equations (as a complement to the graphical representations).
