# Supplementary material for: GenAI-Net: A generative AI framework for automated biomolecular network design
Source: Sci Adv. 2026 Sep 30;12(40):eaeh8819. doi: 10.1126/sciadv.aeh8819 (PMC13626088; doi:10.1126/sciadv.aeh8819)
Supplement: Supplementary file 1 [file sciadv.aeh8819_sm.pdf]

Science Advances
AAAS

Supplementary Materials for

GenAI-Net: A generative AI framework for automated biomolecular network design

Maurice Filo *et al.*

Corresponding author: Mustafa Khammash, mustafa.khammash@bsse.ethz.ch

Sci. Adv. **12**, eaeh8819 (2026)
DOI: 10.1126/sciadv.aeh8819

This PDF file includes:

Figs. S1 to S13
Table S1
Supplementary Text

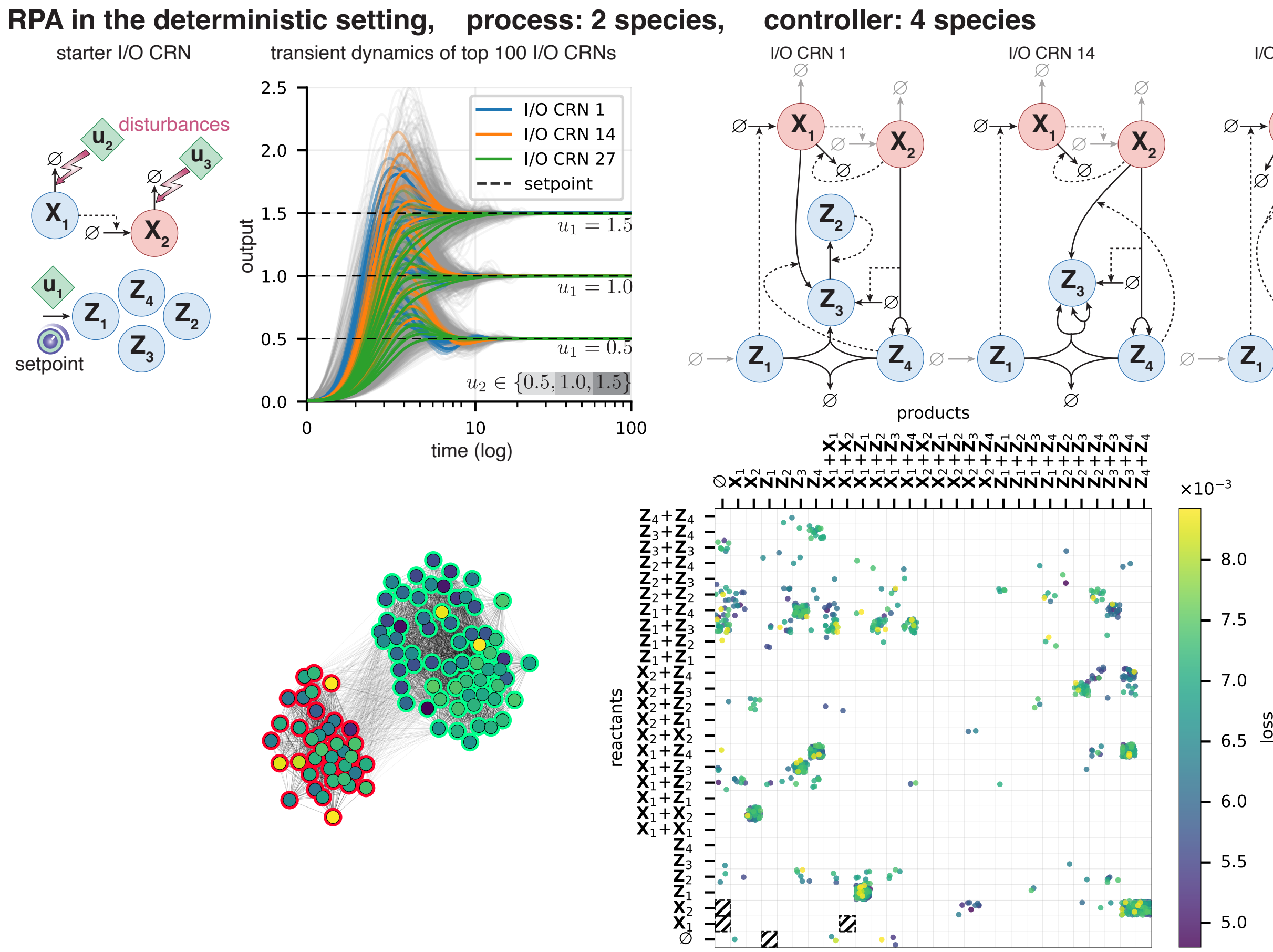

Figure S1: **Deterministic RPA with 6 species.** GenAI-Net scales to a higher-dimensional setting with two process species, the regulated output $\mathbf{X_2}$ (red) and an additional species $\mathbf{X_1}$, together with four controller species $\mathbf{Z_1}$–$\mathbf{Z_4}$ (blue). The setpoint is specified by u_1, while disturbances u_2 and u_3 modulate degradation of $\mathbf{X_1}$ and $\mathbf{X_2}$, respectively. Starting from this multi-species starter I/O CRN, GenAI-Net generates candidate I/O CRNs by appending up to eight doubly bimolecular mass-action reactions and optimizing the same setpoint-tracking objective as in Fig. 4A. The center plot shows output trajectories for all generated solutions (gray), with three representative networks highlighted (I/O CRNs 1, 14, and 27) and their corresponding topologies shown to the right. The graph visualization and reactant–product incidence map summarize topological diversity and reaction usage across the generated collection, as in Fig. 4A.

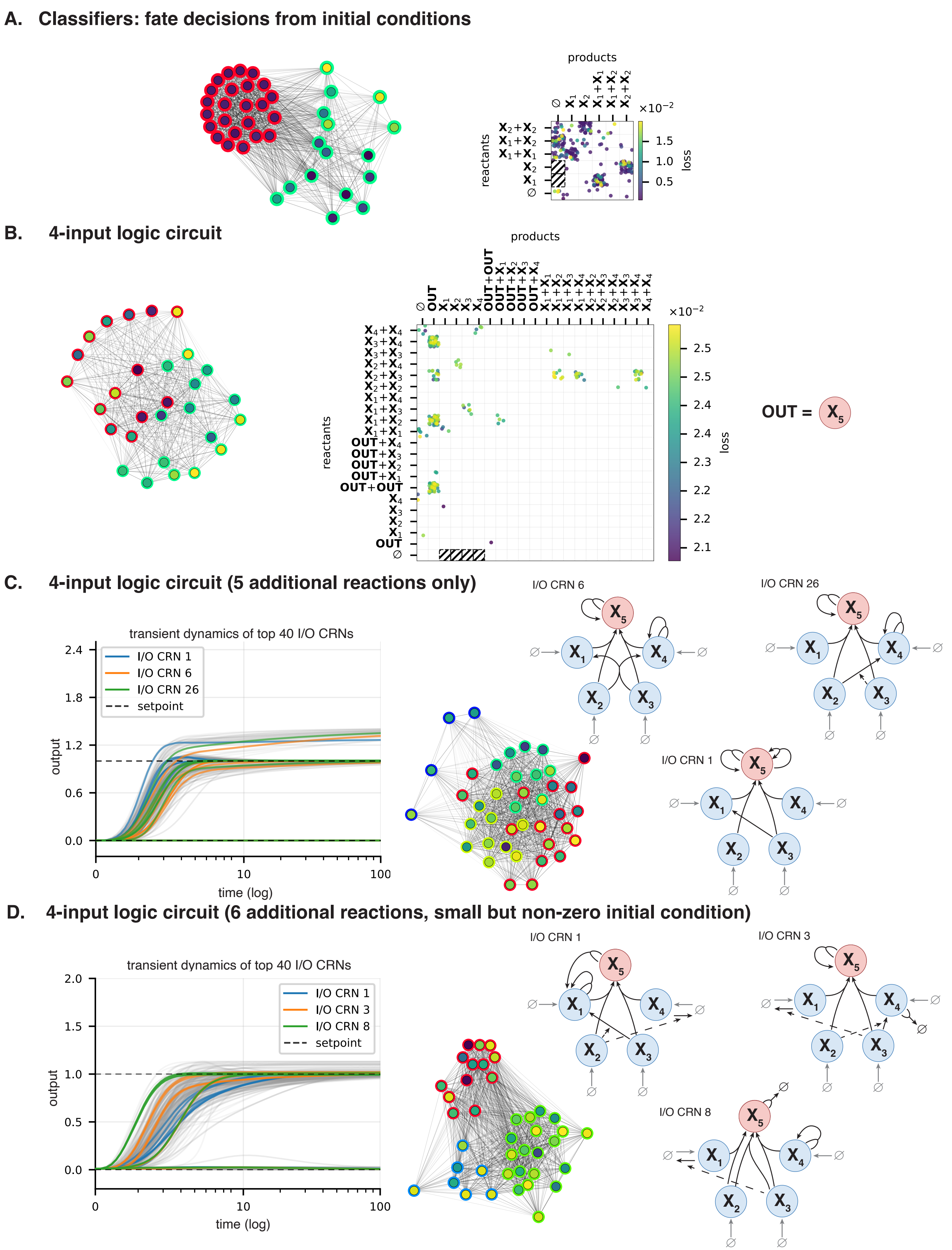

Figure S2: **Classifiers and logic circuits.** The figure caption is on the next page.

Figure S2: **Classifiers and logic circuits.** **(a)** and **(b)**: The graph visualization and reactant–product incidence map summarizing topological diversity and reaction usage across the generated collections of Fig. 6 and 7 respectively. **(c)** Restricting the search to at most 5 appended reactions (instead of 6 as in Fig. 7), circuits that remain correct under thresholding but exhibit a more analog implementation of the logic; in particular, highlighted examples show a noticeable overshoot above the nominal "high" level in the all-inputs-high condition, in contrast to the cleaner saturation obtained in Fig. 7 when allowing one additional reaction. **(d)** This panel is the same as Fig. 7 with only one difference: the initial conditions are not zero to avoid exploiting unstable fixed points at the origin. GenAI-Net also succeeded in generating diverse I/O CRNs under these conditions.

A. Reaction library with Hill kinetics (433 reactions)

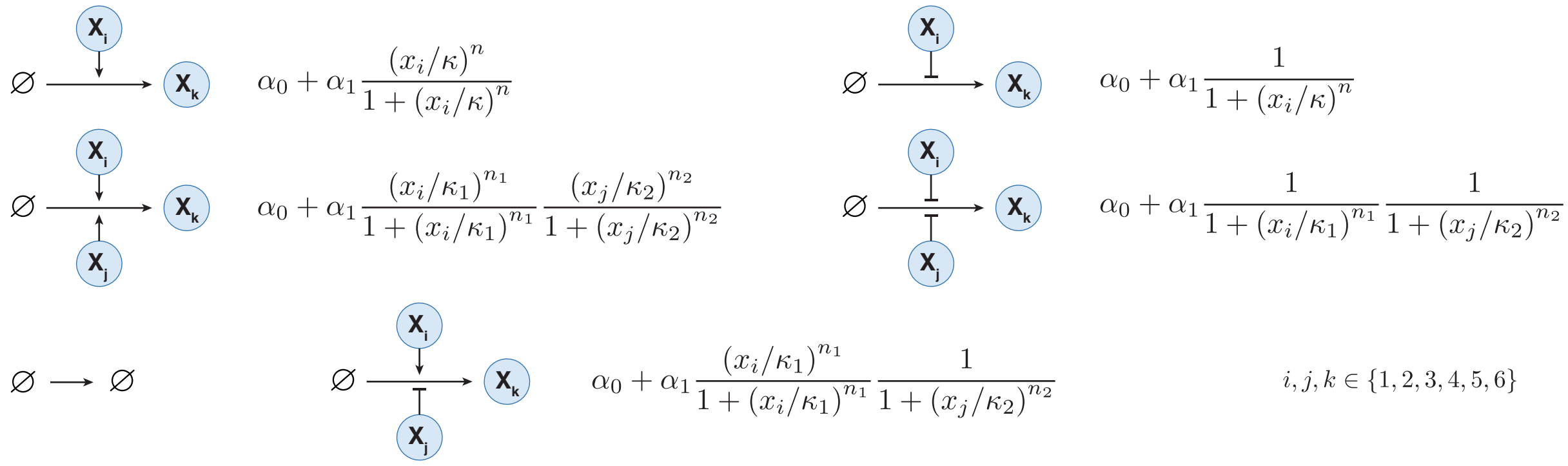

B. 4-input logic circuit (6 additional reactions) - Hill function $(u_1 \wedge u_2) \vee (u_2 \wedge u_3) \vee (u_3 \wedge u_4)$

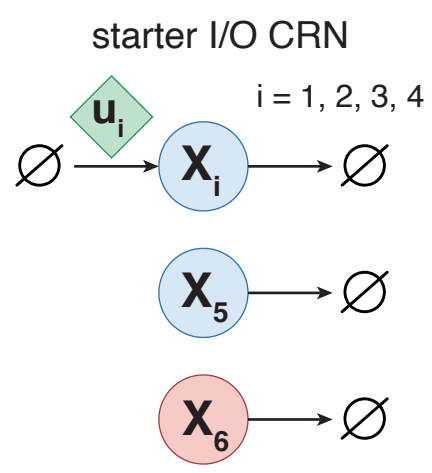

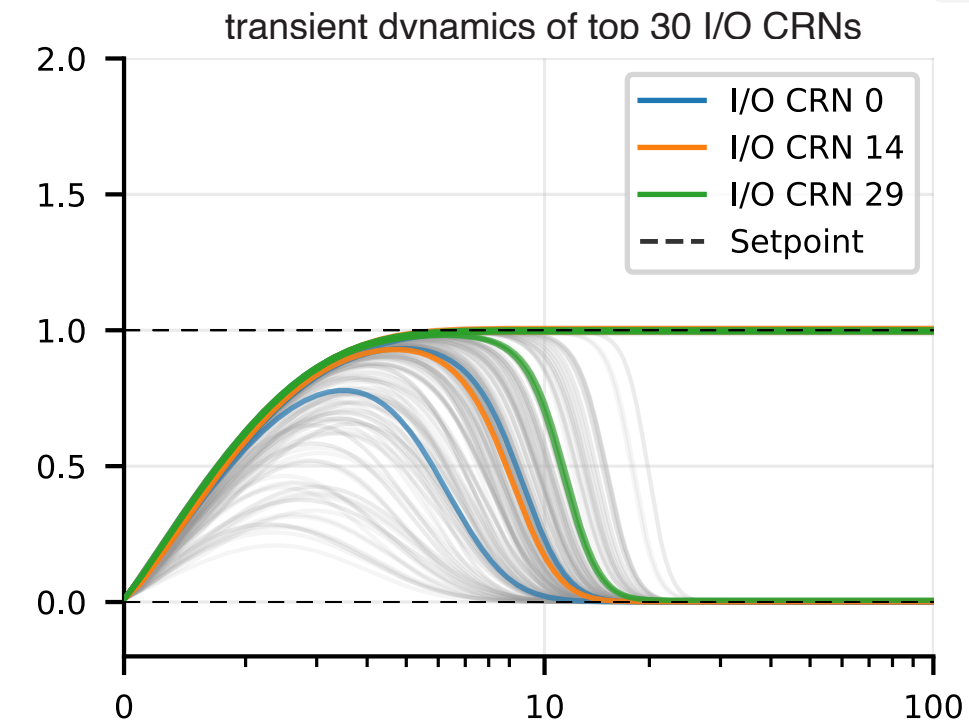

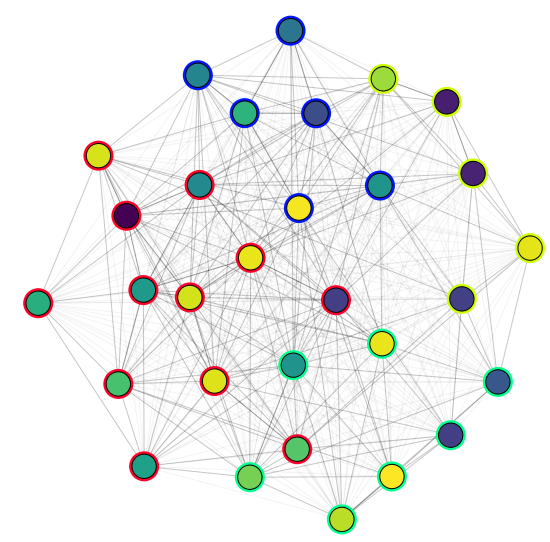

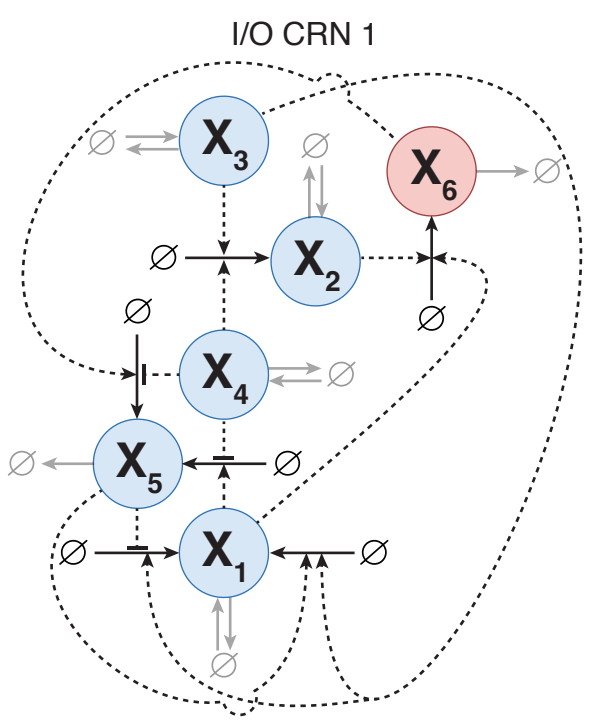

u_1	0	0	0	0	0	0	0	0	1	1	1	1	1	1	1	1
u_2	0	0	0	0	1	1	1	1	0	0	0	0	1	1	1	1
u_3	0	0	1	1	0	0	1	1	0	0	1	1	0	0	1	1
u_4	0	1	0	1	0	1	0	1	0	1	0	1	0	1	0	1
**Target**	0.00	0.00	0.00	1.00	0.00	0.00	1.00	1.00	0.00	0.00	0.00	1.00	1.00	1.00	1.00	1.00
**Output**	**0.00**	**0.00**	**0.00**	**1.00**	**0.00**	**0.00**	**1.00**	**1.00**	**0.00**	**0.00**	**0.00**	**1.00**	**1.00**	**1.00**	**1.00**	**1.00**

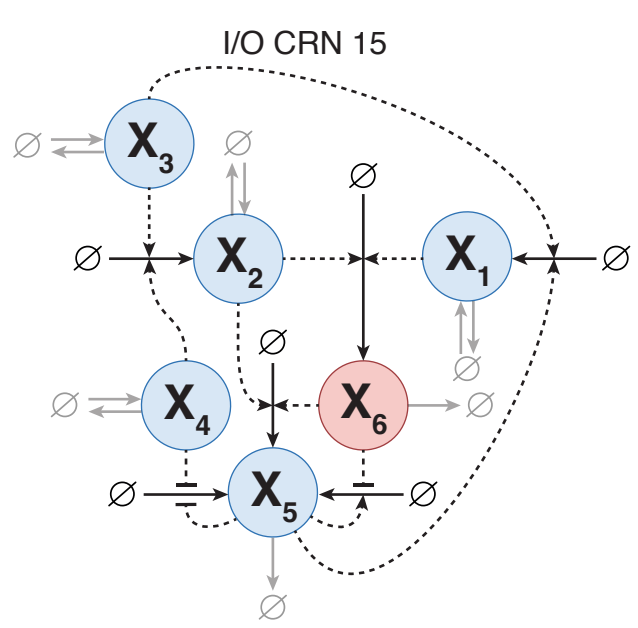

u_1	0	0	0	0	0	0	0	0	1	1	1	1	1	1	1	1
u_2	0	0	0	0	1	1	1	1	0	0	0	0	1	1	1	1
u_3	0	0	1	1	0	0	1	1	0	0	1	1	0	0	1	1
u_4	0	1	0	1	0	1	0	1	0	1	0	1	0	1	0	1
**Target**	0.00	0.00	0.00	1.00	0.00	0.00	1.00	1.00	0.00	0.00	0.00	1.00	1.00	1.00	1.00	1.00
**Output**	**0.00**	**0.00**	**0.00**	**1.00**	**0.00**	**0.00**	**1.00**	**1.01**	**0.00**	**0.00**	**0.00**	**1.00**	**0.99**	**0.99**	**1.00**	**1.01**

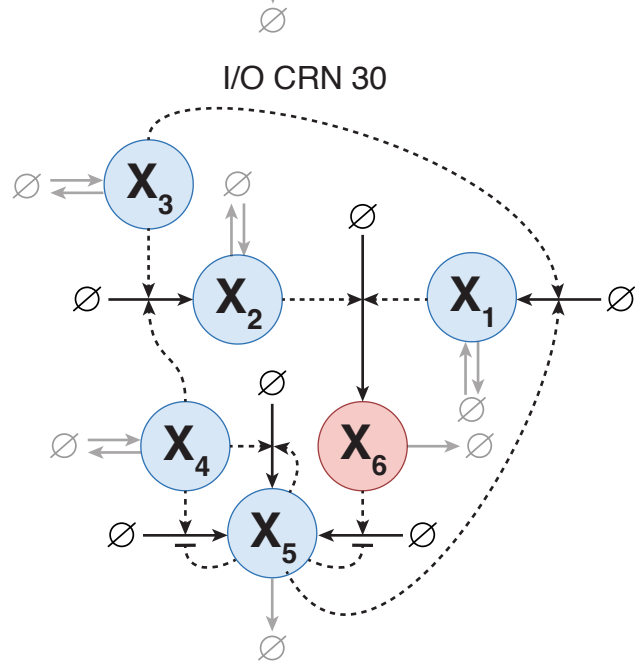

u_1	0	0	0	0	0	0	0	0	1	1	1	1	1	1	1	1
u_2	0	0	0	0	1	1	1	1	0	0	0	0	1	1	1	1
u_3	0	0	1	1	0	0	1	1	0	0	1	1	0	0	1	1
u_4	0	1	0	1	0	1	0	1	0	1	0	1	0	1	0	1
**Target**	0.00	0.00	0.00	1.00	0.00	0.00	1.00	1.00	0.00	0.00	0.00	1.00	1.00	1.00	1.00	1.00
**Output**	**0.00**	**0.00**	**0.00**	**1.00**	**0.01**	**0.01**	**0.99**	**1.00**	**0.00**	**0.00**	**0.00**	**1.00**	**0.99**	**0.99**	**1.00**	**1.00**

Figure S3: **Logic design with Hill-type kinetics.** The figure caption is on the next page

Figure S3: **Logic design with Hill-type kinetics. (A) Reaction library with Hill kinetics.** To test GenAI-Net with reactions governed by multiple parameters, we construct a reaction library of 433 admissible reaction templates whose propensities follow Hill-type activation and repression forms (schematics). These templates include single-enzyme and multi-enzyme Hill functions that modulate effective production rates, yielding a larger action space for logic synthesis. **(B) 4-input logic circuit using Hill functions.** GenAI-Net starts from a *starter I/O CRN* (left) containing species $\mathbf{X_1}$–$\mathbf{X_6}$, with four external inputs u_i ($i = 1, \ldots, 4$) that modulate the production rates of $\mathbf{X_1}$–$\mathbf{X_4}$. All species are also assumed to dilute at a constant rate. The objective is to implement the target 4-input Boolean function shown above the panel. GenAI-Net appends up to **6 additional reactions** selected from the Hill library and optimizes a terminal-time truth-table loss identical to that in Fig. 7. The center plot shows output transients for the **top 30** topologically unique generated I/O CRNs (gray), with three representative solutions highlighted (colored; I/O CRNs 1, 15, and 30) and their corresponding topologies shown to the left. The network visualization (right) summarizes topological diversity across the 30 solutions. The truth-table panels (bottom) compare target outputs to simulated outputs across all 2^4 input combinations for the representative networks, demonstrating accurate multi-input logic computation with Hill-type kinetics. Note that, compared to Fig. 7, this Hill library is less flexible: only production reactions can be selected, whereas degradation reactions are not available. This constraint makes the task more demanding and motivates inclusion of an additional intermediate species ($\mathbf{X_5}$) to achieve high-precision logic behavior.

Oscillators with input-tunable frequency

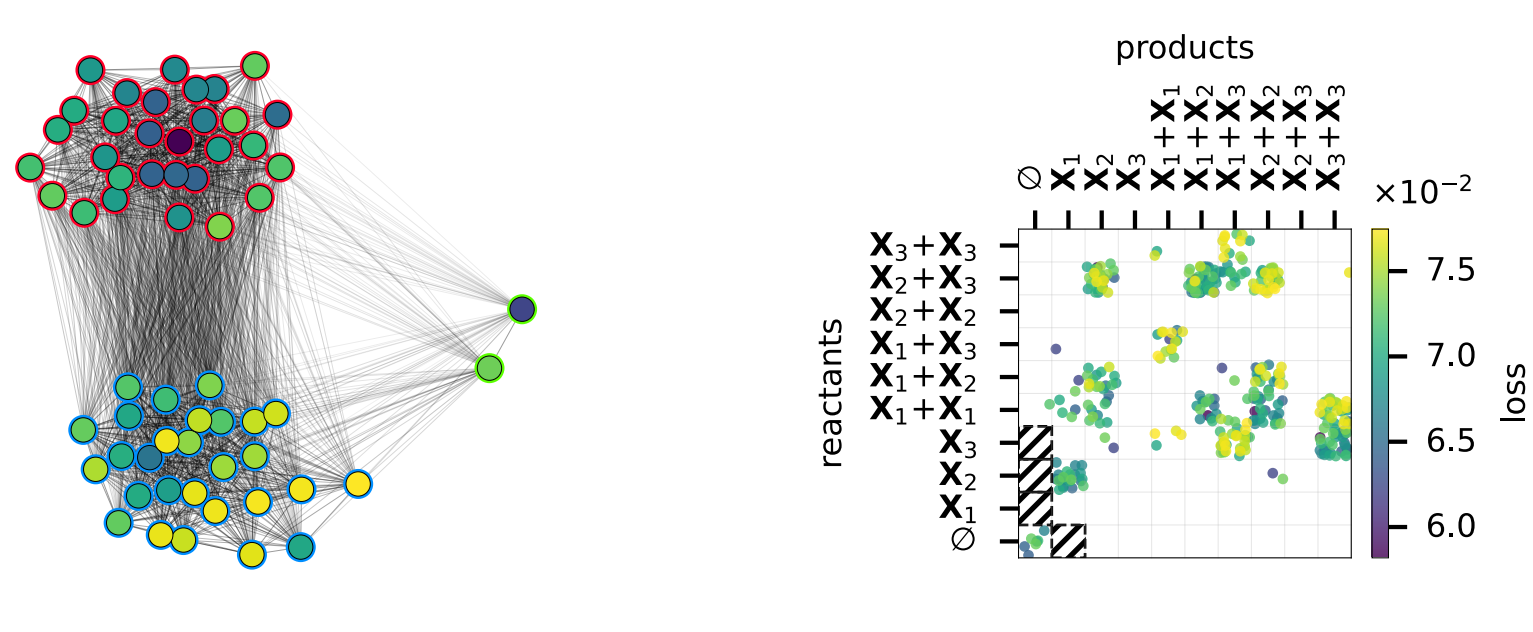

Figure S4: **Oscillators with input-tunable frequency.** The graph visualization and reactant–product incidence map summarizing topological diversity and reaction usage across the generated collections of Fig. 9.

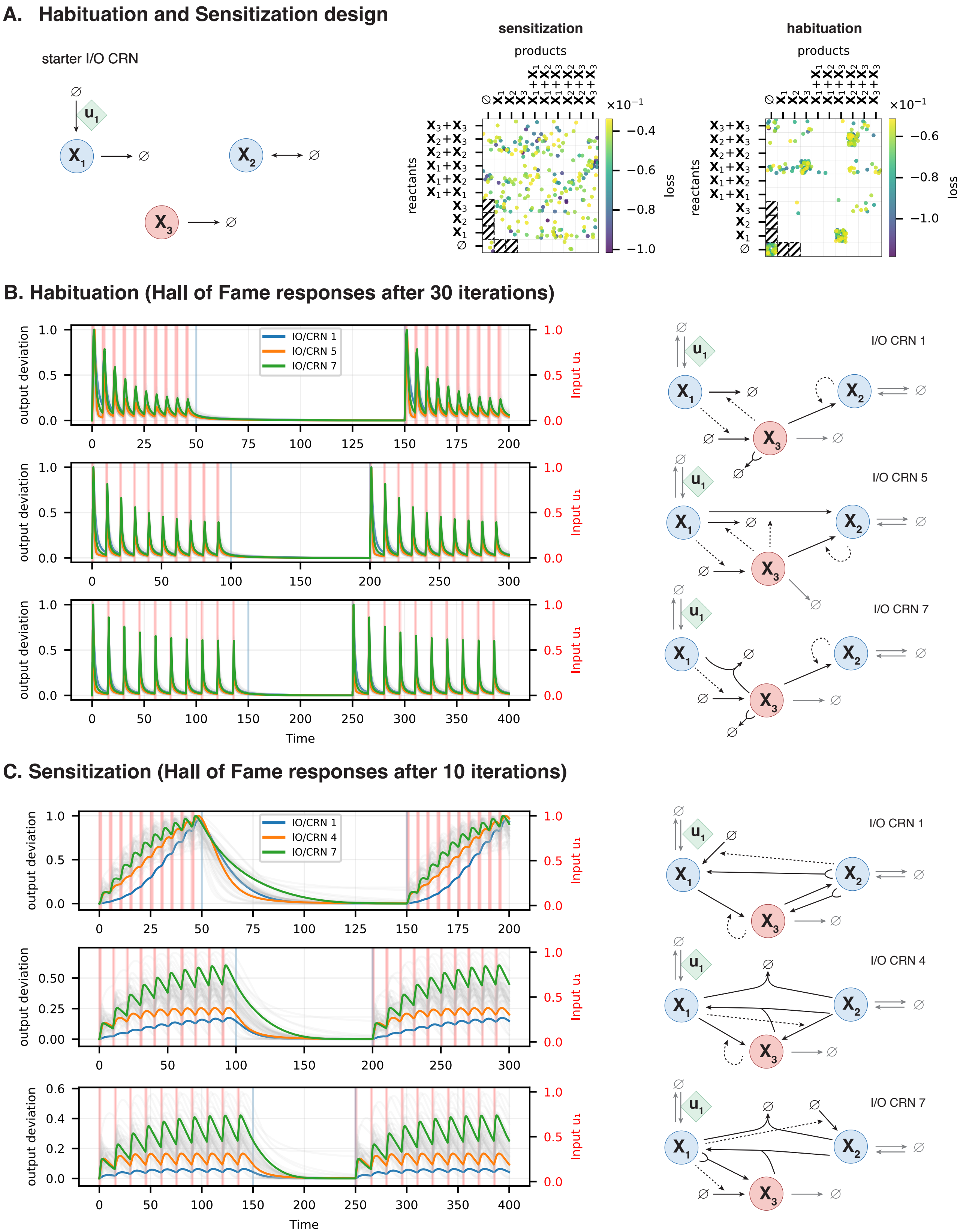

Figure S5: Molecular habituation and sensitization. The figure caption is on the next page.

Figure S5: **Design of biomolecular circuits exhibiting habituation and sensitization. (a) Problem setup and reaction signatures.** GenAI-Net starts from a *starter I/O CRN* with three species, an external input u_1, and a designated output species $\mathbf{X_3}$ (red). The design objective is to generate I/O CRNs whose output exhibits either *sensitization* (progressively increasing responses under repeated stimulation) or *habituation* (progressively decreasing responses), while preserving spontaneous recovery after an OFF gap (see Methods). The reactant–product incidence maps at right summarize reaction usage for the corresponding solution sets: each point denotes a reaction, and point color indicates the loss of I/O CRNs in which that reaction appears. **(b) Habituation.** After only 30 iterations, GenAI-Net identifies a hall-of-fame set of habituating I/O CRNs. The left panels show representative output trajectories for three highlighted networks (I/O CRNs 1, 5, and 7; colored curves), with the input pulse train u_1 overlaid in red. Across repeated pulses, the output peaks progressively attenuate, and after the OFF gap the response recovers before adapting again during the second pulse train, consistent with habituation and spontaneous recovery. Furthermore higher frequencies exhibit faster habituation. Gray trajectories indicate the remaining hall-of-fame solutions. The corresponding I/O CRN topologies are shown at right. These topologies show negative feedback structures, incoherent feedforward loop structure as reported in (*85, 86*), and buffering structures where $\mathbf{X_2}$ monitors the output and converts it to itself faster when the next pulse comes in. **(b) Sensitization.** After only 10 iterations, GenAI-Net likewise identifies a hall-of-fame set of sensitizing I/O CRNs. The left panels show representative trajectories for I/O CRNs 1, 4, and 7, again with the input pulse train overlaid in red. In contrast to (c), repeated stimulation now produces progressively larger output peaks, followed by recovery during the OFF gap and renewed sensitization under the second pulse train. The associated I/O CRN topologies are shown at right. Together, these results show that GenAI-Net can generate distinct circuit families that realize opposite forms of adaptive pulse-train response using the same overall design framework.

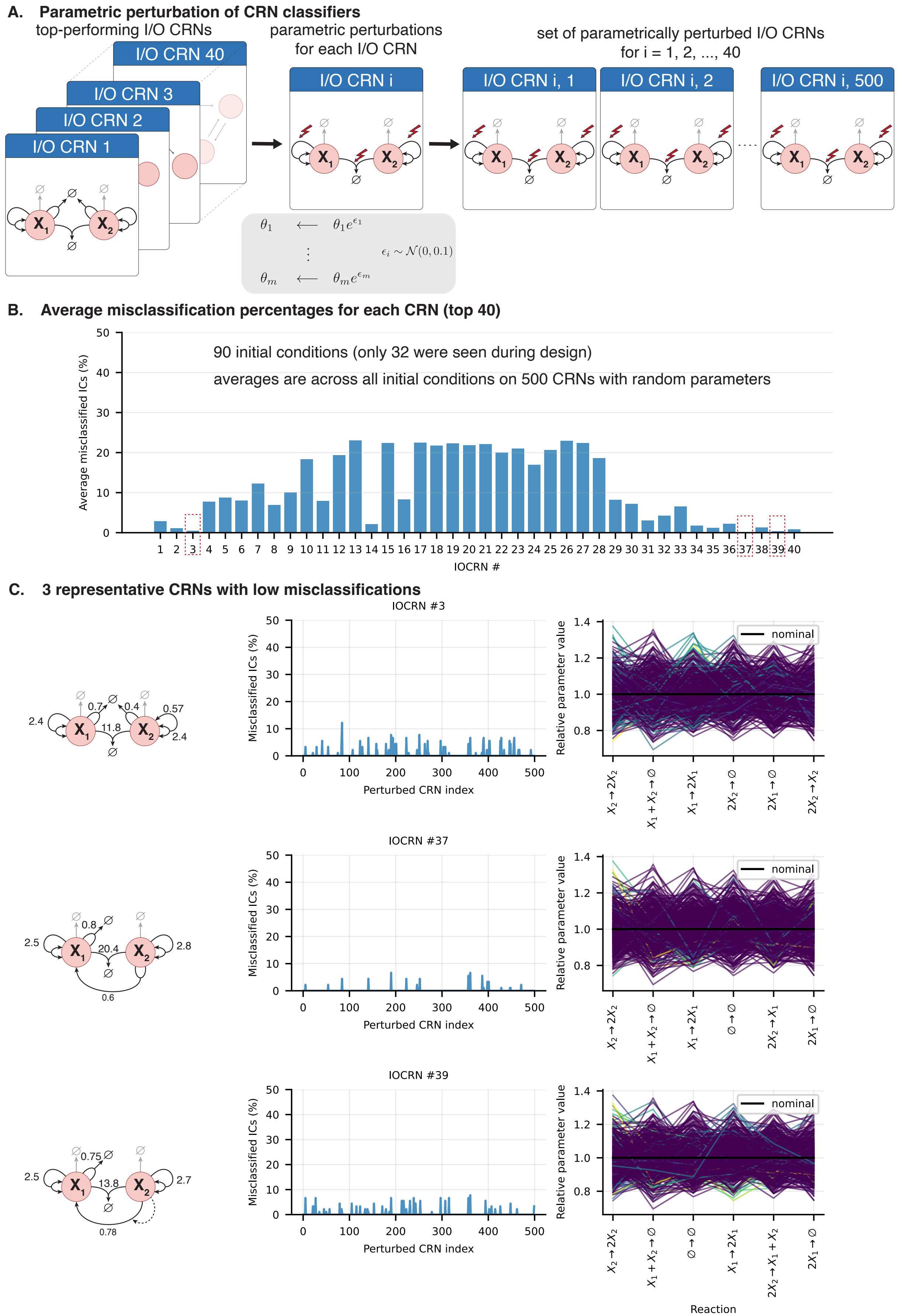

Figure S6: Parametric robustness of initial-condition classifiers implemented by chemical reaction networks. The figure caption is on the next page.

Figure S6: **Parametric robustness of initial-condition classifiers implemented by chemical reaction networks. (a) Robustness-analysis workflow.** The 40 top-performing, topologically distinct I/O CRNs were selected based on their nominal classification performance from Fig. 6. For each I/O CRN i, 500 independently perturbed variants were generated while preserving its reaction topology. Each reaction rate (excluding the starter reactions) was perturbed multiplicatively according to $\theta'_j = \theta_j e^{\epsilon_j}$, with independently sampled $\epsilon_j \sim \mathcal{N}(0, 0.1)$ and thus representing 10% relative parametric perturbations to all the parameters simultaneously. Lightning symbols indicate perturbed reaction-rate parameters. **(b) Mean percentage of misclassified initial conditions for each of the 40 nominal I/O CRNs after parameter perturbation.** Each bar is the average across the 500 independently perturbed variants and 90 initial conditions. The initial conditions form a grid as in Fig. 6. Only 32 of these conditions were used during CRN design; the remaining conditions test generalization beyond the design set. Low bars therefore indicate that the classifier's behavior is preserved despite random kinetic-parameter perturbations. Red dashed boxes identify the representative robust classifiers shown in panel c. **(c) Detailed robustness profiles for representative I/O CRNs 3, 37, and 39.** Left, reaction-network diagrams show the nominal topology and rounded kinetic parameters; gray reactions are the fixed starter reactions, while black reactions were perturbed. Middle, percentage of the 90 initial conditions misclassified by each of the 500 perturbed variants. Right, corresponding perturbed kinetic parameters normalized by their nominal values; each polyline represents one perturbed CRN, and the horizontal black line denotes the nominal parameter values. Together, these examples demonstrate that several independently discovered CRN topologies retain accurate initial-condition classification across broad simultaneous perturbations of their kinetic parameters even though this requirement was not explicitly encoded in the loss function.

A. Parametric perturbations: average frequency error and periodicity index (top 60)

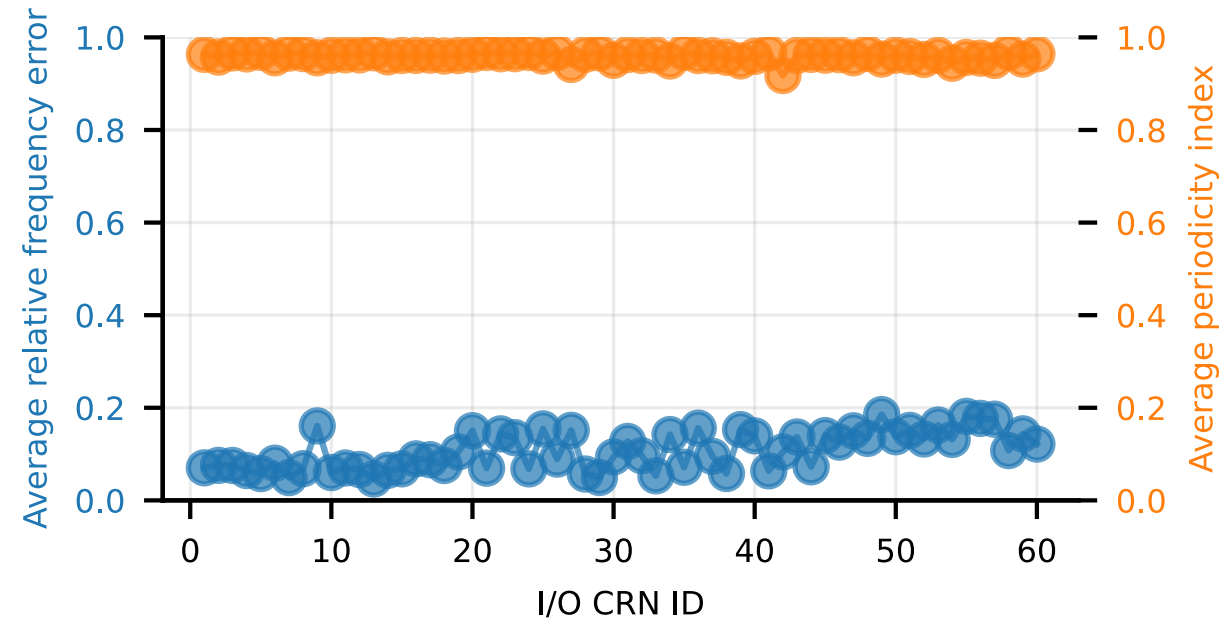

B. 3 representative CRNs with low frequency errors

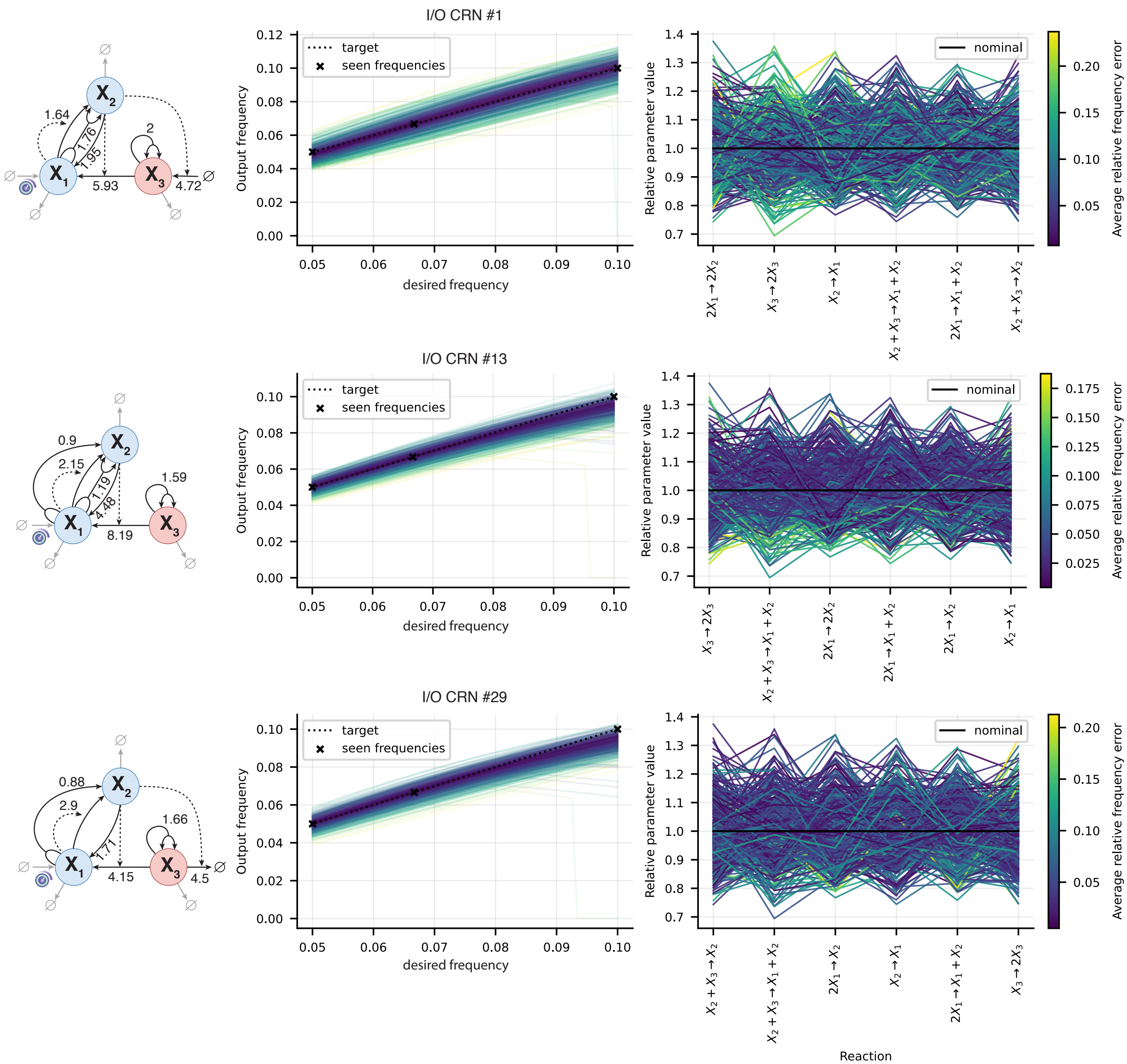

Figure S7: **Parametric robustness of frequency-tracking chemical oscillators. The figure caption is on the next page.**

Figure S7: **Parametric robustness of frequency-tracking chemical oscillators.** The parameter-perturbation procedure follows the workflow introduced in Fig. S6(a): The 60 top-performing, topologically distinct I/O CRNs were selected based on their nominal classification performance from Fig. 9. For each I/O CRN i, 500 independently perturbed variants were generated while preserving its reaction topology. Each reaction rate (excluding the starter reactions) was perturbed multiplicatively according to $\theta'_j = \theta_j e^{\epsilon_j}$, with independently sampled $\epsilon_j \sim \mathcal{N}(0, 0.1)$ and thus representing 10% relative parametric perturbations to all the parameters simultaneously. Each perturbed oscillator was evaluated at 100 desired frequencies (only 3 of which were seen during design) in the same way as in Fig. 9. **(a) Robustness of the 60 top-performing, topologically distinct frequency-tracking I/O CRNs.** Blue markers show the mean relative frequency error of the 500 perturbed variants of each nominal CRN, averaged across all tested desired frequencies. Orange markers show the corresponding mean periodicity index, where values approaching one indicate sustained periodic behavior. Thus, several frequency-tracking oscillators simultaneously exhibited maintained oscillations close to the desired input frequencies despite the parametric perturbations even though this was not encoded in the loss function. **(b) Detailed robustness profiles for three representative oscillators with low frequency errors: I/O CRNs 1, 13, and 29.** Left, reaction-network diagrams show the nominal CRN topologies and rounded kinetic parameters. Gray reactions denote the fixed starter reactions, whereas black reactions were subjected to parameter perturbations; $\mathbf{X_3}$ is the output species. Center, input-output frequency maps for the 500 perturbed variants. Each line represents one perturbed CRN evaluated over the full range of desired frequencies. The dotted diagonal indicates perfect frequency tracking, and crosses denote the three frequencies used during CRN design. Right, kinetic parameters of each perturbed variant normalized by their corresponding nominal values. Each polyline represents one perturbed CRN, and the horizontal black line denotes the nominal parameter vector. In the center and right plots, lines are colored by their average relative frequency error, linking changes in kinetic parameters to changes in frequency-tracking performance. Together, the results show that the selected CRNs generally retain sustained oscillations and accurate frequency tracking under simultaneous random perturbations of their kinetic parameters.

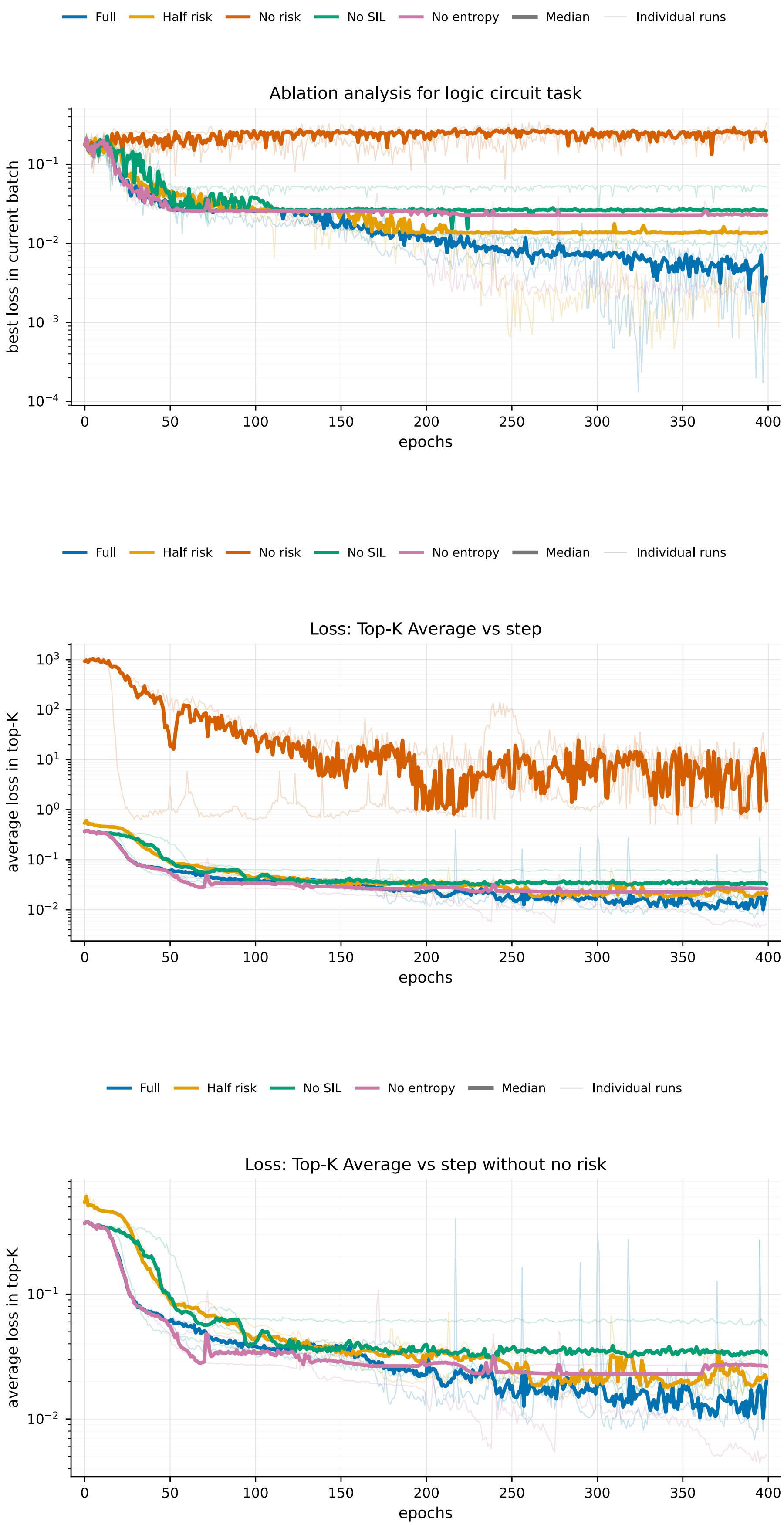

Figure S8: **Ablation analysis for the logic-circuit inference task. The figure caption is on the next page.**

Figure S8: **Ablation analysis for the logic-circuit inference task.** Training performance over 400 epochs for the complete GenAI-Net (Full) and four ablations: reducing the risk parameter from $r = 0.9$ to $r = 0.5$ (Half risk), removing risk seeking with $r = 0$ (No risk), disabling self-imitation learning (No SIL), and disabling entropy regularization (No entropy). The losses are plotted over the epochs; lower values indicate better logic-circuit performance. Thick colored curves show the median across three independent runs, while pale curves show individual runs (3 runs). All vertical axes use logarithmic scaling. Top: Best loss among the batch during each epoch. The full method achieves the lowest median batch-best loss by the end of training, whereas removing components substantially degrades performance and often gets stuck at local minima. Middle: Mean loss over the Top-K candidates, where $K = \lfloor 1280(1 - r) \rfloor$: $K = 127$ for Full (no ablation), No SIL, and No entropy; $K = 640$ for Half risk; and $K = 1280$ for No risk. The No risk condition remains markedly worse because its objective averages over the entire batch rather than emphasizing its best candidates. Bottom: The same Top-K metric with No risk omitted, revealing differences among the remaining conditions more clearly. The full method continues improving throughout training and ultimately attains the lowest median Top-K loss, while removing self-imitation learning or entropy regularization leads to earlier performance plateaus.

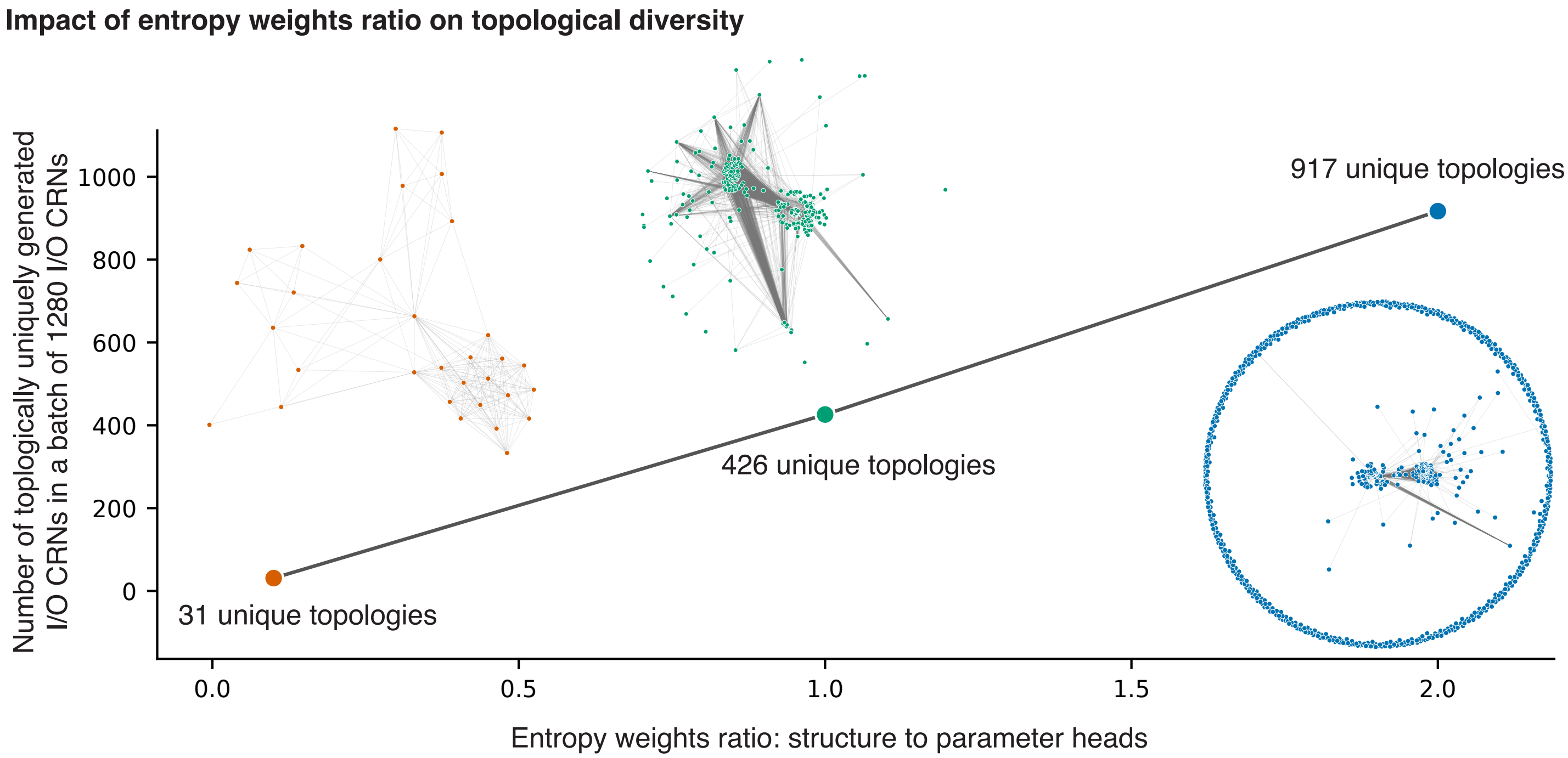

Figure S9: **Influence of the structure-to-parameter-head entropy-weight ratio on topological diversity**. The number of topologically unique I/O CRNs generated in a batch of 1,280 samples increases from 31 to 426 and 917 as the entropy-weight ratio increases from 0.1 to 1.0 and 2.0, respectively. The line connects the measured values as a visual guide. The topological diversity graph inset associated with each point shows the diversity within the corresponding batch that was generated by the trained GenAI-Net model. Each node represents a unique CRN Boolean topology signature, and edges connect topology pairs with a Hamming distance strictly smaller than three. The models were trained using the same hyperparameters as the ones in the main text with the only difference being the entropy weight of the structure head in the policy network.

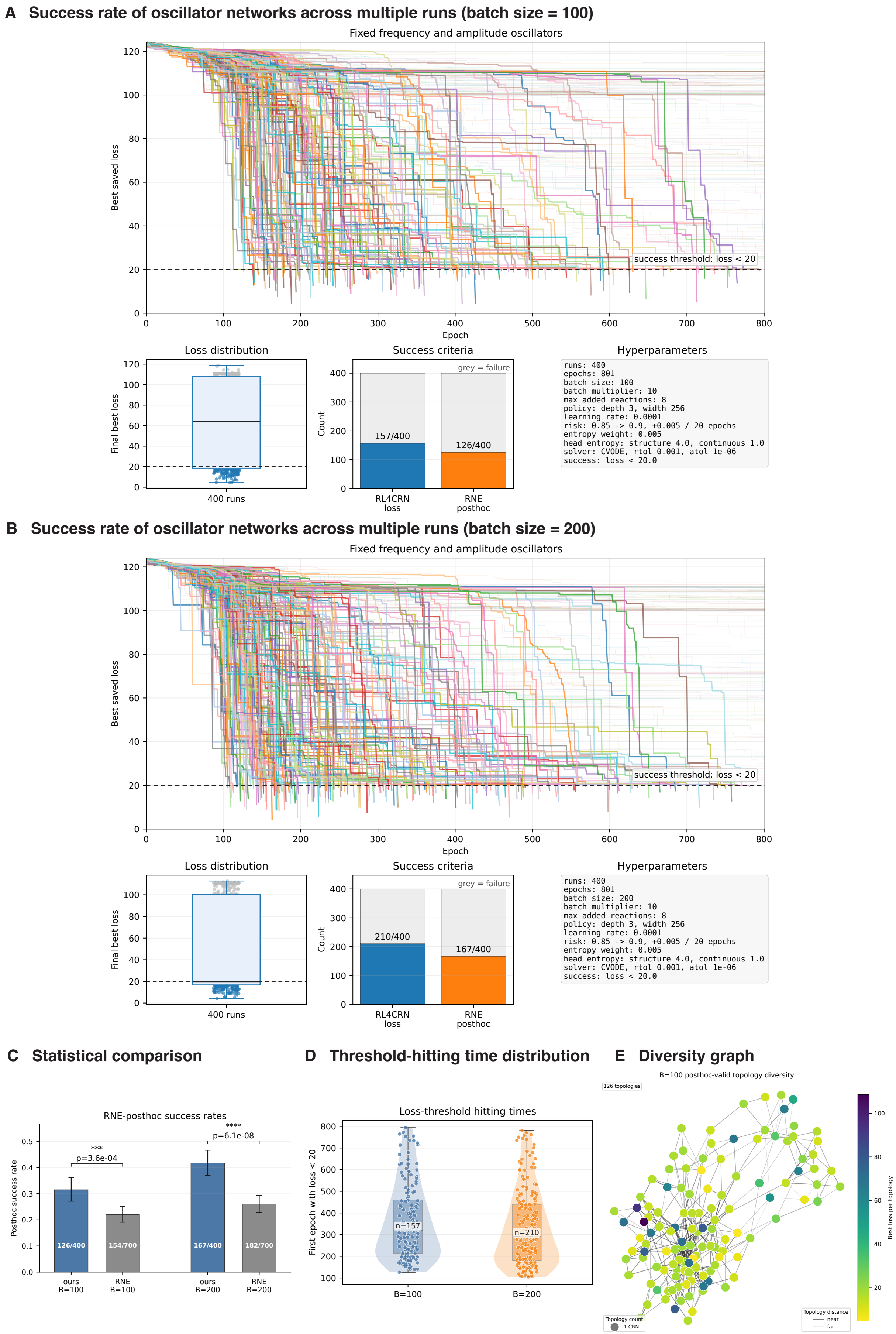

Figure S10: **Comparison of GenAI-Net with Reaction Network Evolution (RNE) under the fixed-frequency, fixed-amplitude oscillator-design setting used in the original RNE study. The figure caption is on the next page.**

Figure S10: Comparison of GenAI-Net with Reaction Network Evolution (RNE) (*54*) under the fixed-frequency, fixed-amplitude oscillator-design setting used in the original RNE study. Candidate CRNs were optimized to reproduce the nominal RNE oscillator trajectory while adding at most eight reactions. The success of a GenAI-Net run was flagged in exactly the same manner as in RNE (refer to (*54*) for details). **(a,b) Outcomes of 400 independent GenAI-Net runs using batch sizes** $B = 100$ **and** $B = 200$**, respectively.** Each run was trained for 800 epochs. Top, evolution of the best saved loss for each run; the horizontal dashed line marks the early stopping threshold $L < 20$ as in (*54*), and pale gray trajectories denote unsuccessful runs. Bottom left, distribution of the final best saved losses across all runs. Bottom center, numbers of successful runs according to two criteria: the GenAI-Net trajectory-loss threshold and the post-design oscillator-validity test used by RNE (*54*). For $B = 100$, 157 of 400 runs reached $L < 20$, and 126 passed the RNE posthoc test. For $B = 200$, 210 of 400 runs reached $L < 20$, and 167 passed the RNE posthoc test. The principal hyperparameters are listed at right. **(c) Statistical comparison of the RNE-posthoc success rates obtained by GenAI-Net with those reported in the original RNE study (*54*), matched by batch size.** GenAI-Net achieved posthoc success rates of 126/400 and 167/400 for $B = 100$ and $B = 200$, respectively, compared with the reported RNE rates of 154/700 and 182/700. Bars show empirical Bernoulli success probabilities, error bars show Wilson confidence intervals, and brackets report the p-values for the corresponding pairwise comparisons. **(d) Distribution of the first epoch at which successful GenAI-Net runs reached** $L < 20$**, shown for the 157 successful** $B = 100$ **runs and the 210 successful** $B = 200$ **runs.** Points represent individual runs and violin plots summarize their distributions. **(e) Topological diversity of the 126 CRNs generated with** $B = 100$ **that passed the RNE posthoc oscillator test.** Each node represents a distinct CRN topology; node color indicates the loss associated with that topology, node size indicates its frequency among successful runs, and edges connect topologically similar networks based on reaction-presence Hamming distance. Together, these results show that GenAI-Net achieves higher oscillator-generation success rates than those reported for RNE in their exact setting, while recovering a diverse collection of valid oscillator topologies - a feature that was not tested in (*54*).

Comparison with a fixed budget of simulations

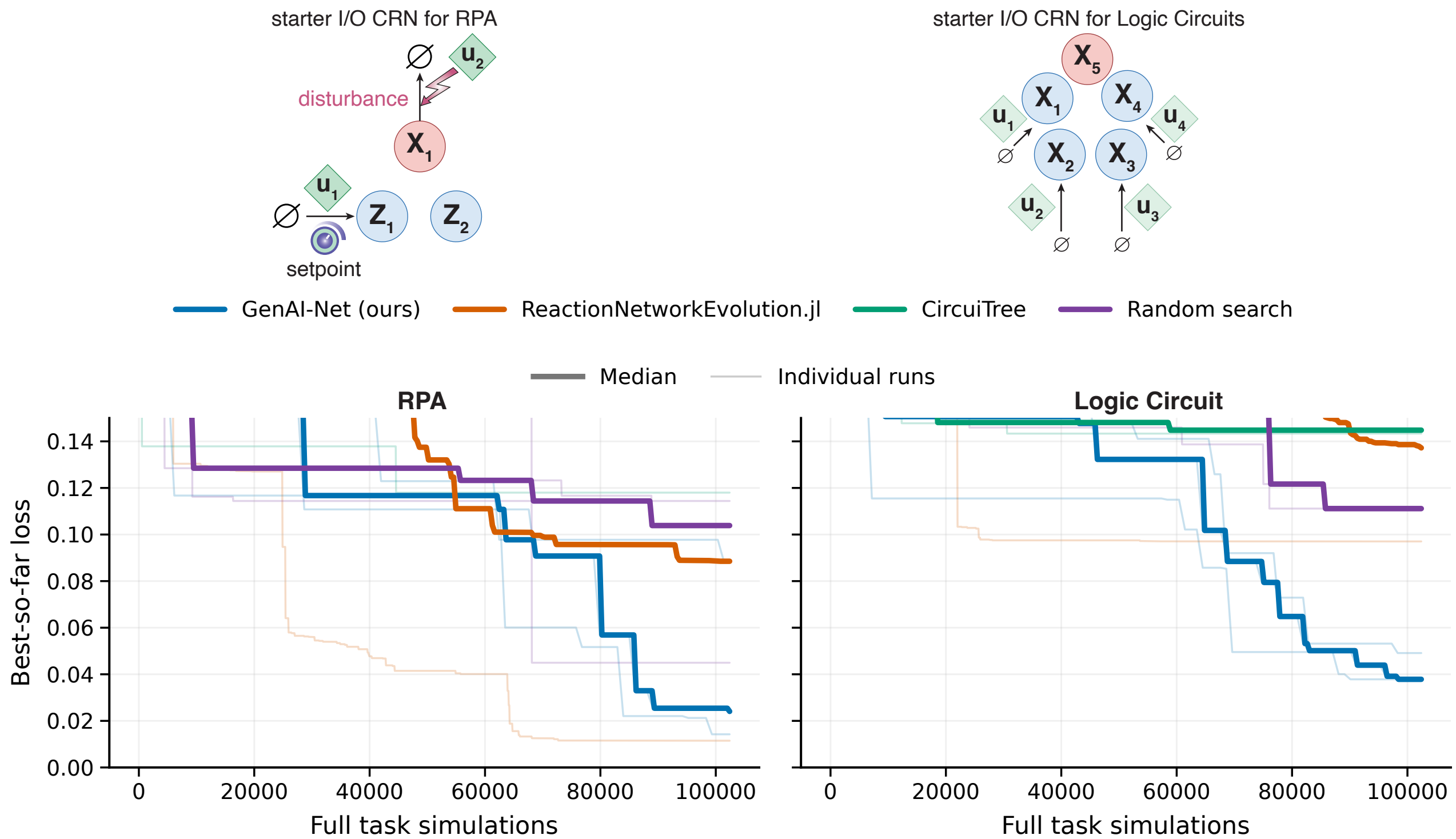

Figure S11: **Comparison of CRN-design methods under a fixed simulation budget.** GenAI-Net is compared with ReactionNetworkEvolution.jl (*54*), CircuiTree (*56*), and random search on two benchmark tasks. Top, starter I/O CRNs. For robust perfect adaptation (RPA), the input u_1 specifies the target setpoint, u_2 acts as a disturbance, and $\mathbf{X_1}$ is the regulated output; the controller species $\mathbf{Z_1}$ and $\mathbf{Z_2}$ form the starter adaptation module. For logic-circuit design, four binary inputs $u_1, \ldots, u_4$ regulate the corresponding input species $\mathbf{X_1}, \cdots, \mathbf{X_4}$, and $\mathbf{X_5}$ is the output species. All methods begin from the same task-specific starter CRN and search for additional reactions and kinetic parameters that minimize the corresponding task loss. Bottom, best-so-far loss as a function of the cumulative number of full task simulations for the RPA and logic-circuit benchmarks. A full task simulation denotes the complete evaluation of one candidate CRN across all input, disturbance, initial-condition, or truth-table conditions required by the corresponding loss. This measure accounts for differences in how the methods propose and evaluate candidates and provides a common computational-budget axis, since simulations represent the main computational bottleneck for all methods. Thin curves show independent runs, and thick curves show the median best-so-far loss across 3 runs. Lower values indicate better task performance. Across the triplicates on both benchmarks, GenAI-Net reduces the objective more rapidly and reaches lower losses within the shared simulation budget.

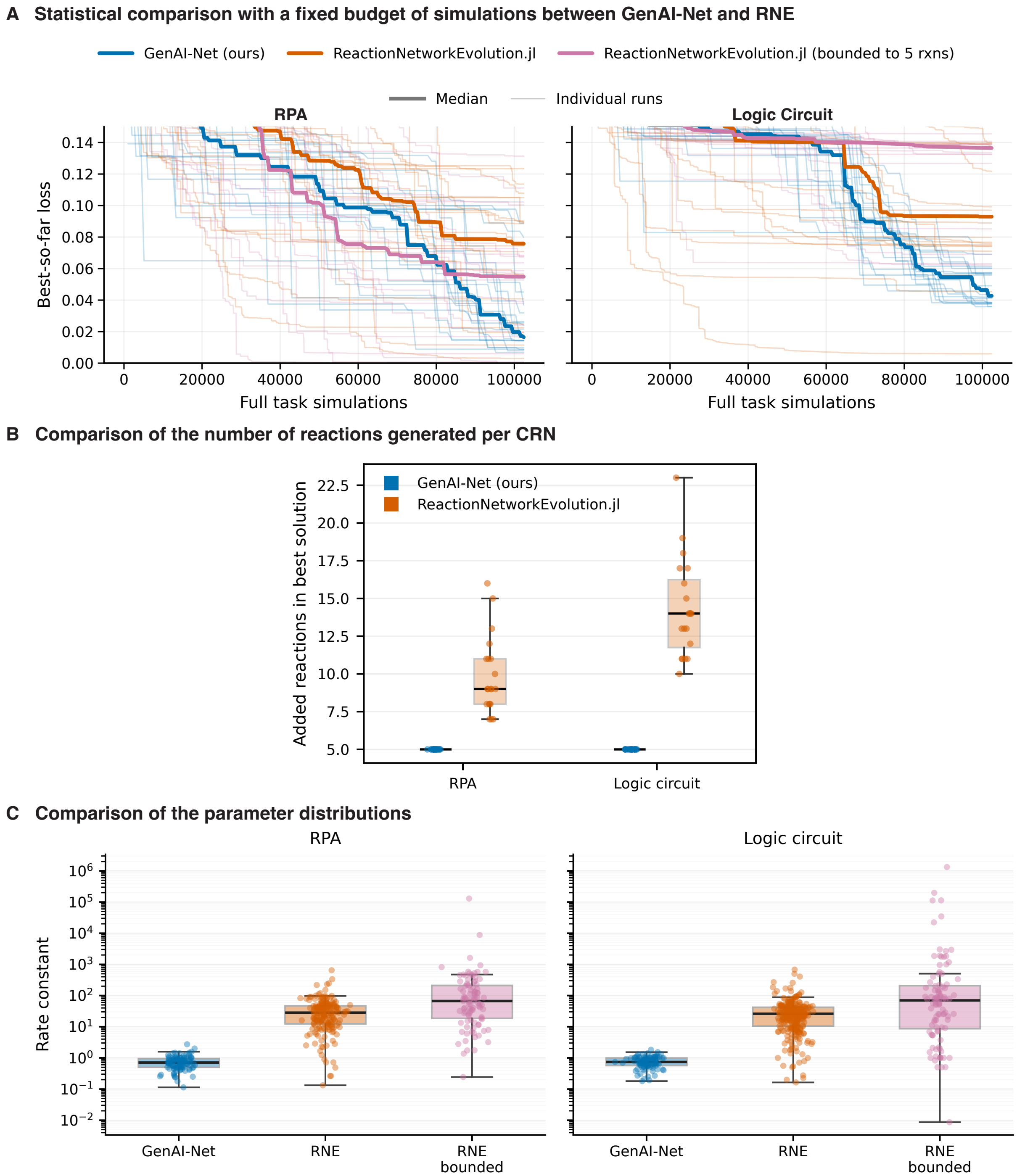

Figure S12: **Detailed comparison of GenAI-Net and ReactionNetworkEvolution.jl (RNE) (*54*) under matched simulation budgets and network-size constraints.** The methods were evaluated on the robust-perfect-adaptation (RPA) and logic-circuit benchmarks using the same task losses and starter CRNs. Standard RNE was allowed to vary the number of added reactions, whereas bounded RNE was restricted to at most five added reactions, matching the reaction budget used by GenAI-Net. **(a) Best-so-far loss as a function of the cumulative number of full task simulations.** A full task simulation denotes the complete evaluation of one candidate CRN across all conditions specified by the corresponding task loss. This provides a common computational-budget axis across methods, since simulations represent the main computational bottleneck for all methods. Thin curves show independent runs, and thick curves show the median best-so-far loss across 20 runs. Lower values indicate better task performance. GenAI-Net achieves lower median losses than standard and bounded RNE within the matched simulation budget for both tasks. Interestingly, limiting the number of reaction for the RPA task helped RNE in achieving better performances; however it was completely detrimental for the logic circuit task. This suggests that RNE struggles more in finding solutions for larger CRNs or for tasks that are not easily implementable with sub-modules in the network (e.g. integral controller sub-modules). In contrast, GenAI-Net performs consistently well in both tasks. **(b) Number of reactions added to the starter CRN in the best solution from each independent run.** Points represent individual runs, boxes summarize the corresponding distributions, and black horizontal lines indicate medians. GenAI-Net solutions contain exactly five added reactions by construction. In contrast, standard RNE frequently identifies substantially larger networks, particularly for the logic-circuit task, demonstrating that comparisons with unconstrained RNE can conflate performance with differences in permitted network complexity. **(c) Distributions of the optimized rate constants in the best CRNs generated by GenAI-Net, standard RNE, and bounded RNE.** Points represent individual rate constants pooled across the best solutions, and boxes summarize their distributions. Rate constants are shown on a logarithmic scale.

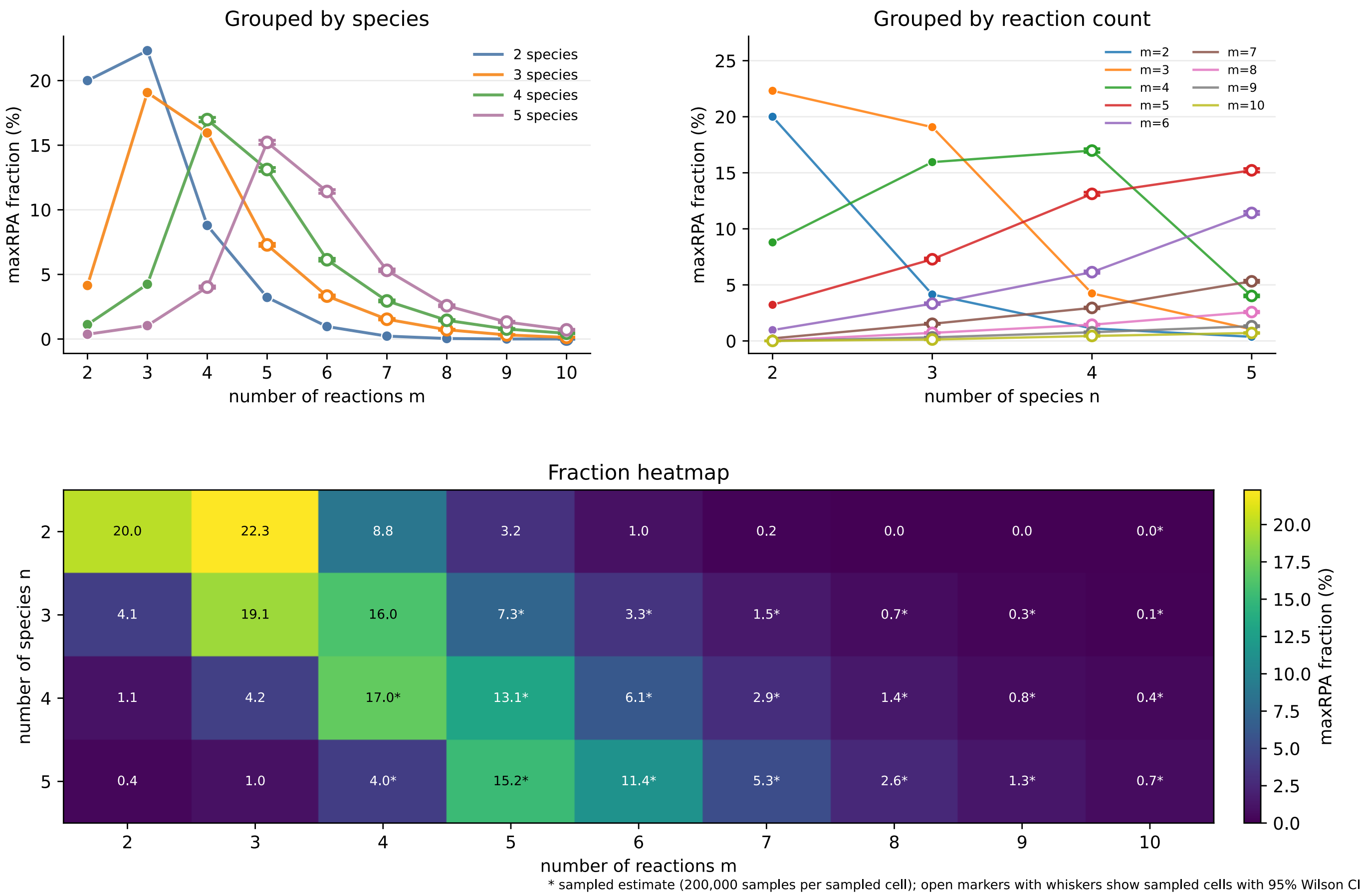

Figure S13: **Fraction of reaction networks satisfying the deterministic maxRPA (*81*) stoichiometric criterion across species and reaction counts.** For each number of labeled species $n \in \{2, 3, 4, 5\}$ and reactions $m \in \{2, \ldots, 10\}$, CRNs were drawn from the space of m-reaction subsets of all doubly bimolecular reactions, with duplicate reactions excluded and X_1 fixed as the output species. A network is counted as maxRPA if it satisfies the conditions in (*81, Section 1.4*). Top panels show the estimated (empty markers with error bars) or exact (solid markers) maxRPA fraction grouped by species count and by reaction count. Error bars denote 95% Wilson binomial confidence intervals for Monte Carlo sampled CRNs. The bottom heatmap summarizes the same fractions over the full (n, m) grid, with sampled entries indicated by asterisks and annotated with the number of Monte Carlo samples used. The non-monoticity of the solution fraction observed here aligns with the theoretical analysis carried out in the main text. Indeed, increasing the size of the search space does not necessarily reduce the solution (maxRPA) density.

Table S1: Use of AI-assisted tools during manuscript preparation and code development.

**Tool**	**Purpose**	**Own effort/check**	**Relevant sections**
ChatGPT / Codex	Revision of manuscript text for clarity, conciseness, readability, and grammar.	All suggested text edits were reviewed by the authors. Only stylistic and clarity improvements were accepted; scientific claims, interpretation, and final wording were checked and approved by the authors.	Entire manuscript
ChatGPT / Codex	Assistance in the development, debugging, documentation, and organization of code used throughout the project.	Generated or modified code was inspected, tested, and validated by the authors. Numerical results, plots, scripts, and notebooks were checked against the intended methodology before inclusion.	Code, notebooks, analysis scripts, and figures.

1 Task: Dose-Response

In this task, the networks are designed so that the steady-state concentration of the output species follows a specific functional form relative to the input u_1.

1.1 Hill-function Mapping (3 Species, 5 Reactions)

The following networks approximate a sigmoidal Hill-type response, characterized by sensitivity to a threshold concentration of the input.

I/O CRN 1

Loss: 0.01622
Inputs: $\{u_1\}$ **Species:** $\{X_1, X_2, X_3\}$ **Output:** X_3

$$\varnothing \xrightarrow{u_1} \mathrm{X}_1 \quad \textit{Rate: MAK(k=1.0, } u_1\textit{)}$$
$$\mathrm{X}_1 \xrightarrow{1.00} \varnothing \quad \textit{Rate: MAK(k=1.0)}$$
$$\mathrm{X}_1 \xrightarrow{0.55} \mathrm{X}_3 + \mathrm{X}_3 \quad \textit{Rate: MAK(k=0.5535)}$$
$$\mathrm{X}_2 \xrightarrow{1.00} \varnothing \quad \textit{Rate: MAK(k=1.0)}$$
$$\mathrm{X}_3 \xrightarrow{1.00} \varnothing \quad \textit{Rate: MAK(k=1.0)}$$
$$\mathrm{X}_3 \xrightarrow{1.65} \mathrm{X}_2 + \mathrm{X}_2 \quad \textit{Rate: MAK(k=1.6505)}$$
$$\mathrm{X}_1 + \mathrm{X}_3 \xrightarrow{1.04} \mathrm{X}_1 + \mathrm{X}_1 \quad \textit{Rate: MAK(k=1.0388)}$$
$$\mathrm{X}_2 + \mathrm{X}_2 \xrightarrow{0.71} \mathrm{X}_2 + \mathrm{X}_3 \quad \textit{Rate: MAK(k=0.7087)}$$
$$\mathrm{X}_2 + \mathrm{X}_2 \xrightarrow{1.00} \mathrm{X}_3 + \mathrm{X}_3 \quad \textit{Rate: MAK(k=1.0012)}$$

I/O CRN 4

Loss: 0.01912
Inputs: $\{u_1\}$ **Species:** $\{X_1, X_2, X_3\}$ **Output:** X_3

$$\varnothing \xrightarrow{u_1} X_1 \qquad \textit{Rate: MAK(k=1.0, } u_1\textit{)}$$
$$X_1 \xrightarrow{1.00} \varnothing \qquad \textit{Rate: MAK(k=1.0)}$$
$$X_1 \xrightarrow{0.69} X_3 + X_3 \qquad \textit{Rate: MAK(k=0.6904)}$$
$$X_2 \xrightarrow{1.00} \varnothing \qquad \textit{Rate: MAK(k=1.0)}$$
$$X_3 \xrightarrow{1.00} \varnothing \qquad \textit{Rate: MAK(k=1.0)}$$
$$X_3 \xrightarrow{1.45} X_2 + X_2 \qquad \textit{Rate: MAK(k=1.4537)}$$
$$X_1 + X_3 \xrightarrow{1.31} X_1 + X_1 \qquad \textit{Rate: MAK(k=1.3143)}$$
$$X_2 + X_2 \xrightarrow{1.37} X_2 + X_3 \qquad \textit{Rate: MAK(k=1.3718)}$$
$$X_2 + X_3 \xrightarrow{1.22} X_3 + X_3 \qquad \textit{Rate: MAK(k=1.2243)}$$

I/O CRN 8

Loss: 0.02026
Inputs: $\{u_1\}$ **Species:** $\{X_1, X_2, X_3\}$ **Output:** X_3

$$\varnothing \xrightarrow{u_1} X_1 \qquad \textit{Rate: MAK(k=1.0, } u_1\textit{)}$$
$$X_1 \xrightarrow{1.00} \varnothing \qquad \textit{Rate: MAK(k=1.0)}$$
$$X_1 \xrightarrow{1.38} X_3 \qquad \textit{Rate: MAK(k=1.3840)}$$
$$X_2 \xrightarrow{1.00} \varnothing \qquad \textit{Rate: MAK(k=1.0)}$$
$$X_3 \xrightarrow{1.00} \varnothing \qquad \textit{Rate: MAK(k=1.0)}$$
$$X_3 \xrightarrow{1.34} X_1 + X_2 \qquad \textit{Rate: MAK(k=1.3439)}$$
$$X_3 \xrightarrow{1.27} X_2 + X_3 \qquad \textit{Rate: MAK(k=1.2653)}$$
$$X_1 + X_2 \xrightarrow{1.53} X_1 + X_3 \qquad \textit{Rate: MAK(k=1.5331)}$$
$$X_1 + X_3 \xrightarrow{1.33} X_1 + X_1 \qquad \textit{Rate: MAK(k=1.3333)}$$

1.2 Ultra-Sensitive Mapping (3 Species, 5 Reactions)

This subsection details the networks optimized for Ultra-Sensitive responses, characterized by a very steep "switch-like" transition in output concentration over a narrow range of input values. These are typically achieved through mechanisms like zero-order ultrasensitivity or molecular titration.

I/O CRN 1

Loss: 0.01380
Inputs: $\{u_1\}$ **Species:** $\{X_1, X_2, X_3\}$ **Output:** X_3

$$\varnothing \xrightarrow{u_1} \mathrm{X}_1 \qquad \textit{Rate: MAK(k=1.0, } u_1\textit{)}$$
$$\mathrm{X}_1 \xrightarrow{1.00} \varnothing \qquad \textit{Rate: MAK(k=1.0)}$$
$$\mathrm{X}_1 \xrightarrow{0.79} \mathrm{X}_3 + \mathrm{X}_3 \qquad \textit{Rate: MAK(k=0.7918)}$$
$$\mathrm{X}_2 \xrightarrow{1.00} \varnothing \qquad \textit{Rate: MAK(k=1.0)}$$
$$\mathrm{X}_3 \xrightarrow{1.00} \varnothing \qquad \textit{Rate: MAK(k=1.0)}$$
$$\mathrm{X}_3 \xrightarrow{34.96} \mathrm{X}_2 + \mathrm{X}_2 \qquad \textit{Rate: MAK(k=34.9569)}$$
$$\mathrm{X}_1 + \mathrm{X}_2 \xrightarrow{1.78} \mathrm{X}_1 + \mathrm{X}_3 \qquad \textit{Rate: MAK(k=1.7771)}$$
$$\mathrm{X}_1 + \mathrm{X}_3 \xrightarrow{1.78} \mathrm{X}_1 + \mathrm{X}_1 \qquad \textit{Rate: MAK(k=1.7797)}$$
$$\mathrm{X}_2 + \mathrm{X}_3 \xrightarrow{1.82} \mathrm{X}_1 + \mathrm{X}_2 \qquad \textit{Rate: MAK(k=1.8197)}$$

I/O CRN 20

Loss: 0.02082
Inputs: $\{u_1\}$ **Species:** $\{X_1, X_2, X_3\}$ **Output:** X_3

$$\varnothing \xrightarrow{u_1} \mathrm{X}_1 \qquad \textit{Rate: MAK(k=1.0, } u_1\textit{)}$$
$$\mathrm{X}_1 \xrightarrow{1.00} \varnothing \qquad \textit{Rate: MAK(k=1.0)}$$
$$\mathrm{X}_1 \xrightarrow{12.07} \mathrm{X}_3 + \mathrm{X}_3 \qquad \textit{Rate: MAK(k=12.0656)}$$
$$\mathrm{X}_2 \xrightarrow{1.00} \varnothing \qquad \textit{Rate: MAK(k=1.0)}$$
$$\mathrm{X}_3 \xrightarrow{1.00} \varnothing \qquad \textit{Rate: MAK(k=1.0)}$$
$$\mathrm{X}_3 \xrightarrow{4.97} \mathrm{X}_2 \qquad \textit{Rate: MAK(k=4.9710)}$$
$$\mathrm{X}_3 \xrightarrow{16.52} \mathrm{X}_2 + \mathrm{X}_2 \qquad \textit{Rate: MAK(k=16.5216)}$$
$$\mathrm{X}_1 + \mathrm{X}_1 \xrightarrow{4.21} \mathrm{X}_1 + \mathrm{X}_2 \qquad \textit{Rate: MAK(k=4.2149)}$$
$$\mathrm{X}_2 + \mathrm{X}_3 \xrightarrow{1.53} \mathrm{X}_1 + \mathrm{X}_1 \qquad \textit{Rate: MAK(k=1.5290)}$$

1.3 Biphasic Mapping (3 Species, 5 Reactions)

This subsection presents networks that exhibit a Biphasic (or bell-shaped) response. In these systems, the output concentration initially increases with the input stimulus u_1, but subsequently decreases as the input concentration continues to rise.

I/O CRN 1

Loss: 0.05380
Inputs: $\{u_1\}$ **Species:** $\{X_1, X_2, X_3\}$ **Output:** X_3

$$\varnothing \xrightarrow{u_1} \mathrm{X_1} \qquad \textit{Rate: MAK(k=1.0, } u_1\textit{)}$$
$$\mathrm{X_1} \xrightarrow{1.00} \varnothing \qquad \textit{Rate: MAK(k=1.0)}$$
$$\mathrm{X_2} \xrightarrow{1.00} \varnothing \qquad \textit{Rate: MAK(k=1.0)}$$
$$\mathrm{X_2} \xrightarrow{0.74} \mathrm{X_1} + \mathrm{X_2} \qquad \textit{Rate: MAK(k=0.7361)}$$
$$\mathrm{X_3} \xrightarrow{1.00} \varnothing \qquad \textit{Rate: MAK(k=1.0)}$$
$$\mathrm{X_3} \xrightarrow{0.57} \mathrm{X_1} + \mathrm{X_2} \qquad \textit{Rate: MAK(k=0.5742)}$$
$$\mathrm{X_1} + \mathrm{X_1} \xrightarrow{0.44} \mathrm{X_3} + \mathrm{X_3} \qquad \textit{Rate: MAK(k=0.4442)}$$
$$\mathrm{X_1} + \mathrm{X_2} \xrightarrow{0.39} \mathrm{X_2} + \mathrm{X_2} \qquad \textit{Rate: MAK(k=0.3872)}$$
$$\mathrm{X_2} + \mathrm{X_3} \xrightarrow{0.70} \mathrm{X_1} + \mathrm{X_2} \qquad \textit{Rate: MAK(k=0.6972)}$$

I/O CRN 12

Loss: 0.06556
Inputs: $\{u_1\}$ **Species:** $\{X_1, X_2, X_3\}$ **Output:** X_3

$$\varnothing \xrightarrow{u_1} \mathrm{X_1} \qquad \textit{Rate: MAK(k=1.0, } u_1\textit{)}$$
$$\mathrm{X_1} \xrightarrow{1.00} \varnothing \qquad \textit{Rate: MAK(k=1.0)}$$
$$\mathrm{X_2} \xrightarrow{1.00} \varnothing \qquad \textit{Rate: MAK(k=1.0)}$$
$$\mathrm{X_3} \xrightarrow{1.00} \varnothing \qquad \textit{Rate: MAK(k=1.0)}$$
$$\mathrm{X_1} + \mathrm{X_1} \xrightarrow{0.57} \mathrm{X_3} + \mathrm{X_3} \qquad \textit{Rate: MAK(k=0.5680)}$$
$$\mathrm{X_1} + \mathrm{X_2} \xrightarrow{0.79} \mathrm{X_2} + \mathrm{X_2} \qquad \textit{Rate: MAK(k=0.7884)}$$
$$\mathrm{X_2} + \mathrm{X_3} \xrightarrow{0.37} \mathrm{X_1} + \mathrm{X_1} \qquad \textit{Rate: MAK(k=0.3690)}$$
$$\mathrm{X_2} + \mathrm{X_3} \xrightarrow{0.52} \mathrm{X_1} + \mathrm{X_2} \qquad \textit{Rate: MAK(k=0.5194)}$$
$$\mathrm{X_3} + \mathrm{X_3} \xrightarrow{0.29} \mathrm{X_2} + \mathrm{X_3} \qquad \textit{Rate: MAK(k=0.2924)}$$

2 Task: Robust Perfect Adaptation

This section details the In-Silico Optimized Chemical Reaction Networks (I/O CRNs) discovered for the Robust Perfect Adaptation task. Reaction rates (rounded to 2 decimal places) are displayed above the arrows, with full parameter details provided on the right.

2.1 Configuration: 3 Species, 5 Reactions

I/O CRN 1

Loss: 0.00292
Inputs: $\{u_1, u_2\}$ **Species:** $\{X_1, Z_1, Z_2\}$ **Output:** X_1

$$\varnothing \xrightarrow{u_1} \mathrm{Z_1} \qquad \textit{Rate: MAK(k=1.0, } u_1\textit{)}$$
$$\varnothing \xrightarrow{0.95} \mathrm{X_1} + \mathrm{X_1} \qquad \textit{Rate: MAK(k=0.9523)}$$
$$\mathrm{X_1} \xrightarrow{u_2} \varnothing \qquad \textit{Rate: MAK(k=1.0, } u_2\textit{)}$$
$$\mathrm{X_1} \xrightarrow{1.00} \mathrm{Z_2} \qquad \textit{Rate: MAK(k=1.0007)}$$
$$\mathrm{Z_1} \xrightarrow{3.11} \mathrm{X_1} + \mathrm{Z_1} \qquad \textit{Rate: MAK(k=3.1131)}$$
$$\mathrm{X_1} + \mathrm{Z_2} \xrightarrow{2.85} \mathrm{Z_2} \qquad \textit{Rate: MAK(k=2.8472)}$$
$$\mathrm{Z_1} + \mathrm{Z_2} \xrightarrow{1.03} \varnothing \qquad \textit{Rate: MAK(k=1.0346)}$$

I/O CRN 19

Loss: 0.00519
Inputs: $\{u_1, u_2\}$ **Species:** $\{X_1, Z_1, Z_2\}$ **Output:** X_1

$$\varnothing \xrightarrow{u_1} \mathrm{Z_1} \qquad \textit{Rate: MAK(k=1.0, } u_1\textit{)}$$
$$\mathrm{X_1} \xrightarrow{u_2} \varnothing \qquad \textit{Rate: MAK(k=1.0, } u_2\textit{)}$$
$$\mathrm{X_1} \xrightarrow{1.00} \mathrm{Z_2} \qquad \textit{Rate: MAK(k=1.0007)}$$
$$\mathrm{Z_1} \xrightarrow{1.62} \mathrm{X_1} + \mathrm{Z_1} \qquad \textit{Rate: MAK(k=1.6172)}$$
$$\mathrm{X_1} + \mathrm{X_1} \xrightarrow{1.01} \varnothing \qquad \textit{Rate: MAK(k=1.0056)}$$
$$\mathrm{X_1} + \mathrm{Z_2} \xrightarrow{1.02} \mathrm{Z_2} \qquad \textit{Rate: MAK(k=1.0227)}$$
$$\mathrm{Z_1} + \mathrm{Z_2} \xrightarrow{1.68} \mathrm{X_1} + \mathrm{X_1} \qquad \textit{Rate: MAK(k=1.6823)}$$

I/O CRN 28

Loss: 0.00584
Inputs: $\{u_1, u_2\}$ **Species:** $\{X_1, Z_1, Z_2\}$ **Output:** X_1

$$\varnothing \xrightarrow{u_1} \mathrm{Z_1} \qquad \textit{Rate: MAK(k=1.0, } u_1\textit{)}$$
$$\mathrm{X_1} \xrightarrow{u_2} \varnothing \qquad \textit{Rate: MAK(k=1.0, } u_2\textit{)}$$
$$\mathrm{X_1} \xrightarrow{1.00} \mathrm{Z_2} \qquad \textit{Rate: MAK(k=1.0007)}$$
$$\mathrm{X_1} \xrightarrow{1.24} \mathrm{X_1} + \mathrm{X_1} \qquad \textit{Rate: MAK(k=1.2353)}$$
$$\mathrm{Z_1} \xrightarrow{3.14} \mathrm{X_1} + \mathrm{Z_1} \qquad \textit{Rate: MAK(k=3.1393)}$$
$$\mathrm{X_1} + \mathrm{X_1} \xrightarrow{1.01} \mathrm{Z_1} + \mathrm{Z_2} \qquad \textit{Rate: MAK(k=1.0059)}$$
$$\mathrm{Z_1} + \mathrm{Z_2} \xrightarrow{1.40} \varnothing \qquad \textit{Rate: MAK(k=1.4007)}$$

2.2 Configuration: 6 Species, 8 Reactions

I/O CRN 1

Loss: 0.00480
Inputs: $\{u_1, u_2, u_3\}$ **Species:** $\{X_1, X_2, Z_1, Z_2, Z_3, Z_4\}$ **Output:** X_2

$$\varnothing \xrightarrow{u_1} \mathrm{Z}_1 \qquad \textit{Rate: MAK(k=1.0, } u_1\textit{)}$$
$$\mathrm{X}_1 \xrightarrow{u_2} \varnothing \qquad \textit{Rate: MAK(k=1.0, } u_2\textit{)}$$
$$\mathrm{X}_1 \xrightarrow{1.00} \mathrm{X}_1 + \mathrm{X}_2 \qquad \textit{Rate: MAK(k=1.0)}$$
$$\mathrm{X}_2 \xrightarrow{u_3} \varnothing \qquad \textit{Rate: MAK(k=1.0, } u_3\textit{)}$$
$$\mathrm{X}_2 \xrightarrow{1.77} \mathrm{X}_2 + \mathrm{Z}_3 \qquad \textit{Rate: MAK(k=1.7734)}$$
$$\mathrm{X}_2 \xrightarrow{0.50} \mathrm{Z}_4 + \mathrm{Z}_4 \qquad \textit{Rate: MAK(k=0.4996)}$$
$$\mathrm{Z}_1 \xrightarrow{5.64} \mathrm{X}_1 + \mathrm{Z}_1 \qquad \textit{Rate: MAK(k=5.6404)}$$
$$\mathrm{X}_1 + \mathrm{X}_2 \xrightarrow{1.45} \mathrm{X}_2 \qquad \textit{Rate: MAK(k=1.4502)}$$
$$\mathrm{X}_1 + \mathrm{Z}_4 \xrightarrow{3.66} \mathrm{Z}_3 + \mathrm{Z}_4 \qquad \textit{Rate: MAK(k=3.6597)}$$
$$\mathrm{Z}_1 + \mathrm{Z}_4 \xrightarrow{1.72} \varnothing \qquad \textit{Rate: MAK(k=1.7200)}$$
$$\mathrm{Z}_1 + \mathrm{Z}_4 \xrightarrow{1.32} \mathrm{Z}_3 \qquad \textit{Rate: MAK(k=1.3229)}$$
$$\mathrm{Z}_2 + \mathrm{Z}_3 \xrightarrow{5.59} \mathrm{Z}_2 + \mathrm{Z}_2 \qquad \textit{Rate: MAK(k=5.5870)}$$

I/O CRN 14

Loss: 0.00567
Inputs: $\{u_1, u_2, u_3\}$ **Species:** $\{X_1, X_2, Z_1, Z_3, Z_4\}$ **Output:** X_2

$$\varnothing \xrightarrow{u_1} \mathrm{Z}_1 \qquad \textit{Rate: MAK(k=1.0, } u_1\textit{)}$$
$$\mathrm{X}_1 \xrightarrow{u_2} \varnothing \qquad \textit{Rate: MAK(k=1.0, } u_2\textit{)}$$
$$\mathrm{X}_1 \xrightarrow{1.00} \mathrm{X}_1 + \mathrm{X}_2 \qquad \textit{Rate: MAK(k=1.0)}$$
$$\mathrm{X}_2 \xrightarrow{u_3} \varnothing \qquad \textit{Rate: MAK(k=1.0, } u_3\textit{)}$$
$$\mathrm{X}_2 \xrightarrow{0.67} \mathrm{X}_2 + \mathrm{Z}_3 \qquad \textit{Rate: MAK(k=0.6670)}$$
$$\mathrm{X}_2 \xrightarrow{0.50} \mathrm{Z}_4 + \mathrm{Z}_4 \qquad \textit{Rate: MAK(k=0.5001)}$$
$$\mathrm{Z}_1 \xrightarrow{3.07} \mathrm{X}_1 + \mathrm{Z}_1 \qquad \textit{Rate: MAK(k=3.0747)}$$
$$\mathrm{X}_1 + \mathrm{X}_2 \xrightarrow{0.82} \mathrm{X}_2 \qquad \textit{Rate: MAK(k=0.8197)}$$
$$\mathrm{X}_2 + \mathrm{Z}_4 \xrightarrow{0.51} \mathrm{Z}_3 + \mathrm{Z}_4 \qquad \textit{Rate: MAK(k=0.5080)}$$
$$\mathrm{Z}_1 + \mathrm{Z}_4 \xrightarrow{0.36} \varnothing \qquad \textit{Rate: MAK(k=0.3640)}$$
$$\mathrm{Z}_1 + \mathrm{Z}_4 \xrightarrow{0.68} \mathrm{Z}_3 \qquad \textit{Rate: MAK(k=0.6823)}$$
$$\mathrm{Z}_1 + \mathrm{Z}_4 \xrightarrow{0.49} \mathrm{Z}_3 + \mathrm{Z}_3 \qquad \textit{Rate: MAK(k=0.4917)}$$

I/O CRN 27

Loss: 0.00611
Inputs: $\{u_1, u_2, u_3\}$ **Species:** $\{X_1, X_2, Z_1, Z_2, Z_3, Z_4\}$ **Output:** X_2

$\varnothing \xrightarrow{u_1} \mathrm{Z_1}$	*Rate: MAK(k=1.0, u_1)*
$\mathrm{X_1} \xrightarrow{u_2} \varnothing$	*Rate: MAK(k=1.0, u_2)*
$\mathrm{X_1} \xrightarrow{1.00} \mathrm{X_1 + X_2}$	*Rate: MAK(k=1.0)*
$\mathrm{X_2} \xrightarrow{u_3} \varnothing$	*Rate: MAK(k=1.0, u_3)*
$\mathrm{X_2} \xrightarrow{0.50} \mathrm{Z_4 + Z_4}$	*Rate: MAK(k=0.5004)*
$\mathrm{Z_1} \xrightarrow{1.80} \mathrm{X_1 + Z_1}$	*Rate: MAK(k=1.8020)*
$\mathrm{X_1 + Z_4} \xrightarrow{0.48} \mathrm{Z_4}$	*Rate: MAK(k=0.4796)*
$\mathrm{X_1 + Z_4} \xrightarrow{0.41} \mathrm{Z_3 + Z_4}$	*Rate: MAK(k=0.4090)*
$\mathrm{X_2 + Z_4} \xrightarrow{1.38} \mathrm{Z_3 + Z_4}$	*Rate: MAK(k=1.3803)*
$\mathrm{Z_1 + Z_4} \xrightarrow{0.51} \mathrm{Z_3 + Z_3}$	*Rate: MAK(k=0.5082)*
$\mathrm{Z_2 + Z_2} \xrightarrow{0.57} \mathrm{Z_2}$	*Rate: MAK(k=0.5725)*
$\mathrm{Z_3 + Z_3} \xrightarrow{0.49} \mathrm{Z_2}$	*Rate: MAK(k=0.4938)*

2.3 Configuration: 2 Species, 4 Reactions

I/O CRN 2

Loss: 0.01230
Inputs: $\{u_1, u_2\}$ **Species:** $\{X, Z\}$ **Output:** X

$$\varnothing \xrightarrow{1.09} \varnothing \quad \textit{Rate: MAK(k=1.0875)}$$
$$\varnothing \xrightarrow{1.13} \mathrm{X} + \mathrm{X} \quad \textit{Rate: MAK(k=1.1297)}$$
$$\mathrm{X} \xrightarrow{u_2} \varnothing \quad \textit{Rate: MAK(k=1.0, } u_2\textit{)}$$
$$\mathrm{Z} \xrightarrow{u_1} \varnothing \quad \textit{Rate: MAK(k=1.0, } u_1\textit{)}$$
$$\mathrm{X} + \mathrm{Z} \xrightarrow{1.08} \mathrm{Z} \quad \textit{Rate: MAK(k=1.0831)}$$
$$\mathrm{X} + \mathrm{Z} \xrightarrow{1.00} \mathrm{Z} + \mathrm{Z} \quad \textit{Rate: MAK(k=0.9993)}$$

I/O CRN 4

Loss: 0.01494
Inputs: $\{u_1, u_2\}$ **Species:** $\{X, Z\}$ **Output:** X

$$\varnothing \xrightarrow{0.91} \varnothing \quad \textit{Rate: MAK(k=0.9145)}$$
$$\varnothing \xrightarrow{0.89} \mathrm{X} \quad \textit{Rate: MAK(k=0.8927)}$$
$$\varnothing \xrightarrow{0.89} \mathrm{X} + \mathrm{X} \quad \textit{Rate: MAK(k=0.8912)}$$
$$\mathrm{X} \xrightarrow{u_2} \varnothing \quad \textit{Rate: MAK(k=1.0, } u_2\textit{)}$$
$$\mathrm{Z} \xrightarrow{u_1} \varnothing \quad \textit{Rate: MAK(k=1.0, } u_1\textit{)}$$
$$\mathrm{X} + \mathrm{Z} \xrightarrow{1.00} \mathrm{Z} + \mathrm{Z} \quad \textit{Rate: MAK(k=0.9999)}$$

I/O CRN 9

Loss: 0.02902
Inputs: $\{u_1, u_2\}$ **Species:** $\{X, Z\}$ **Output:** X

$$\varnothing \xrightarrow{1.09} \mathrm{X} + \mathrm{X} \quad \textit{Rate: MAK(k=1.0885)}$$
$$\mathrm{X} \xrightarrow{u_2} \varnothing \quad \textit{Rate: MAK(k=1.0, } u_2\textit{)}$$
$$\mathrm{Z} \xrightarrow{u_1} \varnothing \quad \textit{Rate: MAK(k=1.0, } u_1\textit{)}$$
$$\mathrm{Z} \xrightarrow{1.00} \mathrm{X} + \mathrm{Z} \quad \textit{Rate: MAK(k=0.9996)}$$
$$\mathrm{X} + \mathrm{Z} \xrightarrow{1.05} \mathrm{Z} \quad \textit{Rate: MAK(k=1.0454)}$$
$$\mathrm{X} + \mathrm{Z} \xrightarrow{1.01} \mathrm{Z} + \mathrm{Z} \quad \textit{Rate: MAK(k=1.0089)}$$

2.4 Configuration: 2 Species, 6 Reactions

I/O CRN 1

Loss: 0.00059
Inputs: $\{u_1\}$ **Species:** $\{X_1, X_2\}$ **Output:** $\{X_1, X_2\}$

$$\varnothing \xrightarrow{2.15} \varnothing$$ *Rate: MAK(k=2.1519)*

$$X_1 \xrightarrow{u_1} \varnothing$$ *Rate: MAK(k=1.0, u_1)*

$$X_1 \xrightarrow{5.10} X_1 + X_1$$ *Rate: MAK(k=5.1015)*

$$X_2 \xrightarrow{1.00} \varnothing$$ *Rate: MAK(k=1.0)*

$$X_2 \xrightarrow{5.61} X_2 + X_2$$ *Rate: MAK(k=5.6112)*

$$X_1 + X_1 \xrightarrow{2.05} \varnothing$$ *Rate: MAK(k=2.0498)*

$$X_1 + X_2 \xrightarrow{15.79} \varnothing$$ *Rate: MAK(k=15.7926)*

$$X_2 + X_2 \xrightarrow{2.30} \varnothing$$ *Rate: MAK(k=2.3049)*

I/O CRN 11

Loss: 0.00193
Inputs: $\{u_1\}$ **Species:** $\{X_1, X_2\}$ **Output:** $\{X_1, X_2\}$

$$\varnothing \xrightarrow{1.57} \varnothing$$ *Rate: MAK(k=1.5695)*

$$X_1 \xrightarrow{u_1} \varnothing$$ *Rate: MAK(k=1.0, u_1)*

$$X_1 \xrightarrow{2.56} X_1 + X_1$$ *Rate: MAK(k=2.5650)*

$$X_2 \xrightarrow{1.00} \varnothing$$ *Rate: MAK(k=1.0)*

$$X_2 \xrightarrow{2.67} X_2 + X_2$$ *Rate: MAK(k=2.6720)*

$$X_1 + X_1 \xrightarrow{1.56} X_1$$ *Rate: MAK(k=1.5628)*

$$X_1 + X_2 \xrightarrow{4.01} \varnothing$$ *Rate: MAK(k=4.0056)*

$$X_2 + X_2 \xrightarrow{1.67} X_2$$ *Rate: MAK(k=1.6697)*

I/O CRN 14

Loss: 0.00197
Inputs: $\{u_1\}$ **Species:** $\{X_1, X_2\}$ **Output:** $\{X_1, X_2\}$

$$X_1 \xrightarrow{u_1} \varnothing$$ *Rate: MAK(k=1.0, u_1)*

$$X_1 \xrightarrow{3.73} X_1 + X_1$$ *Rate: MAK(k=3.7344)*

$$X_2 \xrightarrow{1.00} \varnothing$$ *Rate: MAK(k=1.0)*

$$X_2 \xrightarrow{4.34} X_2 + X_2$$ *Rate: MAK(k=4.3372)*

$$X_1 + X_1 \xrightarrow{1.37} \varnothing$$ *Rate: MAK(k=1.3690)*

$$X_1 + X_2 \xrightarrow{13.15} \varnothing$$ *Rate: MAK(k=13.1543)*

$$X_1 + X_2 \xrightarrow{1.54} X_1$$ *Rate: MAK(k=1.5375)*

$$X_2 + X_2 \xrightarrow{1.66} \varnothing$$ *Rate: MAK(k=1.6614)*

3 Task: Robust Perfect Adaptation with CV Reduction

This section presents I/O CRNs optimized with stochastic simulations. These networks are designed to achieve Robust Perfect Adaptation of the mean output X_1 while suppressing stochastic fluctuations (reducing the Coefficient of Variation).

3.1 Configuration: 4 Species, 8 Reactions

I/O CRN 1

Loss: 0.20004
Inputs: $\{u_1, u_2\}$ **Species:** $\{X_1, Z_1, Z_2, Z_3\}$ **Output:** X_1

$\varnothing \xrightarrow{3\,u_1} \mathrm{Z_1}$	*Rate: MAK(k=3.0, u_1)*
$\mathrm{X_1} \xrightarrow{u_2} \varnothing$	*Rate: MAK(k=1.0, u_2)*
$\mathrm{X_1} \xrightarrow{0.99} \mathrm{Z_3}$	*Rate: MAK(k=0.9901)*
$\mathrm{Z_1} \xrightarrow{0.96} \mathrm{X_1} + \mathrm{Z_1}$	*Rate: MAK(k=0.9588)*
$\mathrm{X_1} + \mathrm{X_1} \xrightarrow{1.11} \mathrm{X_1}$	*Rate: MAK(k=1.1058)*
$\mathrm{X_1} + \mathrm{X_1} \xrightarrow{1.74} \mathrm{X_1} + \mathrm{Z_2}$	*Rate: MAK(k=1.7382)*
$\mathrm{Z_1} + \mathrm{Z_3} \xrightarrow{1.17} \mathrm{X_1}$	*Rate: MAK(k=1.1736)*
$\mathrm{Z_2} + \mathrm{Z_2} \xrightarrow{1.56} \mathrm{Z_1} + \mathrm{Z_3}$	*Rate: MAK(k=1.5645)*

I/O CRN 31

Loss: 0.20772
Inputs: $\{u_1, u_2\}$ **Species:** $\{X_1, Z_1, Z_2, Z_3\}$ **Output:** X_1

$\varnothing \xrightarrow{3\,u_1} \mathrm{Z_1}$	*Rate: MAK(k=3.0, u_1)*
$\mathrm{X_1} \xrightarrow{u_2} \varnothing$	*Rate: MAK(k=1.0, u_2)*
$\mathrm{X_1} \xrightarrow{1.00} \mathrm{Z_3}$	*Rate: MAK(k=0.9958)*
$\mathrm{Z_1} \xrightarrow{1.14} \mathrm{X_1} + \mathrm{Z_1}$	*Rate: MAK(k=1.1427)*
$\mathrm{Z_2} \xrightarrow{1.37} \varnothing$	*Rate: MAK(k=1.3746)*
$\mathrm{X_1} + \mathrm{X_1} \xrightarrow{1.48} \mathrm{X_1}$	*Rate: MAK(k=1.4754)*
$\mathrm{Z_1} + \mathrm{Z_3} \xrightarrow{1.04} \mathrm{X_1}$	*Rate: MAK(k=1.0382)*
$\mathrm{Z_2} + \mathrm{Z_3} \xrightarrow{0.80} \mathrm{Z_1} + \mathrm{Z_3}$	*Rate: MAK(k=0.7966)*

I/O CRN 34

Loss: 0.20897
Inputs: $\{u_1, u_2\}$ **Species:** $\{X_1, Z_1, Z_2, Z_3\}$ **Output:** X_1

$\varnothing \xrightarrow{3\,u_1} \mathrm{Z}_1$	*Rate: MAK(k=3.0, u_1)*
$\mathrm{X}_1 \xrightarrow{u_2} \varnothing$	*Rate: MAK(k=1.0, u_2)*
$\mathrm{X}_1 \xrightarrow{0.50} \mathrm{Z}_2 + \mathrm{Z}_2$	*Rate: MAK(k=0.4972)*
$\mathrm{Z}_1 \xrightarrow{1.18} \mathrm{X}_1 + \mathrm{Z}_1$	*Rate: MAK(k=1.1756)*
$\mathrm{X}_1 + \mathrm{X}_1 \xrightarrow{2.28} \mathrm{X}_1$	*Rate: MAK(k=2.2788)*
$\mathrm{Z}_1 + \mathrm{Z}_2 \xrightarrow{0.45} \varnothing$	*Rate: MAK(k=0.4548)*
$\mathrm{Z}_1 + \mathrm{Z}_3 \xrightarrow{0.74} \mathrm{Z}_2 + \mathrm{Z}_2$	*Rate: MAK(k=0.7377)*
$\mathrm{Z}_2 + \mathrm{Z}_3 \xrightarrow{0.68} \mathrm{X}_1 + \mathrm{Z}_1$	*Rate: MAK(k=0.6781)*

4 Task: Classifiers

This section describes CRNs optimized to perform classification operations across initial conditions. The output concentration of species X_1 and X_2 represent the classes.

4.1 Configuration: 2 Species, 6 Reactions

I/O CRN 1

Loss: 0.00059
Inputs: $\{u_1\}$ **Species:** $\{X_1, X_2\}$ **Output:** $\{X_1, X_2\}$

$\varnothing \xrightarrow{2.15} \varnothing$	*Rate: MAK(k=2.1519)*
$\mathrm{X}_1 \xrightarrow{u_1} \varnothing$	*Rate: MAK(k=1.0, u_1)*
$\mathrm{X}_1 \xrightarrow{5.10} \mathrm{X}_1 + \mathrm{X}_1$	*Rate: MAK(k=5.1015)*
$\mathrm{X}_2 \xrightarrow{1.00} \varnothing$	*Rate: MAK(k=1.0)*
$\mathrm{X}_2 \xrightarrow{5.61} \mathrm{X}_2 + \mathrm{X}_2$	*Rate: MAK(k=5.6112)*
$\mathrm{X}_1 + \mathrm{X}_1 \xrightarrow{2.05} \varnothing$	*Rate: MAK(k=2.0498)*
$\mathrm{X}_1 + \mathrm{X}_2 \xrightarrow{15.79} \varnothing$	*Rate: MAK(k=15.7926)*
$\mathrm{X}_2 + \mathrm{X}_2 \xrightarrow{2.30} \varnothing$	*Rate: MAK(k=2.3049)*

I/O CRN 11

Loss: 0.00193
Inputs: $\{u_1\}$ **Species:** $\{X_1, X_2\}$ **Output:** $\{X_1, X_2\}$

$\varnothing \xrightarrow{1.57} \varnothing$	*Rate: MAK(k=1.5695)*
$X_1 \xrightarrow{u_1} \varnothing$	*Rate: MAK(k=1.0, u_1)*
$X_1 \xrightarrow{2.56} X_1 + X_1$	*Rate: MAK(k=2.5650)*
$X_2 \xrightarrow{1.00} \varnothing$	*Rate: MAK(k=1.0)*
$X_2 \xrightarrow{2.67} X_2 + X_2$	*Rate: MAK(k=2.6720)*
$X_1 + X_1 \xrightarrow{1.56} X_1$	*Rate: MAK(k=1.5628)*
$X_1 + X_2 \xrightarrow{4.01} \varnothing$	*Rate: MAK(k=4.0056)*
$X_2 + X_2 \xrightarrow{1.67} X_2$	*Rate: MAK(k=1.6697)*

I/O CRN 14

Loss: 0.00197
Inputs: $\{u_1\}$ **Species:** $\{X_1, X_2\}$ **Output:** $\{X_1, X_2\}$

$X_1 \xrightarrow{u_1} \varnothing$	*Rate: MAK(k=1.0, u_1)*
$X_1 \xrightarrow{3.73} X_1 + X_1$	*Rate: MAK(k=3.7344)*
$X_2 \xrightarrow{1.00} \varnothing$	*Rate: MAK(k=1.0)*
$X_2 \xrightarrow{4.34} X_2 + X_2$	*Rate: MAK(k=4.3372)*
$X_1 + X_1 \xrightarrow{1.37} \varnothing$	*Rate: MAK(k=1.3690)*
$X_1 + X_2 \xrightarrow{13.15} \varnothing$	*Rate: MAK(k=13.1543)*
$X_1 + X_2 \xrightarrow{1.54} X_1$	*Rate: MAK(k=1.5375)*
$X_2 + X_2 \xrightarrow{1.66} \varnothing$	*Rate: MAK(k=1.6614)*

5 Task: Logic Circuits

This section describes CRNs optimized to perform logic operations across four chemical inputs. The output concentration of species OUT represents the boolean result of the computation.

5.1 Configuration: 5 Species, 5 Reactions

I/O CRN 1

Loss: 0.02127
Inputs: $\{u_1, u_2, u_3, u_4\}$ **Species:** $\{\mathrm{OUT}, X_1, X_2, X_3, X_4\}$ **Output:** OUT

$$\varnothing \xrightarrow{u_1} \mathrm{X}_1 \qquad \textit{Rate: MAK(k=1.0, } u_1\textit{)}$$
$$\varnothing \xrightarrow{u_2} \mathrm{X}_2 \qquad \textit{Rate: MAK(k=1.0, } u_2\textit{)}$$
$$\varnothing \xrightarrow{u_3} \mathrm{X}_3 \qquad \textit{Rate: MAK(k=1.0, } u_3\textit{)}$$
$$\varnothing \xrightarrow{u_4} \mathrm{X}_4 \qquad \textit{Rate: MAK(k=1.0, } u_4\textit{)}$$
$$\mathrm{X}_3 \xrightarrow{1.01} \mathrm{X}_1 \qquad \textit{Rate: MAK(k=1.0138)}$$
$$\mathrm{OUT} \xrightarrow{0.94} \mathrm{OUT} + \mathrm{OUT} \qquad \textit{Rate: MAK(k=0.9369)}$$
$$\mathrm{X}_1 + \mathrm{X}_2 \xrightarrow{0.99} \mathrm{OUT} \qquad \textit{Rate: MAK(k=0.9904)}$$
$$\mathrm{X}_3 + \mathrm{X}_4 \xrightarrow{1.24} \mathrm{OUT} \qquad \textit{Rate: MAK(k=1.2422)}$$
$$\mathrm{OUT} + \mathrm{OUT} \xrightarrow{1.95} \mathrm{OUT} \qquad \textit{Rate: MAK(k=1.9500)}$$

I/O CRN 6

Loss: 0.02274
Inputs: $\{u_1, u_2, u_3, u_4\}$ **Species:** $\{\mathrm{OUT}, X_1, X_2, X_3, X_4\}$

$$\varnothing \xrightarrow{u_1} \mathrm{X}_1 \qquad \textit{Rate: MAK(k=1.0, } u_1\textit{)}$$
$$\varnothing \xrightarrow{u_2} \mathrm{X}_2 \qquad \textit{Rate: MAK(k=1.0, } u_2\textit{)}$$
$$\varnothing \xrightarrow{u_3} \mathrm{X}_3 \qquad \textit{Rate: MAK(k=1.0, } u_3\textit{)}$$
$$\varnothing \xrightarrow{u_4} \mathrm{X}_4 \qquad \textit{Rate: MAK(k=1.0, } u_4\textit{)}$$
$$\mathrm{X}_1 + \mathrm{X}_2 \xrightarrow{0.27} \mathrm{OUT} \qquad \textit{Rate: MAK(k=0.2697)}$$
$$\mathrm{X}_2 + \mathrm{X}_3 \xrightarrow{1.02} \mathrm{X}_1 + \mathrm{X}_4 \qquad \textit{Rate: MAK(k=1.0214)}$$
$$\mathrm{X}_3 + \mathrm{X}_4 \xrightarrow{0.95} \mathrm{OUT} \qquad \textit{Rate: MAK(k=0.9495)}$$
$$\mathrm{X}_4 + \mathrm{X}_4 \xrightarrow{0.92} \mathrm{X}_4 \qquad \textit{Rate: MAK(k=0.9154)}$$
$$\mathrm{OUT} + \mathrm{OUT} \xrightarrow{1.00} \mathrm{OUT} \qquad \textit{Rate: MAK(k=0.9960)}$$

I/O CRN 26

Loss: 0.02468
Inputs: $\{u_1, u_2, u_3, u_4\}$ **Species:** $\{\mathrm{OUT}, X_1, X_2, X_3, X_4\}$

$$\varnothing \xrightarrow{u_1} \mathrm{X}_1 \quad \textit{Rate: MAK(k=1.0, } u_1\textit{)}$$
$$\varnothing \xrightarrow{u_2} \mathrm{X}_2 \quad \textit{Rate: MAK(k=1.0, } u_2\textit{)}$$
$$\varnothing \xrightarrow{u_3} \mathrm{X}_3 \quad \textit{Rate: MAK(k=1.0, } u_3\textit{)}$$
$$\varnothing \xrightarrow{u_4} \mathrm{X}_4 \quad \textit{Rate: MAK(k=1.0, } u_4\textit{)}$$
$$\mathrm{X}_1 + \mathrm{X}_2 \xrightarrow{0.52} \mathrm{OUT} \quad \textit{Rate: MAK(k=0.5205)}$$
$$\mathrm{X}_2 + \mathrm{X}_3 \xrightarrow{1.29} \mathrm{X}_3 + \mathrm{X}_4 \quad \textit{Rate: MAK(k=1.2892)}$$
$$\mathrm{X}_3 + \mathrm{X}_4 \xrightarrow{0.75} \mathrm{OUT} \quad \textit{Rate: MAK(k=0.7477)}$$
$$\mathrm{X}_4 + \mathrm{X}_4 \xrightarrow{0.75} \mathrm{X}_4 \quad \textit{Rate: MAK(k=0.7470)}$$
$$\mathrm{OUT} + \mathrm{OUT} \xrightarrow{0.99} \mathrm{OUT} \quad \textit{Rate: MAK(k=0.9903)}$$

5.2 Configuration: 5 Species, 6 Reactions

I/O CRN 1

Loss: 0.00581
Inputs: $\{u_1, u_2, u_3, u_4\}$ **Species:** $\{\mathrm{OUT}, X_1, X_2, X_3, X_4\}$

$$\varnothing \xrightarrow{u_1} \mathrm{X}_1 \quad \textit{Rate: MAK(k=1.0, } u_1\textit{)}$$
$$\varnothing \xrightarrow{u_2} \mathrm{X}_2 \quad \textit{Rate: MAK(k=1.0, } u_2\textit{)}$$
$$\varnothing \xrightarrow{u_3} \mathrm{X}_3 \quad \textit{Rate: MAK(k=1.0, } u_3\textit{)}$$
$$\varnothing \xrightarrow{u_4} \mathrm{X}_4 \quad \textit{Rate: MAK(k=1.0, } u_4\textit{)}$$
$$\mathrm{X}_1 + \mathrm{X}_1 \xrightarrow{5.18} \mathrm{X}_1 \quad \textit{Rate: MAK(k=5.1791)}$$
$$\mathrm{X}_1 + \mathrm{X}_2 \xrightarrow{0.49} \mathrm{OUT} \quad \textit{Rate: MAK(k=0.4917)}$$
$$\mathrm{X}_2 + \mathrm{X}_3 \xrightarrow{0.79} \mathrm{X}_1 + \mathrm{X}_2 \quad \textit{Rate: MAK(k=0.7890)}$$
$$\mathrm{X}_2 + \mathrm{X}_4 \xrightarrow{5.70} \mathrm{X}_2 \quad \textit{Rate: MAK(k=5.6951)}$$
$$\mathrm{X}_3 + \mathrm{X}_4 \xrightarrow{0.54} \mathrm{OUT} \quad \textit{Rate: MAK(k=0.5447)}$$
$$\mathrm{OUT} + \mathrm{OUT} \xrightarrow{0.50} \mathrm{X}_1 \quad \textit{Rate: MAK(k=0.4978)}$$

I/O CRN 3

Loss: 0.00793
Inputs: $\{u_1, u_2, u_3, u_4\}$ **Species:** $\{\mathrm{OUT}, X_1, X_2, X_3, X_4\}$

$$\varnothing \xrightarrow{u_1} \mathrm{X}_1$$ *Rate: MAK(k=1.0, u_1)*

$$\varnothing \xrightarrow{u_2} \mathrm{X}_2$$ *Rate: MAK(k=1.0, u_2)*

$$\varnothing \xrightarrow{u_3} \mathrm{X}_3$$ *Rate: MAK(k=1.0, u_3)*

$$\varnothing \xrightarrow{u_4} \mathrm{X}_4$$ *Rate: MAK(k=1.0, u_4)*

$$\mathrm{X}_1 + \mathrm{X}_2 \xrightarrow{0.10} \mathrm{OUT}$$ *Rate: MAK(k=0.1032)*

$$\mathrm{X}_1 + \mathrm{X}_3 \xrightarrow{0.74} \mathrm{X}_3$$ *Rate: MAK(k=0.7439)*

$$\mathrm{X}_2 + \mathrm{X}_3 \xrightarrow{3.66} \mathrm{X}_3 + \mathrm{X}_4$$ *Rate: MAK(k=3.6633)*

$$\mathrm{X}_3 + \mathrm{X}_4 \xrightarrow{1.04} \mathrm{OUT}$$ *Rate: MAK(k=1.0372)*

$$\mathrm{X}_4 + \mathrm{X}_4 \xrightarrow{0.74} \varnothing$$ *Rate: MAK(k=0.7436)*

$$\mathrm{OUT} + \mathrm{OUT} \xrightarrow{0.98} \mathrm{OUT}$$ *Rate: MAK(k=0.9819)*

I/O CRN 8

Loss: 0.01125
Inputs: $\{u_1, u_2, u_3, u_4\}$ **Species:** $\{\mathrm{OUT}, X_1, X_2, X_3, X_4\}$

$$\varnothing \xrightarrow{u_1} \mathrm{X}_1$$ *Rate: MAK(k=1.0, u_1)*

$$\varnothing \xrightarrow{u_2} \mathrm{X}_2$$ *Rate: MAK(k=1.0, u_2)*

$$\varnothing \xrightarrow{u_3} \mathrm{X}_3$$ *Rate: MAK(k=1.0, u_3)*

$$\varnothing \xrightarrow{u_4} \mathrm{X}_4$$ *Rate: MAK(k=1.0, u_4)*

$$\mathrm{X}_1 + \mathrm{X}_2 \xrightarrow{0.10} \mathrm{OUT}$$ *Rate: MAK(k=0.1023)*

$$\mathrm{X}_1 + \mathrm{X}_3 \xrightarrow{11.48} \mathrm{X}_3$$ *Rate: MAK(k=11.4839)*

$$\mathrm{X}_2 + \mathrm{X}_3 \xrightarrow{5.53} \mathrm{OUT}$$ *Rate: MAK(k=5.5349)*

$$\mathrm{X}_3 + \mathrm{X}_4 \xrightarrow{0.21} \mathrm{OUT}$$ *Rate: MAK(k=0.2072)*

$$\mathrm{X}_4 + \mathrm{X}_4 \xrightarrow{0.77} \mathrm{X}_4$$ *Rate: MAK(k=0.7732)*

$$\mathrm{OUT} + \mathrm{OUT} \xrightarrow{0.51} \varnothing$$ *Rate: MAK(k=0.5060)*

5.3 Configuration: 6 Species, 6 Reactions, non-zero initial condition

I/O CRN 1

Inputs: $\{u_1, u_2, u_3, u_4\}$ **Species:** $\{X_1, X_2, X_3, X_4, X_5\}$ **Output:** $\mathrm{OUT} = \mathrm{X}_5$

$\varnothing \xrightarrow{u_1} \mathrm{X}_1$	*Rate: MAK(k=1.0, u_1)*
$\varnothing \xrightarrow{u_2} \mathrm{X}_2$	*Rate: MAK(k=1.0, u_2)*
$\varnothing \xrightarrow{u_3} \mathrm{X}_3$	*Rate: MAK(k=1.0, u_3)*
$\varnothing \xrightarrow{u_4} \mathrm{X}_4$	*Rate: MAK(k=1.0, u_4)*
$\mathrm{X}_1 + \mathrm{X}_1 \xrightarrow{5.18} \mathrm{X}_1$	*Rate: MAK(k=5.1791)*
$\mathrm{X}_1 + \mathrm{X}_2 \xrightarrow{0.49} \mathrm{OUT}$	*Rate: MAK(k=0.4917)*
$\mathrm{X}_2 + \mathrm{X}_3 \xrightarrow{0.79} \mathrm{X}_1 + \mathrm{X}_2$	*Rate: MAK(k=0.7890)*
$\mathrm{X}_2 + \mathrm{X}_4 \xrightarrow{5.70} \mathrm{X}_2$	*Rate: MAK(k=5.6951)*
$\mathrm{X}_3 + \mathrm{X}_4 \xrightarrow{0.54} \mathrm{OUT}$	*Rate: MAK(k=0.5447)*
$\mathrm{OUT} + \mathrm{OUT} \xrightarrow{0.50} \mathrm{X}_1$	*Rate: MAK(k=0.4978)*

I/O CRN 3

Inputs: $\{u_1, u_2, u_3, u_4\}$ **Species:** $\{\mathrm{OUT}, X_1, X_2, X_3, X_4\}$ **Output:** $\mathrm{OUT} = \mathrm{X}_5$

$\varnothing \xrightarrow{u_1} \mathrm{X}_1$	*Rate: MAK(k=1.0, u_1)*
$\varnothing \xrightarrow{u_2} \mathrm{X}_2$	*Rate: MAK(k=1.0, u_2)*
$\varnothing \xrightarrow{u_3} \mathrm{X}_3$	*Rate: MAK(k=1.0, u_3)*
$\varnothing \xrightarrow{u_4} \mathrm{X}_4$	*Rate: MAK(k=1.0, u_4)*
$\mathrm{X}_1 + \mathrm{X}_2 \xrightarrow{0.10} \mathrm{OUT}$	*Rate: MAK(k=0.1032)*
$\mathrm{X}_1 + \mathrm{X}_3 \xrightarrow{0.74} \mathrm{X}_3$	*Rate: MAK(k=0.7439)*
$\mathrm{X}_2 + \mathrm{X}_3 \xrightarrow{3.66} \mathrm{X}_3 + \mathrm{X}_4$	*Rate: MAK(k=3.6633)*
$\mathrm{X}_3 + \mathrm{X}_4 \xrightarrow{1.04} \mathrm{OUT}$	*Rate: MAK(k=1.0372)*
$\mathrm{X}_4 + \mathrm{X}_4 \xrightarrow{0.74} \varnothing$	*Rate: MAK(k=0.7436)*
$\mathrm{OUT} + \mathrm{OUT} \xrightarrow{0.98} \mathrm{OUT}$	*Rate: MAK(k=0.9819)*

I/O CRN 8

Inputs: $\{u_1, u_2, u_3, u_4\}$ **Species:** $\{X_1, X_2, X_3, X_4, X_5\}$ **Output:** $\mathrm{OUT} = \mathrm{X}_5$

$$\varnothing \xrightarrow{u_1} \mathrm{X}_1 \qquad \textit{Rate: MAK(k=1.0, } u_1\textit{)}$$
$$\varnothing \xrightarrow{u_2} \mathrm{X}_2 \qquad \textit{Rate: MAK(k=1.0, } u_2\textit{)}$$
$$\varnothing \xrightarrow{u_3} \mathrm{X}_3 \qquad \textit{Rate: MAK(k=1.0, } u_3\textit{)}$$
$$\varnothing \xrightarrow{u_4} \mathrm{X}_4 \qquad \textit{Rate: MAK(k=1.0, } u_4\textit{)}$$
$$\mathrm{X}_1 + \mathrm{X}_2 \xrightarrow{0.10} \mathrm{OUT} \qquad \textit{Rate: MAK(k=0.1023)}$$
$$\mathrm{X}_1 + \mathrm{X}_3 \xrightarrow{11.48} \mathrm{X}_3 \qquad \textit{Rate: MAK(k=11.4839)}$$
$$\mathrm{X}_2 + \mathrm{X}_3 \xrightarrow{5.53} \mathrm{OUT} \qquad \textit{Rate: MAK(k=5.5349)}$$
$$\mathrm{X}_3 + \mathrm{X}_4 \xrightarrow{0.21} \mathrm{OUT} \qquad \textit{Rate: MAK(k=0.2072)}$$
$$\mathrm{X}_4 + \mathrm{X}_4 \xrightarrow{0.77} \mathrm{X}_4 \qquad \textit{Rate: MAK(k=0.7732)}$$
$$\mathrm{OUT} + \mathrm{OUT} \xrightarrow{0.51} \varnothing \qquad \textit{Rate: MAK(k=0.5060)}$$

5.4 Configuration: 6 Species, 6 Reactions (Hill Functions)

I/O CRN 1

Loss: 0.00114
Inputs: $\{u_1, u_2, u_3, u_4\}$ **Species:** $\{X_1, X_2, X_3, X_4, X_5, X_6\}$ **Output:** $\mathrm{OUT} = \mathrm{X}_6$

$$\varnothing \longrightarrow \mathrm{X}_1 \quad \textit{Rate: HILLProd}(b = 0,\ Vm = 1.4108,\ (Ka, na) = X_3(0.0057, 1),\ (Kr, nr) = X_5(0.0049, 1))$$
$$\varnothing \longrightarrow \mathrm{X}_1 \quad \textit{Rate: HILLProd}(b = 0,\ Vm = 1.9852,\ (Ka, na) = X_3(0.8058, 1),\ X_5(0.2614, 1),\ (Kr, nr) =)$$
$$\varnothing \longrightarrow \mathrm{X}_2 \quad \textit{Rate: HILLProd}(b = 0,\ Vm = 1.3947,\ (Ka, na) = X_3(1.0786, 1),\ X_4(0.2897, 1),\ (Kr, nr) =)$$
$$\varnothing \longrightarrow \mathrm{X}_5 \quad \textit{Rate: HILLProd}(b = 0,\ Vm = 3.7648,\ (Ka, na) = X_1(0.0179, 1),\ (Kr, nr) = X_4(0.0002, 1))$$
$$\varnothing \longrightarrow \mathrm{X}_5 \quad \textit{Rate: HILLProd}(b = 0,\ Vm = 0.2577,\ (Ka, na) = X_4(0.1335, 1),\ (Kr, nr) = OUT(0.0002, 1))$$
$$\varnothing \longrightarrow \mathrm{OUT} \quad \textit{Rate: HILLProd}(b = 0,\ Vm = 1.0073,\ (Ka, na) = X_1(0.0076, 1),\ X_2(0.0026, 1),\ (Kr, nr) =)$$
$$\varnothing \xrightarrow{u_1} \mathrm{X}_1 \quad \textit{Rate: MAK(k=1.0, } u_1\textit{)}$$
$$\varnothing \xrightarrow{u_2} \mathrm{X}_2 \quad \textit{Rate: MAK(k=1.0, } u_2\textit{)}$$
$$\varnothing \xrightarrow{u_3} \mathrm{X}_3 \quad \textit{Rate: MAK(k=1.0, } u_3\textit{)}$$
$$\varnothing \xrightarrow{u_4} \mathrm{X}_4 \quad \textit{Rate: MAK(k=1.0, } u_4\textit{)}$$
$$\mathrm{X}_1 \xrightarrow{1.00} \varnothing \quad \textit{Rate: MAK(k=1.0)}$$
$$\mathrm{X}_2 \xrightarrow{1.00} \varnothing \quad \textit{Rate: MAK(k=1.0)}$$
$$\mathrm{X}_3 \xrightarrow{1.00} \varnothing \quad \textit{Rate: MAK(k=1.0)}$$
$$\mathrm{X}_4 \xrightarrow{1.00} \varnothing \quad \textit{Rate: MAK(k=1.0)}$$
$$\mathrm{X}_5 \xrightarrow{1.00} \varnothing \quad \textit{Rate: MAK(k=1.0)}$$
$$\mathrm{OUT} \xrightarrow{1.00} \varnothing \quad \textit{Rate: MAK(k=1.0)}$$

I/O CRN 15

Loss: 0.00195
Inputs: $\{u_1, u_2, u_3, u_4\}$ **Species:** $\{X_1, X_2, X_3, X_4, X_5, X_6\}$ **Output:** $\mathrm{OUT} = \mathrm{X}_6$

$\varnothing \longrightarrow \mathrm{X}_1$ *Rate:* $HILLProd(b=0,\ Vm=2.2641,\ (Ka,na)=X_3(0.0129,1),\ X_5(0.0077,1),\ (Kr,nr)=)$

$\varnothing \longrightarrow \mathrm{X}_2$ *Rate:* $HILLProd(b=0,\ Vm=1.3628,\ (Ka,na)=X_3(0.0211,1),\ X_4(0.5656,1),\ (Kr,nr)=)$

$\varnothing \longrightarrow \mathrm{X}_5$ *Rate:* $HILLProd(b=0,\ Vm=0.9270,\ (Ka,na)=,\ (Kr,nr)=X_4(0.0017,1),\ X_5(0.0218,1))$

$\varnothing \longrightarrow \mathrm{X}_5$ *Rate:* $HILLProd(b=0,\ Vm=0.8529,\ (Ka,na)=X_5(0.1611,1),\ (Kr,nr)=OUT(0.0642,1))$

$\varnothing \longrightarrow \mathrm{X}_5$ *Rate:* $HILLProd(b=0,\ Vm=1.6412,\ (Ka,na)=OUT(0.2783,1),\ X_2(0.0216,1),\ (Kr,nr)=)$

$\varnothing \longrightarrow \mathrm{OUT}$ *Rate:* $HILLProd(b=0,\ Vm=1.0160,\ (Ka,na)=X_1(0.0110,1),\ X_2(0.0104,1),\ (Kr,nr)=)$

$\varnothing \xrightarrow{u_1} \mathrm{X}_1$ *Rate: MAK(k=1.0, u_1)*

$\varnothing \xrightarrow{u_2} \mathrm{X}_2$ *Rate: MAK(k=1.0, u_2)*

$\varnothing \xrightarrow{u_3} \mathrm{X}_3$ *Rate: MAK(k=1.0, u_3)*

$\varnothing \xrightarrow{u_4} \mathrm{X}_4$ *Rate: MAK(k=1.0, u_4)*

$\mathrm{X}_1 \xrightarrow{1.00} \varnothing$ *Rate: MAK(k=1.0)*

$\mathrm{X}_2 \xrightarrow{1.00} \varnothing$ *Rate: MAK(k=1.0)*

$\mathrm{X}_3 \xrightarrow{1.00} \varnothing$ *Rate: MAK(k=1.0)*

$\mathrm{X}_4 \xrightarrow{1.00} \varnothing$ *Rate: MAK(k=1.0)*

$\mathrm{X}_5 \xrightarrow{1.00} \varnothing$ *Rate: MAK(k=1.0)*

$\mathrm{OUT} \xrightarrow{1.00} \varnothing$ *Rate: MAK(k=1.0)*

I/O CRN 30

Loss: 0.00256
Inputs: $\{u_1, u_2, u_3, u_4\}$ **Species:** $\{X_1, X_2, X_3, X_4, X_5, X_6\}$ **Output:** $\mathrm{OUT} = \mathrm{X}_6$

$\varnothing \longrightarrow \mathrm{X}_1$ *Rate:* $HILLProd(b=0,\ Vm=1.1414,\ (Ka,na)=X_3(0.0044,1),\ X_5(0.1256,1),\ (Kr,nr)=)$

$\varnothing \longrightarrow \mathrm{X}_2$ *Rate:* $HILLProd(b=0,\ Vm=4.2466,\ (Ka,na)=X_3(0.0607,1),\ X_4(0.0016,1),\ (Kr,nr)=)$

$\varnothing \longrightarrow \mathrm{X}_5$ *Rate:* $HILLProd(b=0,\ Vm=0.3768,\ (Ka,na)=X_4(0.0061,1),\ (Kr,nr)=X_5(0.0001,1))$

$\varnothing \longrightarrow \mathrm{X}_5$ *Rate:* $HILLProd(b=0,\ Vm=0.8472,\ (Ka,na)=OUT(0.0333,1),\ (Kr,nr)=X_5(0.1009,1))$

$\varnothing \longrightarrow \mathrm{X}_5$ *Rate:* $HILLProd(b=0,\ Vm=0.7262,\ (Ka,na)=X_4(0.0015,1),\ X_5(0.0005,1),\ (Kr,nr)=)$

$\varnothing \longrightarrow \mathrm{OUT}$ *Rate:* $HILLProd(b=0,\ Vm=1.0044,\ (Ka,na)=X_1(0.0007,1),\ X_2(0.0089,1),\ (Kr,nr)=)$

$\varnothing \xrightarrow{u_1} \mathrm{X}_1$ *Rate: MAK(k=1.0, u_1)*

$\varnothing \xrightarrow{u_2} \mathrm{X}_2$ *Rate: MAK(k=1.0, u_2)*

$\varnothing \xrightarrow{u_3} \mathrm{X}_3$ *Rate: MAK(k=1.0, u_3)*

$\varnothing \xrightarrow{u_4} \mathrm{X}_4$ *Rate: MAK(k=1.0, u_4)*

$\mathrm{X}_1 \xrightarrow{1.00} \varnothing$ *Rate: MAK(k=1.0)*

$\mathrm{X}_2 \xrightarrow{1.00} \varnothing$ *Rate: MAK(k=1.0)*

$\mathrm{X}_3 \xrightarrow{1.00} \varnothing$ *Rate: MAK(k=1.0)*

$\mathrm{X}_4 \xrightarrow{1.00} \varnothing$ *Rate: MAK(k=1.0)*

$\mathrm{X}_5 \xrightarrow{1.00} \varnothing$ *Rate: MAK(k=1.0)*

$\mathrm{OUT} \xrightarrow{1.00} \varnothing$ *Rate: MAK(k=1.0)*

6 Task: Oscillators

This section details the In-Silico Optimized Chemical Reaction Networks (I/O CRNs) discovered for the Oscillator task, where the system is designed to produce sustained periodic fluctuations in the output species.

6.1 Configuration: 3 Species, 6 Reactions

I/O CRN 8

Loss: 0.00587
Inputs: $\{u_1\}$ **Species:** $\{X_1, X_2, X_3\}$ **Output:** X_3

$$\varnothing \xrightarrow{u_1} X_1 \qquad \textit{Rate: MAK(k=1.0, } u_1\textit{)}$$
$$X_1 \xrightarrow{1.00} \varnothing \qquad \textit{Rate: MAK(k=1)}$$
$$X_2 \xrightarrow{1.00} \varnothing \qquad \textit{Rate: MAK(k=1)}$$
$$X_2 \xrightarrow{15.49} X_2 + X_2 \qquad \textit{Rate: MAK(k=15.4936)}$$
$$X_3 \xrightarrow{1.00} \varnothing \qquad \textit{Rate: MAK(k=1)}$$
$$X_1 + X_1 \xrightarrow{15.78} X_2 \qquad \textit{Rate: MAK(k=15.7776)}$$
$$X_1 + X_1 \xrightarrow{15.69} X_2 + X_3 \qquad \textit{Rate: MAK(k=15.6906)}$$
$$X_1 + X_3 \xrightarrow{15.16} \varnothing \qquad \textit{Rate: MAK(k=15.1622)}$$
$$X_1 + X_3 \xrightarrow{15.53} X_1 \qquad \textit{Rate: MAK(k=15.5301)}$$
$$X_2 + X_3 \xrightarrow{15.84} X_3 + X_3 \qquad \textit{Rate: MAK(k=15.8353)}$$

I/O CRN 10

Loss: 0.00617
Inputs: $\{u_1\}$ **Species:** $\{X_1, X_2, X_3\}$ **Output:** X_3

$$\varnothing \xrightarrow{u_1} X_1 \qquad \textit{Rate: MAK(k=1.0, } u_1\textit{)}$$
$$X_1 \xrightarrow{1.00} \varnothing \qquad \textit{Rate: MAK(k=1)}$$
$$X_1 \xrightarrow{17.88} X_1 + X_1 \qquad \textit{Rate: MAK(k=17.8764)}$$
$$X_2 \xrightarrow{1.00} \varnothing \qquad \textit{Rate: MAK(k=1)}$$
$$X_2 \xrightarrow{18.03} X_2 + X_2 \qquad \textit{Rate: MAK(k=18.0313)}$$
$$X_3 \xrightarrow{1.00} \varnothing \qquad \textit{Rate: MAK(k=1)}$$
$$X_1 + X_1 \xrightarrow{18.22} X_1 + X_2 \qquad \textit{Rate: MAK(k=18.2249)}$$
$$X_1 + X_2 \xrightarrow{17.99} X_1 \qquad \textit{Rate: MAK(k=17.9876)}$$
$$X_1 + X_3 \xrightarrow{17.91} \varnothing \qquad \textit{Rate: MAK(k=17.9131)}$$
$$X_2 + X_3 \xrightarrow{17.70} X_3 + X_3 \qquad \textit{Rate: MAK(k=17.7040)}$$

I/O CRN 100

Loss: 0.01307
Inputs: $\{u_1\}$ **Species:** $\{X_1, X_2, X_3\}$ **Output:** X_3

$\varnothing \xrightarrow{u_1} \mathrm{X}_1$	*Rate: MAK(k=1.0, u_1)*
$\mathrm{X}_1 \xrightarrow{1.00} \varnothing$	*Rate: MAK(k=1)*
$\mathrm{X}_2 \xrightarrow{1.00} \varnothing$	*Rate: MAK(k=1)*
$\mathrm{X}_2 \xrightarrow{15.89} \mathrm{X}_2 + \mathrm{X}_2$	*Rate: MAK(k=15.8920)*
$\mathrm{X}_3 \xrightarrow{1.00} \varnothing$	*Rate: MAK(k=1)*
$\mathrm{X}_1 + \mathrm{X}_1 \xrightarrow{16.06} \mathrm{X}_1 + \mathrm{X}_2$	*Rate: MAK(k=16.0649)*
$\mathrm{X}_1 + \mathrm{X}_1 \xrightarrow{15.91} \mathrm{X}_1 + \mathrm{X}_3$	*Rate: MAK(k=15.9058)*
$\mathrm{X}_1 + \mathrm{X}_2 \xrightarrow{15.80} \mathrm{X}_2 + \mathrm{X}_2$	*Rate: MAK(k=15.7976)*
$\mathrm{X}_1 + \mathrm{X}_3 \xrightarrow{15.64} \varnothing$	*Rate: MAK(k=15.6434)*
$\mathrm{X}_2 + \mathrm{X}_3 \xrightarrow{16.25} \mathrm{X}_3 + \mathrm{X}_3$	*Rate: MAK(k=16.2546)*

6.2 Configuration: 3 Species, 6 Reactions (Frequency Tuning)

I/O CRN 3

Loss: 0.06399
Inputs: $\{u_1\}$ **Species:** $\{X_1, X_2, X_3\}$ **Output:** X_3

$\varnothing \xrightarrow{1.66} \varnothing$	*Rate: MAK(k=1.66)*
$\varnothing \xrightarrow{u_1} \mathrm{X}_1$	*Rate: MAK(k=1.0, u_1)*
$\mathrm{X}_1 \xrightarrow{1.00} \varnothing$	*Rate: MAK(k=1.0)*
$\mathrm{X}_2 \xrightarrow{1.00} \varnothing$	*Rate: MAK(k=1.0)*
$\mathrm{X}_2 \xrightarrow{1.71} \mathrm{X}_1$	*Rate: MAK(k=1.7973)*
$\mathrm{X}_3 \xrightarrow{1.00} \varnothing$	*Rate: MAK(k=1.0)*
$\mathrm{X}_3 \xrightarrow{1.86} \mathrm{X}_3 + \mathrm{X}_3$	*Rate: MAK(k=1.8626)*
$\mathrm{X}_1 + \mathrm{X}_1 \xrightarrow{2.49} \mathrm{X}_2 + \mathrm{X}_2$	*Rate: MAK(k=2.4929)*
$\mathrm{X}_2 + \mathrm{X}_3 \xrightarrow{7.5} \mathrm{X}_2$	*Rate: MAK(k=7.4959)*
$\mathrm{X}_2 + \mathrm{X}_3 \xrightarrow{2.06} \mathrm{X}_1 + \mathrm{X}_2$	*Rate: MAK(k=2.0564)*

I/O CRN 39

Loss: 0.07286
Inputs: $\{u_1\}$ **Species:** $\{X_1, X_2, X_3\}$ **Output:** X_3

$$\varnothing \xrightarrow{u_1} X_1 \qquad \textit{Rate: MAK(k=1.0, } u_1\textit{)}$$

$$X_1 \xrightarrow{1.00} \varnothing \qquad \textit{Rate: MAK(k=1.0)}$$

$$X_2 \xrightarrow{1.00} \varnothing \qquad \textit{Rate: MAK(k=1.0)}$$

$$X_3 \xrightarrow{1.00} \varnothing \qquad \textit{Rate: MAK(k=1.0)}$$

$$X_3 \xrightarrow{2.13} X_1 + X_3 \qquad \textit{Rate: MAK(k=2.1282)}$$

$$X_1 + X_1 \xrightarrow{4.81} X_1 + X_3 \qquad \textit{Rate: MAK(k=4.8111)}$$

$$X_1 + X_1 \xrightarrow{2.62} X_3 + X_3 \qquad \textit{Rate: MAK(k=2.6216)}$$

$$X_1 + X_3 \xrightarrow{2.58} X_1 + X_1 \qquad \textit{Rate: MAK(k=2.5795)}$$

$$X_2 + X_3 \xrightarrow{4.8} X_2 + X_2 \qquad \textit{Rate: MAK(k=4.7978)}$$

I/O CRN 51

Loss: 0.07583
Inputs: $\{u_1\}$ **Species:** $\{X_1, X_2, X_3\}$ **Output:** X_3

$$\varnothing \xrightarrow{u_1} X_1 \qquad \textit{Rate: MAK(k=1.0, } u_1\textit{)}$$

$$X_1 \xrightarrow{1.00} \varnothing \qquad \textit{Rate: MAK(k=1.0)}$$

$$X_2 \xrightarrow{1.00} \varnothing \qquad \textit{Rate: MAK(k=1.0)}$$

$$X_3 \xrightarrow{1.00} \varnothing \qquad \textit{Rate: MAK(k=1.0)}$$

$$X_3 \xrightarrow{2.81} X_1 + X_3 \qquad \textit{Rate: MAK(k=2.8096)}$$

$$X_1 + X_1 \xrightarrow{3.10} X_3 + X_3 \qquad \textit{Rate: MAK(k=3.0999)}$$

$$X_1 + X_2 \xrightarrow{3.66} X_2 \qquad \textit{Rate: MAK(k=3.6616)}$$

$$X_1 + X_3 \xrightarrow{2.21} X_1 + X_1 \qquad \textit{Rate: MAK(k=2.2070)}$$

$$X_2 + X_3 \xrightarrow{6.77} X_2 + X_2 \qquad \textit{Rate: MAK(k=6.7750)}$$

$$X_3 + X_3 \xrightarrow{1.68} X_1 + X_3 \qquad \textit{Rate: MAK(k=1.6773)}$$

7 Task: Habituation and Sensitization

This section reports the In-Silico Optimized Chemical Reaction Networks (I/O CRNs) discovered for habituation and sensitization. In both tasks, the system is stimulated by repeated pulses of the input u_1 (with an inter-train OFF gap), and the output species X_3 is optimized to exhibit either decreasing (habituation) or increasing (sensitization) peak responses across successive pulses.

7.1 Habituation

I/O CRN 1

Inputs: $\{u_1\}$ **Species:** $\{X_1, X_2, X_3\}$ **Output:** X_3

$\varnothing \xrightarrow{1.23} \varnothing$	*Rate: MAK(k=1.2254)*
$\varnothing \xrightarrow{u_1} \mathrm{X}_1$	*Rate: MAK(k=1.0,* u_1*)*
$\varnothing \xrightarrow{0.10} \mathrm{X}_2$	*Rate: MAK(k=0.1)*
$\mathrm{X}_1 \xrightarrow{0.10} \varnothing$	*Rate: MAK(k=0.1)*
$\mathrm{X}_1 \xrightarrow{51.83} \mathrm{X}_1 + \mathrm{X}_3$	*Rate: MAK(k=51.8283)*
$\mathrm{X}_2 \xrightarrow{0.10} \varnothing$	*Rate: MAK(k=0.1)*
$\mathrm{X}_3 \xrightarrow{0.10} \varnothing$	*Rate: MAK(k=0.1)*
$\mathrm{X}_1 + \mathrm{X}_3 \xrightarrow{0.73} \mathrm{X}_3$	*Rate: MAK(k=0.7332)*
$\mathrm{X}_2 + \mathrm{X}_3 \xrightarrow{0.76} \mathrm{X}_2 + \mathrm{X}_2$	*Rate: MAK(k=0.7588)*
$\mathrm{X}_3 + \mathrm{X}_3 \xrightarrow{5.88} \varnothing$	*Rate: MAK(k=5.8811)*

I/O CRN 5

Inputs: $\{u_1\}$ **Species:** $\{X_1, X_2, X_3\}$ **Output:** X_3

$\varnothing \xrightarrow{2.44} \varnothing$	*Rate: MAK(k=2.4425)*
$\varnothing \xrightarrow{u_1} \mathrm{X}_1$	*Rate: MAK(k=1.0,* u_1*)*
$\varnothing \xrightarrow{0.10} \mathrm{X}_2$	*Rate: MAK(k=0.1)*
$\mathrm{X}_1 \xrightarrow{0.10} \varnothing$	*Rate: MAK(k=0.1)*
$\mathrm{X}_1 \xrightarrow{12.26} \mathrm{X}_1 + \mathrm{X}_3$	*Rate: MAK(k=12.2619)*
$\mathrm{X}_2 \xrightarrow{0.10} \varnothing$	*Rate: MAK(k=0.1)*
$\mathrm{X}_3 \xrightarrow{0.10} \varnothing$	*Rate: MAK(k=0.1)*
$\mathrm{X}_1 + \mathrm{X}_3 \xrightarrow{0.54} \mathrm{X}_3$	*Rate: MAK(k=0.5446)*
$\mathrm{X}_1 + \mathrm{X}_3 \xrightarrow{0.28} \mathrm{X}_2 + \mathrm{X}_3$	*Rate: MAK(k=0.2842)*
$\mathrm{X}_2 + \mathrm{X}_3 \xrightarrow{0.46} \mathrm{X}_2 + \mathrm{X}_2$	*Rate: MAK(k=0.4598)*

I/O CRN 7

Inputs: $\{u_1\}$ **Species:** $\{X_1, X_2, X_3\}$ **Output:** X_3

$$\varnothing \xrightarrow{1.55} \varnothing \qquad \textit{Rate: MAK(k=1.5494)}$$
$$\varnothing \xrightarrow{u_1} \mathrm{X}_1 \qquad \textit{Rate: MAK(k=1.0, } u_1\textit{)}$$
$$\varnothing \xrightarrow{0.10} \mathrm{X}_2 \qquad \textit{Rate: MAK(k=0.1)}$$
$$\mathrm{X}_1 \xrightarrow{0.10} \varnothing \qquad \textit{Rate: MAK(k=0.1)}$$
$$\mathrm{X}_1 \xrightarrow{15.46} \mathrm{X}_1 + \mathrm{X}_3 \qquad \textit{Rate: MAK(k=15.4565)}$$
$$\mathrm{X}_2 \xrightarrow{0.10} \varnothing \qquad \textit{Rate: MAK(k=0.1)}$$
$$\mathrm{X}_3 \xrightarrow{0.10} \varnothing \qquad \textit{Rate: MAK(k=0.1)}$$
$$\mathrm{X}_1 + \mathrm{X}_3 \xrightarrow{1.53} \varnothing \qquad \textit{Rate: MAK(k=1.5272)}$$
$$\mathrm{X}_2 + \mathrm{X}_3 \xrightarrow{0.64} \mathrm{X}_2 + \mathrm{X}_2 \qquad \textit{Rate: MAK(k=0.6357)}$$
$$\mathrm{X}_3 + \mathrm{X}_3 \xrightarrow{0.97} \varnothing \qquad \textit{Rate: MAK(k=0.9733)}$$

7.2 Sensitization

I/O CRN 1

Inputs: $\{u_1\}$ **Species:** $\{X_1, X_2, X_3\}$ **Output:** X_3

$$\varnothing \xrightarrow{u_1} \mathrm{X}_1 \qquad \textit{Rate: MAK(k=1.0, } u_1\textit{)}$$
$$\varnothing \xrightarrow{0.10} \mathrm{X}_2 \qquad \textit{Rate: MAK(k=0.1)}$$
$$\mathrm{X}_1 \xrightarrow{0.10} \varnothing \qquad \textit{Rate: MAK(k=0.1)}$$
$$\mathrm{X}_2 \xrightarrow{0.10} \varnothing \qquad \textit{Rate: MAK(k=0.1)}$$
$$\mathrm{X}_2 \xrightarrow{0.44} \mathrm{X}_1 + \mathrm{X}_2 \qquad \textit{Rate: MAK(k=0.4427)}$$
$$\mathrm{X}_3 \xrightarrow{0.10} \varnothing \qquad \textit{Rate: MAK(k=0.1)}$$
$$\mathrm{X}_3 \xrightarrow{0.32} \mathrm{X}_2 + \mathrm{X}_2 \qquad \textit{Rate: MAK(k=0.3159)}$$
$$\mathrm{X}_1 + \mathrm{X}_3 \xrightarrow{0.14} \mathrm{X}_3 + \mathrm{X}_3 \qquad \textit{Rate: MAK(k=0.1404)}$$
$$\mathrm{X}_2 + \mathrm{X}_2 \xrightarrow{2.01} \mathrm{X}_1 \qquad \textit{Rate: MAK(k=2.0099)}$$
$$\mathrm{X}_2 + \mathrm{X}_2 \xrightarrow{0.87} \mathrm{X}_3 \qquad \textit{Rate: MAK(k=0.8678)}$$

I/O CRN 4

Inputs: $\{u_1\}$ **Species:** $\{X_1, X_2, X_3\}$ **Output:** X_3

$$\varnothing \xrightarrow{u_1} \mathrm{X}_1$$ *Rate: MAK(k=1.0, u_1)*

$$\varnothing \xrightarrow{0.10} \mathrm{X}_2$$ *Rate: MAK(k=0.1)*

$$\mathrm{X}_1 \xrightarrow{0.10} \varnothing$$ *Rate: MAK(k=0.1)*

$$\mathrm{X}_2 \xrightarrow{0.10} \varnothing$$ *Rate: MAK(k=0.1)*

$$\mathrm{X}_3 \xrightarrow{0.10} \varnothing$$ *Rate: MAK(k=0.1)*

$$\mathrm{X}_1 + \mathrm{X}_2 \xrightarrow{0.22} \varnothing$$ *Rate: MAK(k=0.2202)*

$$\mathrm{X}_1 + \mathrm{X}_2 \xrightarrow{0.17} \mathrm{X}_1 + \mathrm{X}_3$$ *Rate: MAK(k=0.1703)*

$$\mathrm{X}_1 + \mathrm{X}_3 \xrightarrow{0.11} \mathrm{X}_3 + \mathrm{X}_3$$ *Rate: MAK(k=0.1121)*

$$\mathrm{X}_2 + \mathrm{X}_3 \xrightarrow{0.10} \mathrm{X}_1$$ *Rate: MAK(k=0.0982)*

$$\mathrm{X}_2 + \mathrm{X}_3 \xrightarrow{0.53} \mathrm{X}_1 + \mathrm{X}_1$$ *Rate: MAK(k=0.5266)*

I/O CRN 7

Inputs: $\{u_1\}$ **Species:** $\{X_1, X_2, X_3\}$ **Output:** X_3

$$\varnothing \xrightarrow{u_1} \mathrm{X}_1$$ *Rate: MAK(k=1.0, u_1)*

$$\varnothing \xrightarrow{0.10} \mathrm{X}_2$$ *Rate: MAK(k=0.1)*

$$\mathrm{X}_1 \xrightarrow{0.10} \varnothing$$ *Rate: MAK(k=0.1)*

$$\mathrm{X}_1 \xrightarrow{0.48} \mathrm{X}_1 + \mathrm{X}_2$$ *Rate: MAK(k=0.4771)*

$$\mathrm{X}_1 \xrightarrow{0.42} \mathrm{X}_1 + \mathrm{X}_3$$ *Rate: MAK(k=0.4233)*

$$\mathrm{X}_2 \xrightarrow{0.10} \varnothing$$ *Rate: MAK(k=0.1)*

$$\mathrm{X}_3 \xrightarrow{0.10} \varnothing$$ *Rate: MAK(k=0.1)*

$$\mathrm{X}_1 + \mathrm{X}_1 \xrightarrow{0.22} \mathrm{X}_3$$ *Rate: MAK(k=0.2218)*

$$\mathrm{X}_1 + \mathrm{X}_2 \xrightarrow{1.13} \varnothing$$ *Rate: MAK(k=1.1293)*

$$\mathrm{X}_2 + \mathrm{X}_3 \xrightarrow{0.24} \mathrm{X}_1 + \mathrm{X}_1$$ *Rate: MAK(k=0.2366)*
